# Up-regulated NRIP2 in colorectal cancer initiating cells modulates the Wnt pathway by targeting RORβ

**DOI:** 10.1186/s12943-017-0590-2

**Published:** 2017-01-31

**Authors:** Zhenzhen Wen, Tianhui Pan, Saisai Yang, Jingwen Liu, Haiying Tao, Yiming Zhao, Dingting Xu, Wei Shao, Jia Wu, Xiyong Liu, Yongjiang Wang, Jianshan Mao, Yongliang Zhu

**Affiliations:** 1 Laboratory of Gastroenterology, Second Affiliated Hospital of Zhejiang University School of Medicine, Jiefang Road 88#, Hangzhou, Zhejiang 310009 China; 2 People’s Hospital of Huangyan district, Taizhou, Zhejiang 318020 China; 3 People’s Hospital of Putuo district, Zhoushan, Zhejiang 316100 China; 4 Department of Molecular Pharmacology, Beckman Research Institute, City of Hope, Duarte, CA 62232 USA; 5 Cancer Institute and Education Ministry Key Laboratory of Cancer Prevention and Intervention, Zhejiang University School of Medicine, Hangzhou, Zhejiang 310009 China; 6 Present address: Department of Gastroenterology, Sir Run Run Shaw Hospital of Zhejiang, University School of Medicine, Hangzhou, Zhejiang 310016 China

**Keywords:** Colorectal cancer initiating cells, Self-renewal, Non-canonical Wnt pathway, NRIP2, RORβ, HBP1

## Abstract

**Background:**

Colorectal cancer remains one of the most common malignant tumors worldwide. Colorectal cancer initiating cells (CCICs) are a small subpopulation responsible for malignant behaviors of colorectal cancer. Aberrant activation of the Wnt pathways regulates the self-renewal of CCIC. However, the underlying mechanism(s) remain poorly understood.

**Methods:**

Via retroviral library screening, we identified Nuclear Receptor-Interacting Protein 2 (NRIP2) as a novel interactor of the Wnt pathway from enriched colorectal cancer colosphere cells. The expression levels of NRIP2 and retinoic acid-related orphan receptor β (RORβ) were further examined by FISH, qRT-PCR, IHC and Western blot. NRIP2 overexpressed and knockdown colorectal cancer cells were produced to study the role of NRIP2 in Wnt pathway. We also verified the binding between NRIP2 and RORβ and investigated the effect of RORβ on CCICs both in vitro and in vivo. Genechip-scanning speculated downstream target HBP1. Western blot, ChIP and luciferase reporter were carried to investigate the interaction between NRIP2, RORβ, and HBP1.

**Results:**

*NRIP2* was significantly up-regulated in CCICs from both cell lines and primary colorectal cancer tissues. Reinforced expression of NRIP2 increased Wnt activity, while silencing of *NRIP2* attenuated Wnt activity. The transcription factor RORβ was a key target through which NRIP2 regulated Wnt pathway activity. RORβ was a transcriptional enhancer of inhibitor *HBP1* of the Wnt pathway. NRIP2 prevented RORβ to bind with downstream *HBP1* promoter regions and reduced the transcription of *HBP1*. This, in turn, attenuated the HBP1-dependent inhibition of TCF4-mediated transcription.

**Conclusions:**

NRIP2 is a novel interactor of the Wnt pathway in colorectal cancer initiating cells. interactions between NRIP2, RORβ, and HBP1 mediate a new mechanism for CCIC self-renewal via the Wnt activity.

**Electronic supplementary material:**

The online version of this article (doi:10.1186/s12943-017-0590-2) contains supplementary material, which is available to authorized users.

## Background

Colorectal cancer remains one of the most common malignant tumors in the world [1]. Studies have indicated that colorectal cancer consists of heterogeneous populations of cells differing in gene expression and growth capacities [2, 3]. CCICs are a small subpopulation of cells within colorectal tumors that can self-renew, differentiate into multiple lineages, and drive tumor growth [4, 5]. Among the CCIC properties, the self-renewal ability, which allows the cells to replicate is a crucial step for CCICs that is responsible for maintaining their homeostasis and malignant behaviors [6–8]. It is therefore of particular importance to clarify which molecules are abnormally activated during CCIC self-renewal.

There are several pathways participating in the regulation of the self-renewal of CCICs. Notch signaling plays an important role in promoting CCIC self-renewal. The Notch effector Hes1 up-regulates the expression of the stem-related molecules CD133, ABCG2, Nanog, and ALDH1 and increases the amount of CD133+ and stem-like SP cells within colorectal cancer cells [9]. miR-34a targeting *Notch1* promotes the differentiation of CCICs [10]; The BMP pathway maintains a stem cell self-renewal balance by inhibiting the Wnt pathway. The zinc-finger transcription factor GATA6 is a crucial regulation factor connecting the Wnt and BMP pathways. Competing with β-catenin/TCF4, GATA6 binds to a distal regulatory region of BMP4, decreases the threshold of the BMP pathway and enables the self-renewal of CCICs [11]. Colorectal cancer cells also have a high level of activity of HedgeHog (HH)-GLI signaling, and the self-renewal of CCICs relies on the direct function of HH-GLI activity in xenograft tumors [12]; Akt can activate 14-3-3zeta in the beta-catenin complex, which contributes to the stabilization and nuclear translocation of β-catenin, thus facilitating CCSC self-renewal by activating Wnt [13]. Akt also phosphorylates Oct4 to promote iPS factor transcription [14]. Among these pathways, the abnormal activation of the Wnt pathway is one of the most critical events in the tumorigenesis and development of colorectal cancer and plays a key role in maintaining the self-renewal of CCICs [15–18]. Aberrant activation of the Wnt pathway occurs in >90% of colorectal cancers [19]. Strong Wnt activation is found in cancerous intestinal epithelial ALDH^+^ initiating cells in ulcerative colitis in addition to CD133^+^ initiating cells in animal tumorigenesis models [20, 21]. Hence, Wnt activity is an important target for inhibition of the self-renewal of CCICs.

The Wnt pathways include canonical (β-catenin-dependent) and non-canonical (β-catenin-independent) pathways. In the canonical Wnt pathway, the ligands Wnt1, Wnt3a, and Wnt8 can bind the Frizzled receptor and LRP5/6 co-receptor on the cell surface, promoting the recruitment of Disheveled (Dvl) from the cytoplasm to the membrane. This, in turn, induces GSK-3β phosphorylation, which suppresses β-catenin degradation. Free β-catenin then accumulates in the cytoplasm and translocates to the nucleus, where it interacts with LEF and TCF to activate the transcription of downstream targets, including *MYC*, *CCND1*, *AXIN2,* and *LECT2*, etc. [22, 23]. The non-canonical pathways mainly include the PCP pathway and Wnt/Ca^2+^ pathway. In the PCP pathway, Wnt5a and other ligands bind to Frizzled and ROR2/PTK7 co-receptors, inducing a signaling cascade involving RhoA, Rac, Cdc42, and JNK, which act on the cytoskeleton [24, 25]. In the Wnt/Ca^2+^ pathway, Wnt ligands combine with Frizzled, leading to PKC and CamKII activation, which regulate target transcription [26, 27]. Thus, the non-canonical Wnt pathways maintain the self-renewal capacity of tumor cells and promote their tumorigenic ability by influencing canonical Wnt pathway activation at different levels [19, 26]. However, the mechanism of Wnt pathway activation in CCICs is still unclear; especially in terms of how non-canonical Wnt signaling molecules affect the canonical pathway.

Here, we identified NRIP2 as a novel molecule that collaborates with RORβ and HMG box-containing protein 1 (HBP1) to modulate Wnt activity.

## Methods

### Cancer tissues and cDNA database

All fresh primary colorectal cancer tissues were collected in the Second Affiliated Hospital of the Zhejiang University School of Medicine, with Institutional Review Board approval and informed consent provided by the patients (Reference number: R2014-041). All 565 cDNA genechip databases derived from patients with colorectal cancer were from the City of Hope National Medical Center of USA.

### Cell culture

Colorectal cancer SW620, HT29, and LoVo cells; gastric cancer SGC7901 cells; and 293 T cells were purchased from The Cell Bank of Chinese Academy of Sciences at the Shanghai Institute of Cell Biology. Colorectal cancer cells which were derived from primary colorectal cancer tissue were cultured in DMEM/F12 medium (Gibco, Gaithersburg, MD, USA) [28]; SW620 cells were cultured in L-15 medium (Gibco); HT29 cells were cultured in Macoy’s 5A medium (Gibco); Lovo cells were cultured in F-12 medium (Gibco); SGC7901 and P1 cells were cultured in RPMI-1640 medium (Cellgro, Manassas, USA); and 293 T cells were cultured in DMEM high-glucose medium (Gibco). All media were supplemented with 100 U/ml penicillin, 100 μg/ml streptomycin, and 10% fetal bovine serum (Gibco). The cells were cultured at 37 °C in a humidified atmosphere containing 5% CO_2_.

### Culturing and counting spheres

Colorectal cancer cell lines and primary colorectal cancer cells were seeded into 24-well low-adhesion plates (Corning, NY, USA) at 200 cells/well and cultured in serum-free sphere medium (containing 1× B27, 20 μg/L EGF, 20 μg/L bFGF, 4 mg/L insulin, 0.4% BSA, and 200 IU/mL streptomycin). These cells were grown in the presence or absence of Wnt Pathway Inhibitor XI, Wnt/β-catenin Inhibitor, Cardamonin (Merck, Germany), or the Wnt activator recombinant Wnt3a (R&D Systems, MN, USA) for 7–14 days at 37 °C in a humidified atmosphere containing 5% CO_2_. After the incubation period, the spheres were dilute passaged for an additional 1 week and the number of spheres was counted manually.

### Organoid culture

The above colosphere cells were digested with 0.25% trypsin and produced single cells using a 40 μM cell strainer (BD, USA). One-hundred cells in 40 μL of medium were mixed well with 70 μL of growth factor-deficient Matrigel (Biocoat, USA) and inoculated on the rim of a 24-well plate at 37 °C for 1 h. Subsequently, 1 mL of serum-free sphere medium was added for 5–7 days in a humidified atmosphere containing 5% CO_2_.

### Creation and screening of a retroviral cDNA library

Total RNA was extracted from SW620 colosphere cells using an RNeasy Kit (Qiagen, Germany). A ZAP cDNA Library Preparation Kit (Stratagene, CA, USA) was used to prepare cDNA, according to the manufacturer’s instructions. Briefly, RNA was reverse transcribed using a ZAP hemi-methylation primer. Next, double-stranded cDNA was synthesized in vitro, digested with XhoI/EcoRI endonucleases, cloned into the modified pLXSN vector (Clontech Laboratories, CA, USA), and transformed into *Escherichia coli* DH5α cells (Stratagene). The plasmids were then extracted and transfected into PT67 packaging cells (Clontech) to produce the recombinant retroviral particles. Before infection, CD133+ and CD44+ SGC7901 cells were removed by magnetic-activated cell sorting (Miltenyi, Germany), and the remaining SGC7901 cells were infected (multiplicity of infection is 20) and cultured in a serum-free low adhesion culture system for 7 days. Colospheres were then collected and digested into a single cell suspension and cloned by limiting dilution, and the clonal cells were further propagated. The Top/Fop flash reporter assay was used to determine Wnt activity. Genomic DNA from cells with obvious changes in Wnt activity was extracted using a DNA extraction kit (Qiagen, Germany), and PCR was used to amplify the inserted DNA fragment with primers from pLXSN plasmids. Finally, DNA sequencing was performed to verify the clones.

### mRNA hybridization

A QuantiGene @ ViewRNA ISH Tissue Assay Kit (Affymetrix, USA) was used for RNA hybridization according to the kit instructions. Briefly, *RORB* and *NRIP2* were used as TYPE1 probes, and *GAPDH* was used as a control probe. After staining the nucleus with DAPI or Hoechst 33342 dye (Invitrogen, Carlsbad, CA), the distribution and expression of *RORB* and *NRIP2* were observed under a confocal microscope (Carl Zeiss Jena, Germany).

### Assessing tumor sizes in mice

With approval from a local animal protection association, NOD/SCID and naked Balb/c mice were purchased from the Shanghai Laboratory Animal Center (Chinese Academy of Sciences) and bred in specific pathogen-free animal housing at the Laboratory Animal Research Center (Zhejiang Traditional Chinese Medical University). The mice were randomized into groups (5 mice/group for each tumor cell dose) and subcutaneously inoculated in their backs with 0.3 mL of different numbers of SW620 cells. The formation and growth of the transplanted tumors were observed, and the tumor sizes were recorded. The tumor volumes were calculated as 4/3π [1/2 × (long diameter/2 + short diameter/2)]^3^.

### Measuring Wnt pathway activity

Wild-type and mutant plasmids were co-transfected with Top/Fop flash reporters (Millipore, Germany) and the pRL plasmid as an internal reference (ratio of 3:1:0.1). The cells were harvested after 24–48 h, washed twice with phosphate-buffered saline (PBS), lysed in lysis buffer (Promega, Madison, WI, USA), and centrifuged at 13,000 rpm for 1 min. The luciferase activities were measured in the lysate supernatants using the Dual-Luciferase Reporter Assay System (Promega).

### Co-Immunoprecipitation and western blot analysis

For Co-IP studies, cells were harvested; incubated on ice for 15 min with 200 μl of RIPA lysis buffer containing 1% NP-40, 0.25% deoxycholic acid, 5 mM Dithiothreitol (DTT), and 1× protease inhibitor cocktail (Merck, NJ, USA); and centrifuged for 10 min at 13,000 rpm. The supernatants were collected and incubated with 5 μg of primary antibody for 2 h at 4 °C, then incubated overnight at 4 °C with 50 μl protein A/G-Agarose beads (Santa Cruz Biotechnology, Santa Cruz, CA, USA), and centrifuged at 13,000 rpm for 10 min. The beads were washed with lysis buffer and centrifuged 5 times. Subsequently, 50 μl of loading buffer was added to the beads and the samples were heated for 3 min in a water bath at 100 °C, cooled to room temperature (RT), and centrifuged for 1 min at 13,000 rpm. The resulting supernatants were collected for western blot analysis.

For western blot analysis, the cells were harvested and incubated on ice for 15 min with 200 μl of RIPA lysis buffer containing 1% NP-40, 0.25% deoxycholic acid, 5 mM DTT, and 1× protease inhibitor cocktail (Merck, NJ, USA). The lysates were centrifuged for 10 min at 13,000 rpm, and the supernatants were collected. The samples were mixed with 2× loading buffer, heated for 3 min in a 100 °C water bath, cooled to RT, and subjected to SDS-PAGE. The proteins were then transferred to a nitrocellulose membrane (Whatman, Dassel, Germany), blocked for 1 h at RT with Tris-base buffer saline +0.05% Tween 20 (TBST) buffer containing 5% skim milk, and then incubated with a primary antibody for 1 h at RT or overnight at 4 °C. Primary antibodies against the following target proteins were used in this study: NRIP2, HBP1 (1:1,000; Novus, USA), cyclin D1, c-Myc, RARα, RORβ (1:1000–2000; Epitomics, CA, USA), and GAPDH (1:5000; KangChen Biotech, Shanghai, China). The nitrocellulose membrane was washed with TBST and then incubated with a secondary antibody (HRP-labeled goat anti-rabbit antibody or HRP-labeled goat anti-mouse antibody, 1:2000, Cell Signaling Technology, Danvers, MA, USA) for 1 h at RT. Bands were visualized by exposing the membranes to ECL reagent (Cell Signaling Technology).

### Immunohistochemical staining

Following approval by the Ethics Committee of The Second Affiliated Hospital of the Zhejiang University School of Medicine, histological sections of colorectal cancer tissues were incubated overnight at 60 °C, fully hydrated with xylene and gradient alcohol, placed in antigen retrieval solution (pH 8.0, 100 mM EDTA), and heated for 15 min. Subsequently, the sections were cooled to RT, washed 3 times in TBST, blocked for 20 min at RT with TBST containing 10% goat serum, and washed 3 times in TBST. Sections were incubated overnight at 4 °C with an anti-NRIP2 antibody (1:1000, Novus, CO, USA), an anti-RORβ antibody (1:250, Novus, USA). After incubation with the primary antibody, the sections were washed 3 times in TBST and incubated for 1 h at RT with a secondary rabbit antibody (1:200; Dako, Denmark). The sections were developed with 3,3′-diaminobenzidine, counterstained with hematoxylin, and examined by microscopy.

### Lentivirus infection

For NRIP2 or RORβ over-expression, colorectal cancer cells were infected for 24 h with recombinant lentivirus encoding human *NRIP2* or *RORB* (Shanghai Innovation Biotechnology Co. for *NRIP2* and Shanghai Ruisai Biotechnology Co. for *RORB*); meanwhile, the cells were infected with blank vector lentivirus as a control. For target gene knockdown, colorectal cancer cells were infected for 72 h with lentivirus encoding shRNAs against *NRIP2*, *RORB*, or *HBP*1 or with scrambled shRNA as a control (Santa Cruz Biotechnology). Subsequently, cells were selected in puromycin (5 μg/ml) for 2 weeks. Clonal cells stably expressing shRNAs were cultured by limiting dilution, and the efficiency of target gene knockdown was verified by western blot analysis.

### Construction of *NRIP2* and *RORB* plasmids

The *NRIP2* ORF DNA sequence (Genbank access: AL136557) was synthesized by the Shanghai Xuguan Biotechnology Development Co. Ltd. and cloned into the pUC57 vector (Thermoscientific, MA USA). The *RORB*/pReceiver plasmid was purchased from Fulengen Co. Ltd (Guangzhou, China). Subsequently, the *NRIP2* and *RORB* ORF DNA sequences were subcloned into the *pEGFP-C1* vector at the XhoI and BamHI sites, respectively. Constructs were confirmed by DNA sequencing.

### RT-PCR and RT-qPCR

Total RNA was extracted from cells receiving different treatments using a RNA mini kit (Qiagen, Germany). After the quantity of RNA was checked, the RNA was reversed transcribed to cDNA by PrimeScript™ reverse transcriptase with a gDNA eraser kit (Takara, Japan). RT-PCR and Taqman RT-qPCR were carried out using the Premix EX Taq™ hot start version PCR and Perfect Real Time PCR kits following the manufacturer’s instructions. The primers used are as below: *NRIP2*: 5′-cacaggcacccaatacaatc-3′(Forward), 5′-tgtagctccaactgct ccac-3′ (Reverse), 5′Fam-ccaggcggctgagacatcca-3′Tamra (Probe); *RORB*: 5′-gcttcttattcctgcccaag-3′(Forward), 5′-cttggacatcctcccaaact-3′(Reverse), 5′Fam-aaccgttgccaacactgccg-3′Tamra (Probe); *GAPDH*: 5′-atcatccctgcc tctactgg-3′ (Forward), 5′-gtcaggtccaccactgacac-3′(Reverse), 5′Fam-accttgc ccacagccttggc-3′ Tamra (Probe).

### Gene chip detection

SGC7901 cells were transiently transfected with *RORB*/pReceiver plasmids (Fulengen) using Lipofectamine 2000 reagent (Invitrogen, USA) for 48 h. The cells were lysed by the addition of 1 mL of Trizol reagent (BBI, Canada), and total RNA was extracted. A GeneChip® PrimeView™ Human Gene Expression Array (Affymetrix, USA) was used to detect global mRNA expression profiles. Differences in mRNA expression were verified by RT-qPCR.

### Chromatin immunoprecipitation

A commercial kit (Upstate, Millipore, USA) was used to perform ChIP assays according to the manufacturer’s instructions. In brief, SGC7901 cells were seeded in a 100-mm dish at 70% confluency overnight and subsequently transiently transfected with *RORB*/pReceiver plasmids for an additional 48 h. Transfected cells were fixed by a final concentration of 1% formaldehyde for 10 min; the reaction was terminated by adding 0.5 mL of 1 M glycine solution. The cells were collected and lysed by SDS reagent. The DNA fragments were co-immunoprecipitated by anti-myc tag antibodies in agarose at 4 °C overnight after sonication. The immunoprecipitated DNA fragments were purified and eluted by the spin filter method. PCR was used for the detection of the *HBP1* upstream DNA fragments with the primers: Forward:5’-gtcctttgcaggacatggat-3’, Reverse: 5’-gtgccatggaggtttgctat-3’. Blank, normal mouse IgG was used as a negative control, and anti-RNA polymerase II was used as a positive control.

### EMSA

An Electrophoretic Mobility Shift Assay (EMSA) kit (Pierce, Thermo Scientific, USA) was used to perform EMSA assays according to the manufacturer’s instructions. DNA sequences of the wild-type and mutant hormone response elements were chemically synthesized. Upstream and downstream DNA primers (100 μM) were mixed and incubated at 94 °C in vitro for 5 min and allowed to cool to RT. The probe was incubated with recombinant RORβ for 30 min and resolved on a 6% PAGE gel. The DNA was then transferred to a nylon membrane and crosslinked for 1 min, after which HRP-labeled streptavidin was added for 30 min and an enhanced ECL reagent was used for detection. WT: 5’-Bio-gatcacgaggtcaagagatcgagaccatcctg-3’(Forward), 5’-Bio-caggatggtctcgatctcttgacctcgtgatc-3’ (Reverse); Mut1:5’-Bio-gatcacg aggtcaagaccatcctg-3’ (Forward), 5’-Bio-caggatggtcttgacctcgtgatc-3’ (Reverse); Mut2: 5’-Bio-gatcacgaggtcaagagatcgtcctg-3’ (Forward), 5’-Bio-caggacgatct cttgacctcgtgatc-3’ (Reverse); Mut3: 5’-Bio-gatcacgagagatcgagaccatcctg -3’ (Forward), 5’-Bio-caggatggtctcgatctctcgtgatc-3’ (Reverse).

### Construction and transfection of the *HBP1* promoter/pGL3 luciferase reporter vector

Bioinformatics analysis of the human *HBP1* 5′ flanking region was used to design the following PCR primers: 5′-agtcactgagctctgtcccagacaccaaaacaa-3′ (forward) and 5′-agtcactctcgagcggcagctcttactcctcaa-3′ (reverse). DNA from SW620 cells was used as a template. The ~2,500 bp promoter region of the *HBP1* gene was amplified using 35 cycles of 95 °C for 10 s, 55 °C for 15 s, and 72 °C for 150 s. The PCR product was doubly digested with XhoI and SacI enzymes and inserted into the pGL3-basic vector (Promega), creating a luciferase reporter gene vector containing the *HBP1* promoter region (pGL3-*HBP*1). The primers used to construct the *HBP1* gene promoter reporter vector containing hormone response elements were as follows: 5′-agtcactctcgagcggcagctcttactcctcaa-3′(forward) and 5′-agtcactgagc tcggaggatcacgaggtcaagagatcgagaccatcctggtgtcccagacaccaaaacaa-3′ (reverse). Plasmid DNA containing the *HBP1* promoter was used as a template for PCR, with 10 cycles of (95 °C for 10 s, 58.8 °C for 15 s, and 72 °C for 40 s) and 20 cycles of (95 °C for 10 s, 69.0 °C for 15 s, and 72 °C for 40 s). The PCR product was doubly digested with XhoI and SacI enzymes and inserted into the pGL3-basic vector, creating a *HBP1* promoter reporter vector containing hormone response elements.

The pGL3-*HBP*1, *RORB*-pCMV6/XL4, and pRL plasmids (an internal reference) were co-transfected (1:3:0.1) by Lipofectamine 2000 reagent into SGC7901 tumor cells for 24 h. The cells were collected and washed twice in 0.01 M PBS (pH7.4) and lysed in lysis buffer (Promega, Madison, WI, USA). The cell lysates were centrifuged at 13,000 rpm for 1 min, and the supernatants were collected for the measurement of luciferase activity. The pGL3-basic, pCMV6/XL4, and pRL plasmids were co-transfected (1:3:0.1) in control experiments.

### Statistical analysis

Continuous variables were expressed as the mean ± standard deviation (SD). Single factor analysis of variance and *t* tests were used to compare multiple groups of independent samples or paired samples. The level of significance was set as *p* <0.05. Statistical analysis was performed using SPSS 18 (SPSS Inc., Chicago, IL, USA).

## Results

### Wnt activity is important for the self-renewal of CCICs

**Fig. 1:**
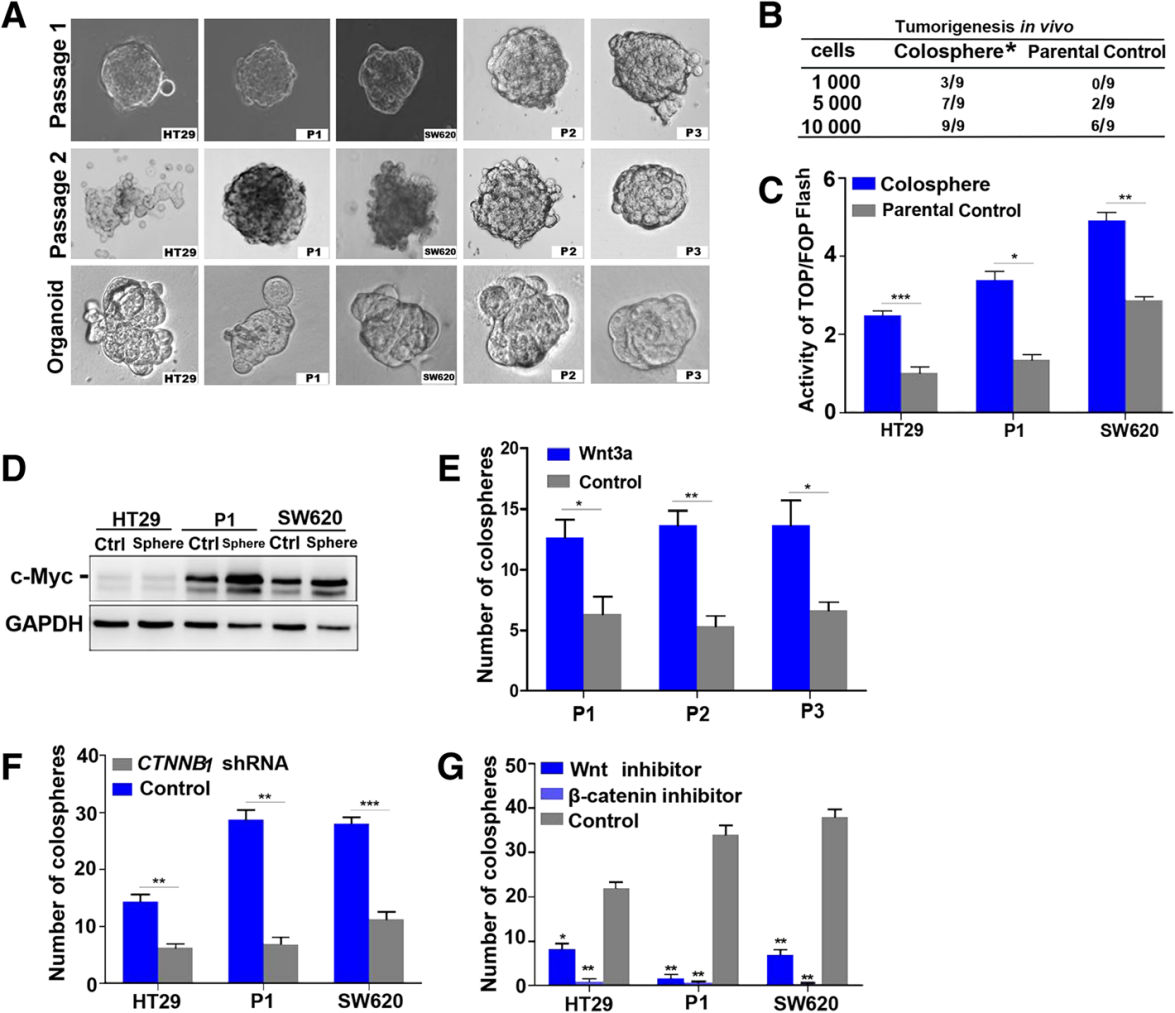
Wnt activity is important for the self-renewal of CCICs. **a** Colospheres were enriched from 3 primary colorectal cancer tissues, colorectal cancer HT29 cells, and SW620 cells on the 5th day in low-adhesive and serum-free culture medium. Colosphere formation occurred after the serial dilution of cells on the 5th day. Single colosphere cells formed organoids in growth factor-deficient Matrigel medium at the 5-7th day (bottom). **b** Tumorigenicity of colospheres. Different numbers of colosphere cells from primary colorectal cancer tissues (P1 cells) were injected into NOD/SCID mice and tumor formation was quantified after 8 weeks. The results showed that colospheres exhibited significantly increased tumorigenicity (*p* < 0.05, Multivariate logistic analysis). The same number of parental cells was used as a control. **c** Wnt activity in colospheres. Colosphere or parental cells (control) were transfected with Top/Fop flash reporters, and the Wnt pathway activity was determined at 24 h post-transfection. The fold-change was calculated relative to controls. The values are represented as the mean ± SD from triplicate samples. *p < 0.05; **p < 0.01; ***p < 0.001 (ANOVA). **d** The levels of Wnt signaling downstream of c-Myc were detected by western blotting in HT29, P1 and SW620 colospheres (Spheres), with their parental cells as controls. **e** Quantification of organoid formation after Wnt pathway activation. The number of organoids was counted from 3 primary colorectal cancer cells (100 cells/well) by the treatment of recombinant Wnt3a (200 ng/mL) for 7 days; cells without treatment of Wnt3a were used as controls. The number of organoids significantly increased after Wnt pathway activation. *p < 0.05 (ANOVA). **f** Quantification of colosphere formation after *CTNNB1* knockdown. The colosphere number was counted in *CTNNB1-*knockdown HT29, P1, and SW620 cells under low-adhesive and serum-free conditions for 7 days. The colosphere number significantly decreased in these cells after knockdown of *CTNNB1*. **p < 0.01; ***p < 0.001 (ANOVA). **g** Quantification of colosphere formation after Wnt pathway inactivation. Colospheres were counted in HT29, P1, and SW620 cells treated with Wnt inhibitor (7.2 μM) or β-catenin inhibitor (3.6 μM) for 7 days, with the solvent dimethyl sulphoxide (DMSO) as a control. The colosphere number significantly decreased after the inhibition of Wnt activity. *p < 0.05; **p < 0.01; (ANOVA)

To evaluate the effect of Wnt activity on CCIC self-renewal, we first enriched colospheres in vitro from primary colorectal cancer tissues and colorectal cancer cell lines and identified their stem-like properties. Using serum-free, low-adhesion culture conditions, colospheres were successfully enriched and dilute passaged from 3 primary colorectal cancer cells as well as colorectal cancer-derived HT29 and SW620 cells. Furthermore, single cells from these spheres were able to form organoids in the conditioned Matrigel medium (Fig. 1a). These colospheres were inoculated into NOD/SCID mice, and they exhibited significantly increased tumorigenicity (Fig. 1b). These results suggested that the enriched colospheres possessed cancer-initiating cell properties. Next, we assayed for Wnt activity in the colosphere cells. Both Top/Fop flash reporter assays and western blots indicated that these colosphere cells had relatively strong Wnt activity compared with their parental cells (Fig. 1c and d). Finally, we evaluated the effect of Wnt pathway activation on the self-renewal capacity of CCICs. The number of colospheres was obviously increased after Wnt signaling was activated by the addition of recombinant Wnt3a (Fig. 1e). However, the number of colospheres significantly decreased in primary colorectal cancer P1, HT-29, and SW620 cells after *CTNNB*1 was knocked down by RNA interference (Fig. 1f). Treatment with Wnt and β-catenin chemical inhibitors showed similar results (Fig. 1g). Collectively, these results suggested that activation of the Wnt pathway plays an important role in the self-renewal capacity of CCICs.

### NRIP2 is significantly up-regulated in CCICs

**Fig. 2:**
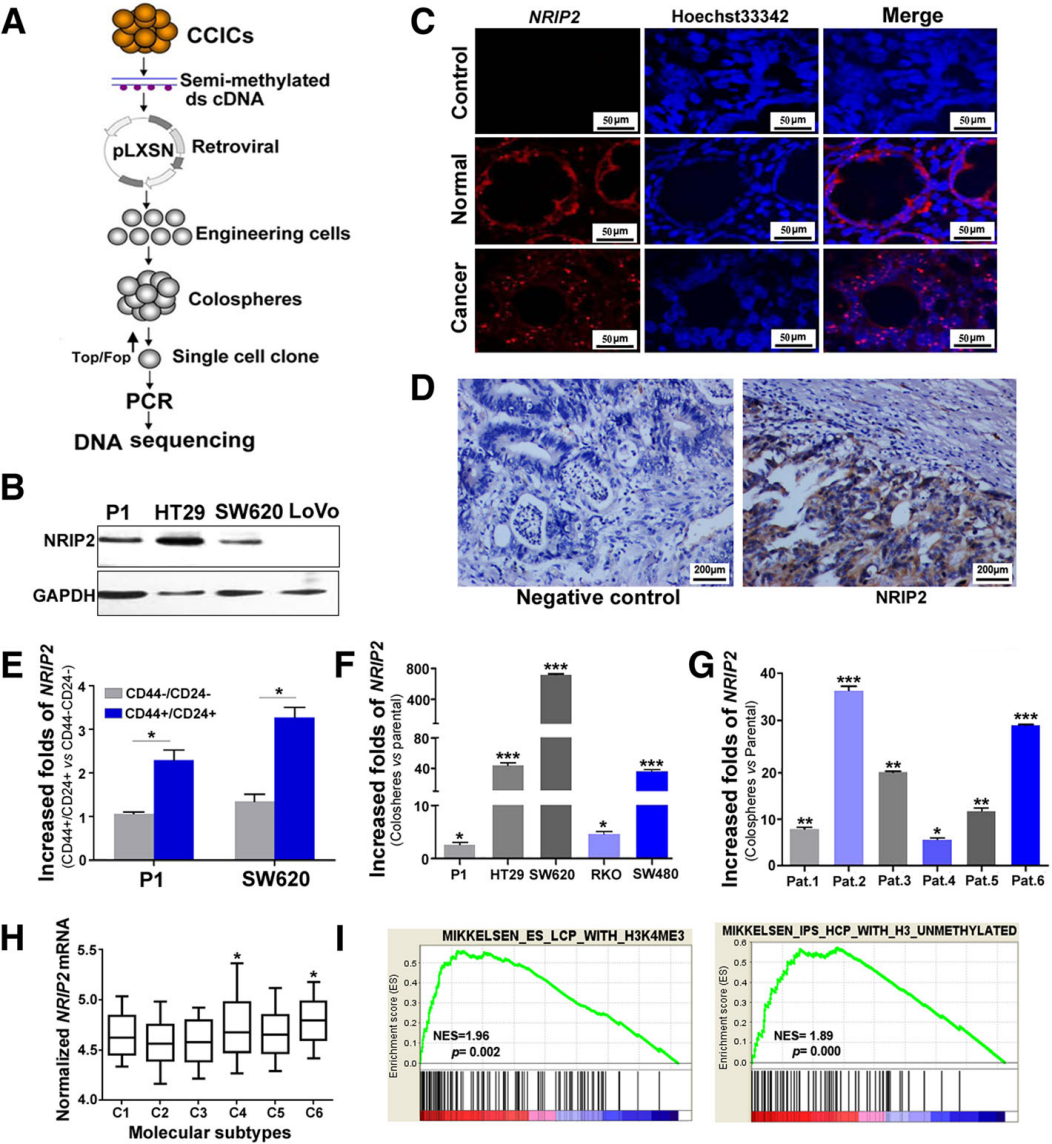
NRIP2 is significantly upregulated in CCICs. **a** Screening strategy for *NRIP2. NRIP2* was identified in a screen for colosphere formation using a retroviral cDNA expression library constructed from SW620 colosphere RNA (as detailed in the Materials and Methods). **b** NRIP2 expression in colorectal cancer cells. NRIP2 was detected by western blotting in colorectal cancer cells. The results showed that P1, HT29, and SW620 cells expressed NRIP2. **c** *NRIP2* mRNA hybridization. Slides containing primary colorectal cancer tissue and normal colorectal tissue were hybridized with labeled probes for *NRIP2* mRNA respectively, with a nonsense probe as a negative control. FISH analysis showed *NRIP2* expression in primary colorectal cancer cells. **d** Expression of NRIP2 in primary colorectal cancer tissue. Colorectal cancer cells were reacted with antibodies against NRIP2 and subsequently detected by IHC staining. Normal rabbit IgG was used as a negative control. **e** *NRIP2* increased in CD44 + CD24+ cancer cells. *NRIP2* mRNA levels were determined by Taqman RT-qPCR in CD44 + CD24+ and CD44-CD24- FACS-isolated cells. *NRIP2* expression increased in CD44 + CD24+ cells compared with CD44-CD24- cells; *p < 0.05 (ANOVA). **f** *NRIP2* expression in colospheres from colorectal cancer cells. *NRIP2* levels were determined by Taqman RT-qPCR in colospheres from P1, HT29, RKO, and SW620 cells under low-adhesive and serum-free conditions for 7 days. *NRIP2* mRNA expression was significantly higher in colospheres than in parental cells. *p < 0.05; ***p < 0.001 (ANOVA). **g** *NRIP2* expression in colospheres from primary colorectal cancer cells. *NRIP2* levels were determined by Taqman RT-qPCR in colospheres from primary colorectal cancer samples under low-adhesive and serum-free conditions for 7 days. *NRIP2* mRNA expression was significantly higher in colospheres than in their parental cells. *p < 0.05; **p < 0.01; ***p < 0.001 (ANOVA). **h** Analysis of the relationship between *NRIP2* and the colorectal cancer subtypes. The relationship between *NRIP2* expression and colorectal cancer molecular typing was analyzed by the global cDNA expression GeneChip database (n = 565). *NRIP2* expression was closely related to the C4-cancer stem cell (CSC) and C6-CIN colorectal cancer subtypes. C4 *vs* C2 or C3, all *p* < 0.05; C4 *vs* C2 or C3, all *p* < 0.01. **i** GSEA analysis. GSEA analysis from primary colorectal cancer tissues with a high level of *NRIP2* expression (n = 200). Similar alterations in the mRNA expression profiles were found in cases with high levels of *NRIP2* expression and in ES cells with histone methylation or iPS cells without histone methylation

The above results demonstrated that Wnt pathway activation plays an important role in maintaining the self-renewal of CCICs; however, the molecular mechanisms whereby Wnt pathway activation occurs in CCICs remain unclear. To screen molecular activators of the Wnt pathway, we constructed a retroviral cDNA library from SW620 colosphere cells and screened it by colosphere formation and Top/Fop flash reporter assays as well as DNA sequencing (Fig. 2a). Based on these screening strategies, there were 13 candidates from SW620 colosphere cells identified by DNA sequencing (Additional file 1: Figure S2a). We found that among these candidates, only the function of *NRIP2* was undefined for promoting the self-renewal of colosphere cells.

Western blot analysis showed that NRIP2 was expressed in primary colorectal P1, HT29, and SW620 cells (Fig. 2b, Additional file 1: Figure S2b). The presence of NRIP2 in primary tumor cells was confirmed by mRNA fluorescence in situ hybridization (FISH) and immunohistochemical (IHC) staining (Fig. 2c and d). To verify that NRIP2 is expressed at a higher level in CCICs, we isolated CD44^+^CD24^+^ colorectal cancer-initiating cells from primary colorectal P1 and SW620 cells by fluorescence-activated cell sorting (FACS) and colospheres from colorectal cancer cell lines and primary colorectal cancer tissues. RT-qPCR analysis showed that *NRIP2* expression was significantly higher in CD44^+^CD24^+^ cells and colosphere cells (Fig. 2e-g). Similar results were observed in CD133^+^ and Aldefluor^+^ colorectal cells (Additional file 1: Figure S2c and S2d). On the other hand, we analyzed the relationship between *NRIP2* expression and colorectal cancer molecular typing in 565 cases of colorectal cancer from a global cDNA expression genechip database and found that high *NRIP2* expression was closely related to the C4-cancer stem cell (CSC) subtype of colorectal cancer (Fig. 2h) [29]. Gene set enrichment analysis (GSEA) also showed that in colorectal cancer cells expressing high levels of *NRIP2*, the alteration of the mRNA expression profiles was similar to that observed with low- and intermediate-CpG-density promoters bearing the histone H3 trimethylation marker at K4 and K27 (H3K4me3 and H3K27me3) in embryonic stem cells (ES) and induced pluripotent cells (iPS) (Fig. 2i). These results suggested that NRIP2 may play an important role in the self-renewal of CCICs.

### NRIP2 up-regulates Wnt pathway activity

**Fig. 3:**
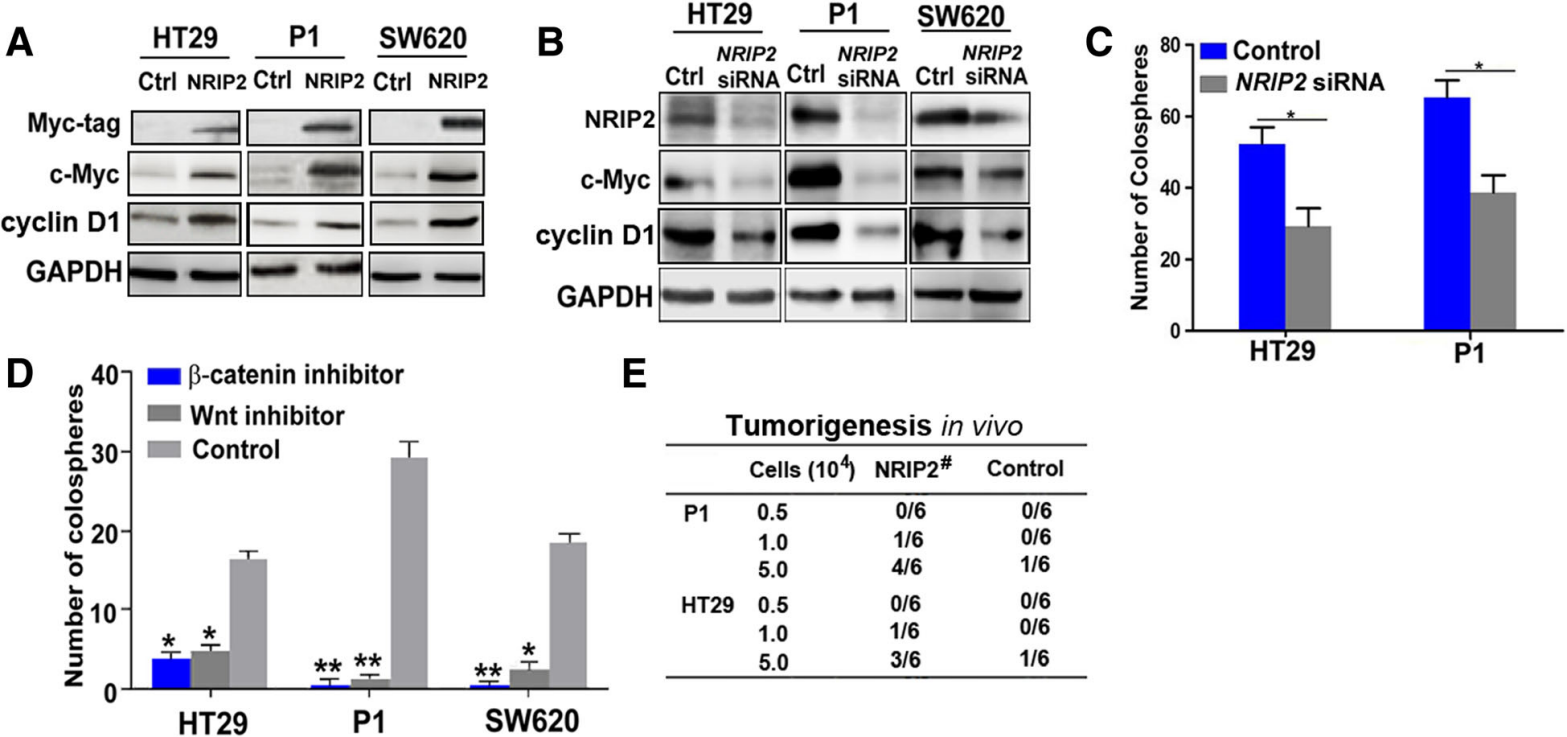
NRIP2 up-regulates Wnt pathway activity. **a** Over-expression of NRIP2 up-regulates Wnt pathway activity. c-Myc and cyclin D1 were detected by western blotting in HT29, P1, and SW620 cells infected with recombinant lentivirus encoding *NRIP2* with Myc-Tag, with cells infected with blank lentivirus as controls. **b** Wnt activity in NRIP2-knockdown cells. c-Myc and cyclin D1 were detected by western blotting in NRIP2-knockdown HT29, P1, and SW620 cells and their scrambled *shRNA*-treated cells (controls). **c** Quantification of colospheres after *NRIP2* silence. The number of colospheres was counted in Nrip2-expressing HT29 and P1 cells after *NRIP2* silence. The number of colospheres significantly decreased after silence of NRIP2, *p < 0.05 (ANOVA). Transfection with RNAi scramble as controls. **d** Quantification of colospheres in NRIP2-overexpressing cells after Wnt inactivation. Colospheres were counted in NRIP2-overexpressing HT29, P1, and SW620 cells and controls treated with Wnt (7.2 μM) and β-catenin inhibitors (3.6 μM). The colosphere number significantly decreased in NRIP2-overexpressing cells after Wnt inactivation. *p < 0.05; **p < 0.01 (ANOVA). **e** Tumorigenicity of NRIP2- overexpressing cells. NRIP2- overexpressing P1 and HT29 cells and their control cells infected with blank lentivirus were injected into naked Balb/c mice, respectively. Tumor formation was quantified within 4 weeks. The results showed that overexpression of NRIP2 significantly increased tumorigenicity (# *p* < 0.05, Multivariate logistic analysis)

To characterize the relationship between NRIP2 and Wnt activity, we determined the impact of NRIP2 on Wnt activity in HT29, P1 and SW620 cells. Western blots showed that the Wnt pathway downstream targets were significantly increased in cells after overexpression of NRIP2, while it was obviously decreased after silencing of *NRIP2* in these cells (Fig. 3a and b, Additional file 1: Figure S3). Furthermore, the colosphere numbers were significantly attenuated in NRIP2- overexpressing HT29 and P1 cells after silencing of *NRIP2* (Fig. 3c). Finally, we evaluated whether NRIP2 promoted the self-renewal of CCICs dependent on Wnt activation. To this end, we observed a change in the colosphere number in NRIP2-overexpressing cells after inactivation of Wnt. The results showed that NRIP2 overexpression did not reverse the inhibition caused by Wnt- and β-catenin chemical inhibitors (Fig. 3d). NRIP2 overexpressing cells were inoculated into NOD/SCID mice, and they exhibited significantly increased tumorigenicity (Fig. 3e). Together, these findings suggested that NRIP2 involves in the self-renewal of colorectal cancer cells by activating the Wnt pathway.

### NRIP2 regulates the activity of the Wnt pathway via RORβ

**Fig. 4:**
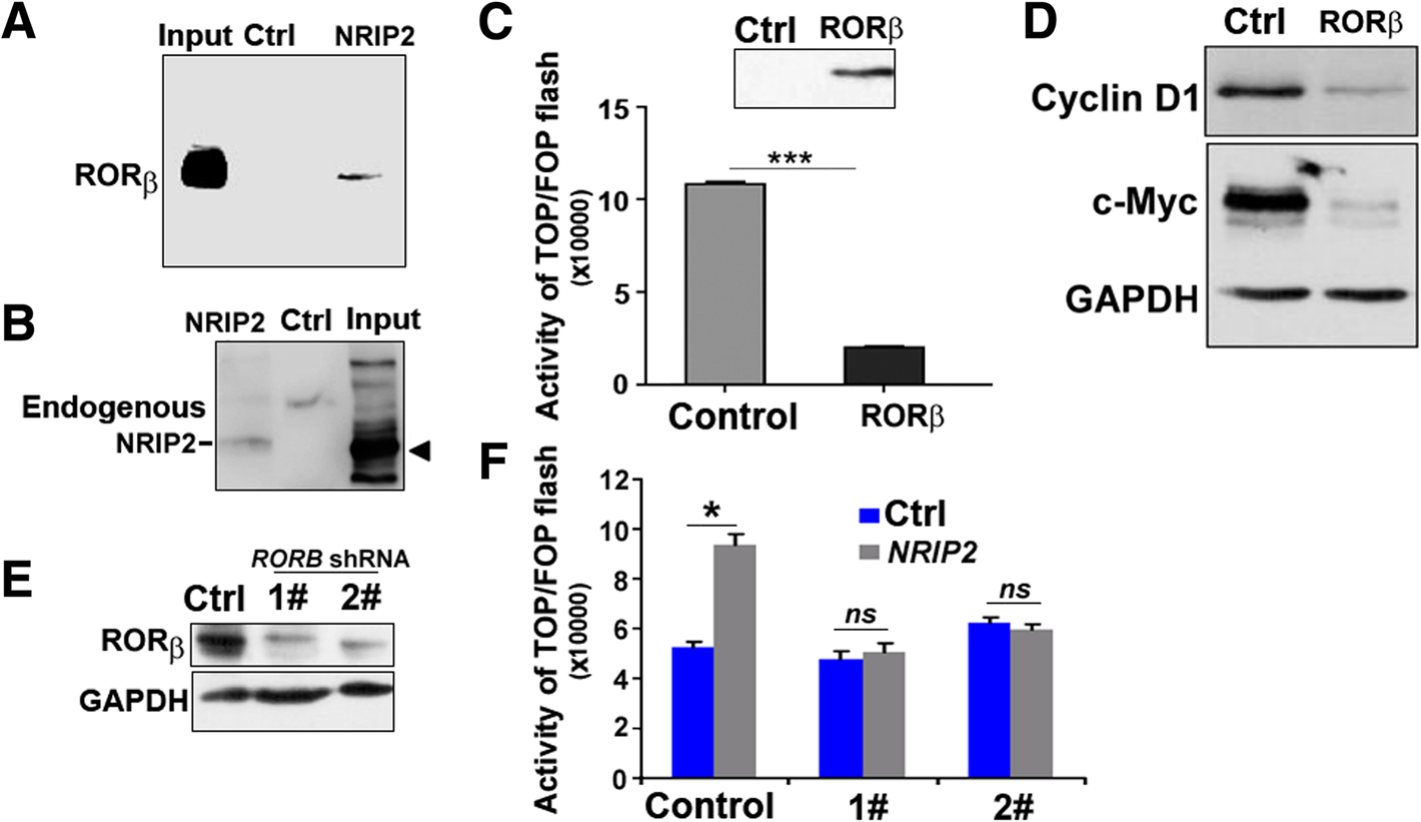
NRIP2 regulates the activity of the Wnt pathway via RORβ. **a** Analysis of NRIP2 binding to RORβ. Lysates from NRIP2-overexpressing SGC cancer cells or control cells were subjected to co-IP with myc-tag antibody agarose beads, followed by western blotting with anti-RORβ antibodies. Normal rabbit IgG agarose beads were used as a negative control. **b** Endogenous NRIP2 interacts with RORβ. Lysates from P1 cells without treatment with protease inhibitor cocktails were subjected to co-immunoprecipitation with RORβ antibodies or mouse IgG (control), followed by western blotting with anti-NRIP2 antibodies. Normal rabbit IgG agarose beads were used as a negative control. **c** Wnt activity in RORβ-expressing cells. Wnt activity was assessed by Top/Fop flash reporter assays in SGC7901 cells 24 h after transient transfection with *RORB* or control plasmids. Overexpression of RORβ attenuated Wnt activity. *p < 0.05 (ANOVA). **d** Detection of Wnt dowmstream targets in RORβ-expressing cells. c-Myc and cyclin D1 were detected by western blotting in the above RORβ-overexpressing SGC7901 and control cells. **e** Detection of RORβ in *RORB-*knockdown cells. Cells were infected with lentivirus encoding *RORB* shRNA for 72 h and subsequently screened with 5 μg/mL Puromycin for 7 days. The surviving cell clone was picked out with limiting dilution analyses. RORβ was detected by western blotting in these *RORB-*knockdown clones, with cells infected with scrambled shRNA lentivirus as controls. **f** NRIP2 failed to activate the Wnt pathway in *RORB-*knockdown cells. Wnt activity was assessed by Top/Fop flash reporter assays in *RORB-*knockdown SGC7901 cell clones 24 h after transient transfection with *NRIP2* or control plasmids. NRIP2 could not activate the Wnt pathway after knockdown of *RORB*. *p < 0.05 (ANOVA), *ns*: non-significance, *p* > 0.05 (ANOVA)

To discover downstream target molecules by which NRIP2 regulates the Wnt pathway, we performed a literature review and target prediction (www.genecards.org) and found that NRIP2 can interact with RORβ [30]. Thus, we performed Co-IP and western blot analysis with cells overexpressing NRIP2 and RORβ. The results confirmed that both exogenous and endogenous NRIP2 could be co-immunoprecipitated with RORβ (Fig. 4a and b), but NRIP2 could not bind to RARα (Additional file 1: Figure S4). These results suggest that NRIP2 may be involved in the Wnt pathway.

Although NRIP2 can interact with RORβ, it is not clear whether RORβ affects Wnt pathway activity. Therefore, we transiently expressed RORβ in SGC7901 cells, which had the highest transfection efficiency. The results from Top/Fop flash assays and western blots showed that the activity of the Wnt pathway was significantly inhibited by RORβ overexpression (Fig. 4c and d). To determine whether NRIP2 activates the Wnt pathway dependent on RORβ, we established *RORB*-knockdown cells and checked the effect of NRIP2 on these cells. The results showed that NRIP2 could not activate the Wnt pathway after knockdown of *RORB* (Fig. 4e and f). Together, these results suggested that RORβ is an inhibitor of the Wnt pathway and that NRIP2 may affect Wnt pathway activity via RORβ.

### RORβ inhibits tumorigenesis and the self-renewal of CCICs

**Fig. 5:**
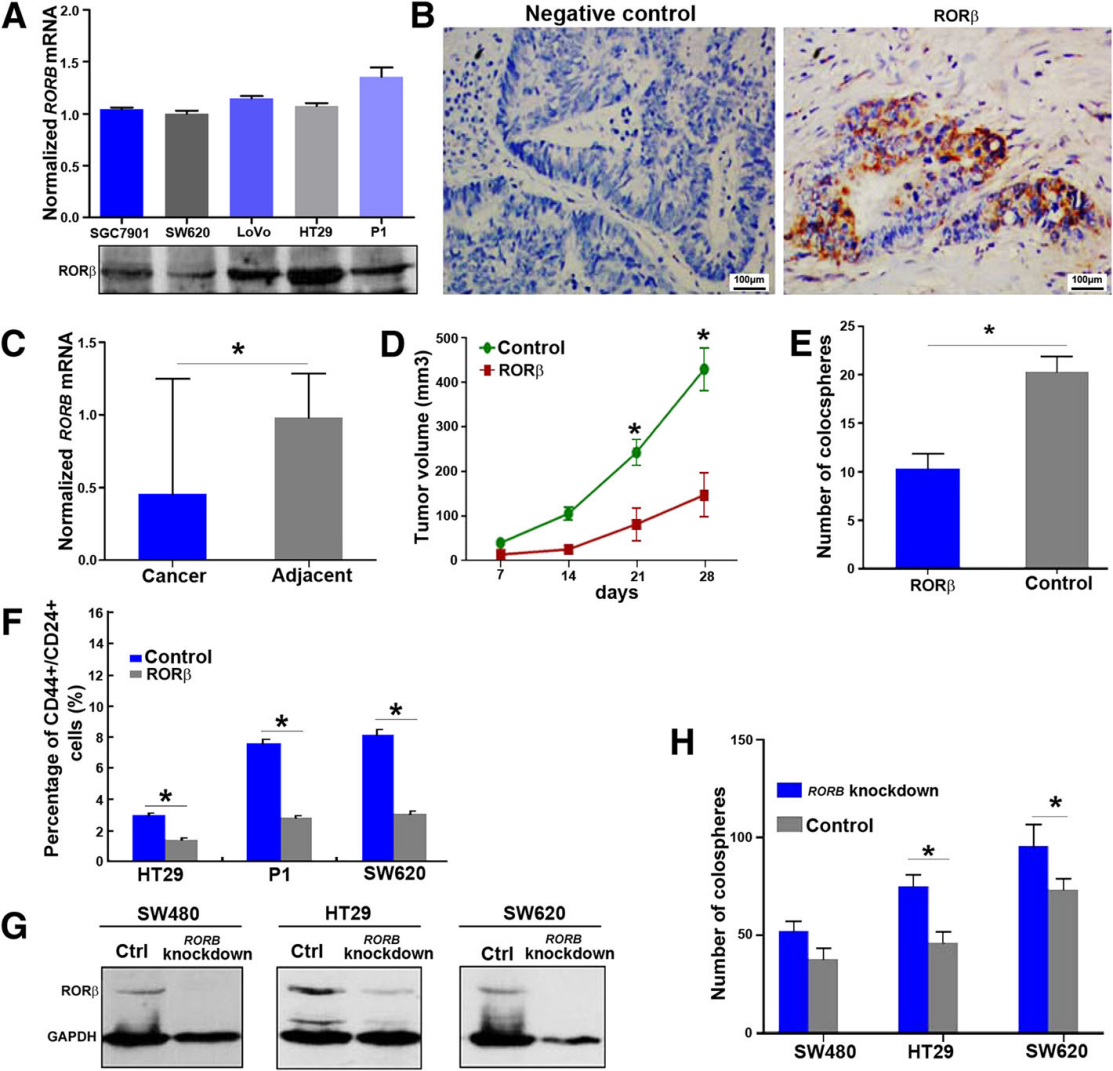
RORβ inhibits tumorigenesis and the self-renewal of CCICs. **a** *RORB* expression in colorectal cancer cells. *RORB* mRNA and protein expression levels were detected in colorectal cancer cells by Taqman RT-qPCR and western blotting, respectively. *RORB* mRNA was normalized with *GAPDH*. **b** RORβ expression in primary colorectal cancer tissues. RORβ expression in human primary colorectal cancer tissues was detected by IHC staining with antibodies against RORβ. Normal rabbit IgG was used as a negative control. **c** *RORB* mRNA in primary colorectal cancer tissues. *RORB* mRNA was measured by Taqman RT-qPCR in 14 patients with colorectal cancer. The results showed that the levels of *RORB* in colorectal cancer cells were significantly lower than those in matched adjacent tissues. *p < 0.05 (ANOVA). **d** Tumorigenicity of RORβ-overexpressing cells. RORβ- overexpressing SW620 cells (1 × 10^6^) as well as their control cells infected with blank lentivirus were injected into naked Balb/c mice, respectively (n = 5). Tumor formation was quantified within 4 weeks. *p < 0.05 (ANOVA). The results showed that RORβ inhibited tumor growth. **e** Quantification of colospheres in RORβ-overexpressing cells. Colospheres were counted in RORβ-overexpressing P1 and control cells infected with blank lentivirus at the 5th day under low-adhesion and serum-free condition. The number of colospheres significantly decreased after RORβ overexpression compared with the controls. *p < 0.05 (ANOVA). **f** Determination of the percentage of CD44 + CD24+ cells after overexpression of RORβ. The percentage of CD44 + CD24+ cells were analyzed by FCM in RORβ-overexpressing HT29, P1 and SW620 cells, with cells infected with blank lentivirus as controls. The results showed that RORβ reduced the percentage of CD44 + CD24+ cells compared with the control cells, all *p* < 0.05 (ANOVA). **g** RORβ expression in *RORB-*knockdown cells. Cells were infected with lentivirus encoding *RORB* shRNA for 72 h and subsequently screened with 5 μg/mL Puromycin for 7 days. The surviving cell clone was picked out with limiting dilution analyses. RORβ was detected by western blotting in these *RORB-*knockdown clones, with cells infected with scrambled shRNA lentivirus as controls. **h** Quantification of colospheres in *RORB-*knockdown cells. Colospheres from the above *RORB*-knockdown colorectal cancer cell clones were counted at day 5 under serum-free conditions. The number of colospheres was significantly higher in *RORB*-knockdown cells than in the control cells. *p < 0.05 (ANOVA)

Previous studies have demonstrated that RORβ is mainly distributed in the central nervous system [31, 32], however, whether RORβ is also expressed in intestinal epithelial cells is unverified. To confirm that RORβ is expressed in intestinal epithelial tissue, we evaluated RORβ expression in colorectal cancer cells by RT-qPCR and western blotting. Immunostaining of RORβ was also carried out in human primary colorectal cancer tissues. We also examined *RORB* mRNA expression in primary colorectal cancer tissues by RT-qPCR and mRNA FISH. We found that *RORB* was expressed in colorectal cancer cells, but at a lower level in colorectal cancer tissue than in matched para-carcinoma tissues (Fig. 5a-c, Additional file 1: Figure S5). The level of *RORB* in CCICs was not significantly different from their parental cells (data not shown). To further clarify the effect of RORβ on CCICs, we observed the tumorigenicity in vivo, the change in the colosphere number and the ratios of CD44 + CD24+ cancer-initiating cells in cells with overexpression of RORβ. The results showed that the tumorigenic capacity was significantly reduced (Fig. 5d), the sphere-forming efficiency was decreased and the ratio of CD44^+^CD24^+^ cells and the number of colospheres were also obviously reduced in colorectal cancer cells after overexpression of RORβ (Fig. 5e and f). Inversely, *RORB* knockdown led to increased colosphere formation (Fig. 5g and h). These results suggested that RORβ negatively regulates the self-renewal of CCICs as a suppressor.

### HBP1 is a crucial target of RORβ in regulation of the Wnt pathway

**Fig. 6:**
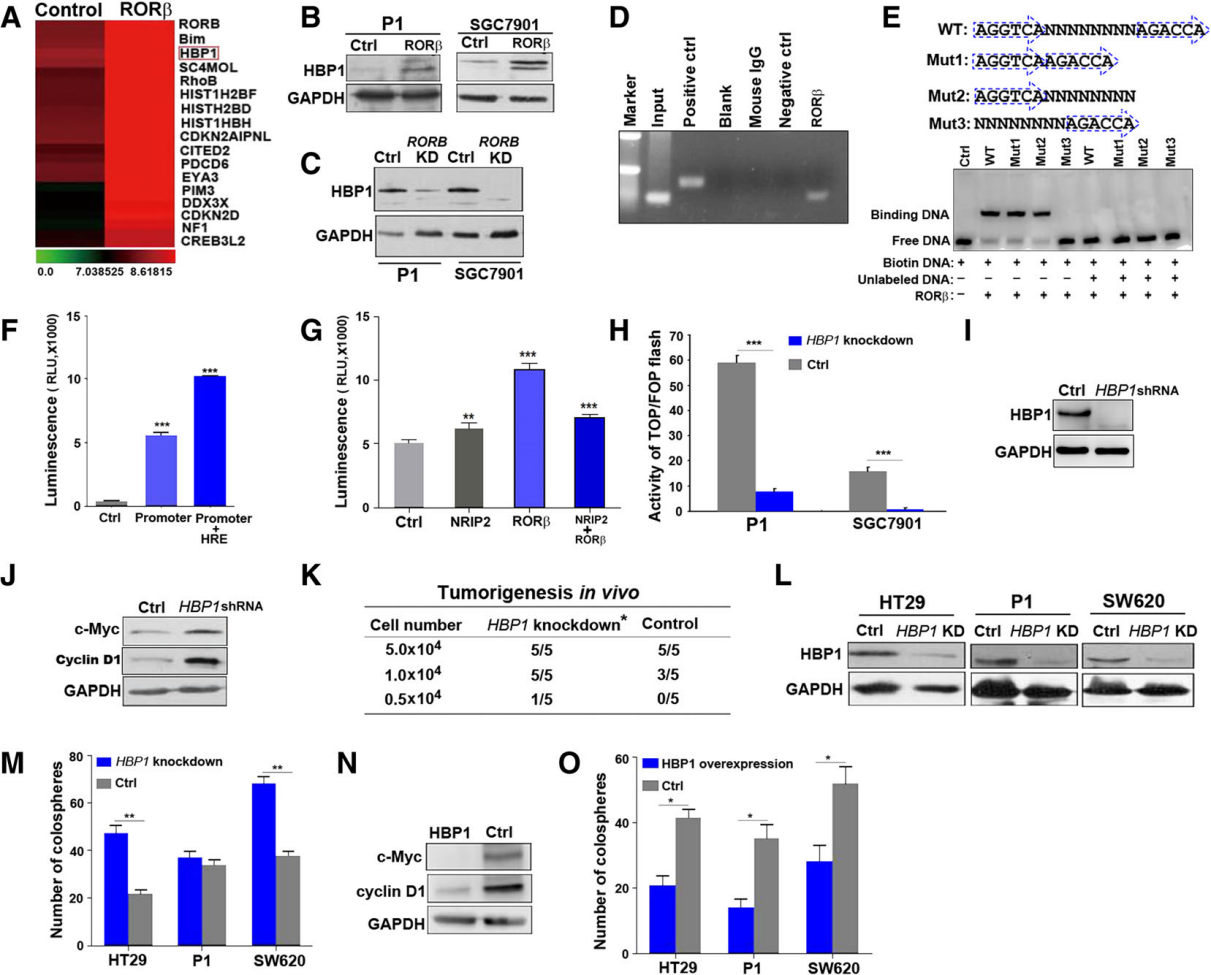
HBP1 is a crucial target of RORβ in regulation of the Wnt pathway. **a** Altered mRNA in RORβ-overexpressing cells. SGC7901 cells were transiently transfected with *RORB*/pReceiver and control pReceiver plasmids for 24 h. Total RNA was purified for global cDNA GeneChip scanning. The most significant up-regulated genes are listed. **b** Detection of HBP1 in RORβ-overexpressing cells. HBP1 was detected by western blotting in cells overexpressing RORβ. P1 cells infected with blank lentivirus and SGC7901 cells transfected with pReceiver plasmids were used as controls **c** Detection of HBP1 in *RORB-*knockdown cells. HBP1 was detected by western blotting in *RORB-*knockdown cells that were produced by infection with *RORB* shRNA lentivirus. HBP1 expression was significantly decreased after knockdown of *RORB.* P1 and SGC7901 cells infected with scrambled shRNA lentivirus were used as controls. **d** ChIP analysis of the interaction between RORβ and *HBP1* upstream DNA. DNA fragments were immunoprecipitated by anti-myc-tag antibodies agarose in RORβ-overexpressing SGC7901 cells after sonication. PCR was used for the detection of the *HBP1* upstream DNA sequence. The results showed that RORβ bound with the region upstream of *HBP1* DNA. Blank, normal mouse IgG was used as a negative control, and anti-RNA polymerase II was used as a positive control. **e** RORβ binds to hormone response elements (HRE) upstream of the *HBP1* promoter region. An EMSA assay was used to identify the seed region for RORβ binding within upstream hormone response elements of the *HBP1* promoter region. Three mutants containing different potential binding sequences were constructed. The results showed that the hormone response element sequence AGGTCA is essential for RORβ binding with the *HBP1* promoter region. **f** HRE increased the activity of the promoter. Plasmids containing HRE or the promoter of *HBP1* were co-transfected into 239 T cells for 24 h. Luciferase activity was evaluated by the dual-luciferase reporter assay system. The results showed that HRE increased the *HBP1* promoter activity, ***p < 0.001 (ANOVA). pRL3 plasmids were used as a control. **g** NRIP2 attenuated RORβ transactivation. The luciferase activity of the *HBP1* promoter was determined in 293 T cells at 24 h after co-transfection of *RORB* and/or *NRIP2* as well as in pRL3 plasmids containing HRE and the *HBP1* promoter. The results showed that NRIP2 attenuated RORβ transactivation, ***p < 0.001 (ANOVA). Blank pRL3 plasmids were used as a control. **h** NRIP2 could not activate Wnt activity in *HBP1*-silenced cells. Wnt activity was evaluated by a luciferase activity assay in *HBP1*-silenced cells and scrambled P1 and SGC7901 cells (control) 24 h after co-transfection with Top/Fop flash reporters and *NRIP2* plasmids. The results showed that NRIP2 could not activate Wnt activity in cells after silencing *HBP1*. ***p < 0.001 (ANOVA). **i** Detection of HBP1 in *HBP1-*knockdown cells. HBP1 was detected by western blotting in the SGC7901 cells with knockdown of *HBP1* by shRNAs. SGC7901 cells transfected with scrambled shRNAs as a control. **j** Wnt activity in *HBP1-*knockdown cells. c-Myc and cyclin D1 were detected by western blotting in the above *HBP1-*knockdown and scrambled SGC7901 cells. **k** Tumorigenicity of *HBP1-*knockdown cells. *HBP1-*knockdown SGC7901 cells and their control cells infected with blank lentivirus were injected into naked Balb/c mice, respectively. Tumor formation was quantified within 4 weeks. The results showed that silence of *HBP1* significantly increased tumorigenicity (*p* < 0.05, Multivariate logistic analysis). **l** HBP1 expression in *HBP1-*knockdown colorectal cancer cells. Colorectal cancer cells were infected with *HBP1* shRNA lentivirus for knockdown of HBP1. HBP1 was detected by western blotting in these *HBP1-*knockdown and scrambled colorectal cancer cells (control). **m** Quantification of colospheres in *HBP1-*knockdown cells. Colospheres were counted in *HBP1-*knockdown and scrambled cells. The number of colospheres was significantly increased in *HBP1*-knockdown cells. **p < 0.01 (ANOVA). **n** Detection of the Wnt downstream targets in HBP1-overexpressing cells. c-Myc and cyclin D1 were analyzed by western blotting in HBP1-overexpressing and control (transfected with pCMV-XL4 plasmids) P1 cells. **o** Quantification of colospheres in HBP1-overexpressing cells. The number of colospheres was counted in the above HBP1-overexpressing and control cells. The results showed that HBP1 significantly inhibited colosphere formation. *p < 0.05 (ANOVA)

To investigate how RORβ inhibits Wnt activation, we first analyzed changes in mRNA expression in cells overexpressing RORβ. Genechip-scanning experiments showed that *HBP1*, a protein that blocks TCF binding to DNA [33], was significantly increased in cells overexpressing RORβ (Fig. 6a). This result was subsequently confirmed by western blot analysis (Fig. 6b). However, HBP1 obviously reduced following *RORB* silencing (Fig. 6c). Similiarly, HBP1 was also reduced in the CCICs and crypts of intestinal mucosa from *Rorb* ^−/−^ mice (Additional file 1: Figures S6 and S7a). These results suggest that HBP1 is a downstream target of RORβ. Furthermore, Chromatin Immunoprecipitation (ChIP) experiments showed that RORβ could bind to *HBP1* upstream DNA sequences (Fig. 6d). Next, the upstream promoter region sequences of *HBP1* were analyzed, and several RORβ binding sequences were identified (Additional file 1: Figure S7b), EMSA detection confirmed that the HRE sequence AGGTCA is essential for RORβ binding to the *HBP1* promoter region (Fig. 6e). By co-transfecting a *RORB* plasmid and a reporter encoding luciferase under the control of the *HBP*1 promoter region sequences, we found that RORβ obviously enhanced downstream luciferase activity, while co-transfection with *NRIP2* significantly weakened its transcription activity (Fig. 6f and g). NRIP2 could not activate the Wnt activity in *HBP1*-silencing cells (Fig. 6h). Western blots revealed that the Wnt pathway was activated in *HBP*1 knockdown cells (Fig. 6i and j). These *HBP*1 knockdown cells were inoculated into NOD/SCID mice, and they exhibited significantly increased tumorigenicity (Fig. 6k). The in vitro colosphere-formation potential was also enhanced in these cells (Fig. 6l and m). However, both Wnt activation and the number of colospheres decreased in cells with reinforced *HBP1* expression (Fig. 6n and o). In summary, these data suggested that the interactions between NRIP2, RORβ, and HBP1 regulated Wnt pathway activation and the self-renewal of CCICs.

## Discussion

**Fig. 7:**
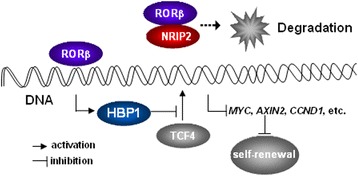
Illustration of interaction between NRIP2, RORβ, and HBP1

Using a retroviral library screening strategy, we demonstrated increased expression of NRIP2 in CCICs. NRIP2 was shown to be a novel interactor with the Wnt pathway. RORβ was identified as a key target of NRIP2, through which NRIP2 regulates the activity of the Wnt pathway. The NRIP2-RORβ interaction reduces *HBP1* transcription, thereby attenuating HBP1-dependent inhibition of the TCF4-DNA complex, finally promoting the self-renewal of CCICs by up-regulating Wnt pathways (Fig. 7).

NRIP2 belongs to the aspartic protease family [34]. Unlike other members of this family, such as Ddi1, both the ubiquitin-associated domain and the ubiquitin-like binding domain are absent from the NRIP2 sequence, suggesting that NRIP2 has a different function from the other aspartic protease family members [35]. NIX1, a murine NRIP2 homolog, can bind directly to the C-terminal ligand-binding domain (LBD) of mouse RORβ independently of retinoic acid and thyroid hormone T3 to inhibit the transcriptional activity of RORβ. NIX1 was found to participate in transcriptional repression in yeast cells [30], but the mechanism whereby NRIP2 regulates the Wnt activity and the self-renewal of cancer initiating cells has been rarely reported.

Previously, *RORB* was primarily detected by Northern blotting, the expression of which was restricted to the central nervous system, in particular, to regions involved in modulating circadian rhythms, such as the suprachiasmatic nucleus, the pineal gland, and the retina [31, 32]. Recently, RORβ was detected in tissues outside of the nervous system, such as normal bone tissue, the endometrium and pancreatic cancer [36–38]. *RORB*, which had a high expression level in the endometrium in healthy pre- or post-menopausal women, was significantly down-regulated in endometrial cancer cells [38]. We detected RORβ expression in normal intestinal epithelial cells and intestinal tumors; moreover, *RORB* was also decreased in cancer tissues, suggesting that the distribution of RORβ may be more widespread than is currently known and that RORβ may play a role as a tumor suppressor. Similar to RORα and RORγ, RORβ contains 4 functional domains, including an amino-terminal A/B domain, a DNA-binding domain (DBD), a hinge region, and a carboxy-terminal LBD [39, 40]. The DBD is highly homologous between RORβ and RORγ (92%), but the LBD is not well conserved among RORs [41]. RORβ includes RORβ1 and RORβ2 isoforms. RORβ1 and RORβ2 are characterized by different A/B domains that contain 2 and 13 amino acids, respectively. The N-terminal 2^nd^–13^th^ amino acids of RORβ1 are replaced by an arginine in RORβ2 [42]. The molecular function of RORβ needs to be further clarified. RORβ is considered to be a critical transcription factor regulating rod differentiation [43, 44]. RORβ1 induces the expression of the early key transcription factors Ptf1a and Foxn4 and promotes the differentiation of amacrine and horizontal cells [45]. RORβ also regulates bone formation by inhibiting Runx2 activity [36]. There is relatively little evidence supporting a functional relationship between the RORβ and Wnt activities related to the self-renewal of CCICs. It has been reported that RORα binds to the promoter region of *CTNNB1* to inhibit Wnt activity [46], which is involved in a non-canonical Wnt pathway. Among the target molecules of NRIP2, RORβ is homologous to RORα [42, 47], but whether RORβ affects the Wnt pathway remains unclear. Here, we show that RORβ suppresses the Wnt pathway and unlike RORα, RORβ neither binds with β-catenin nor affects its transcription.

RORβ is a DNA transcription enhancer. Thus, we screened for target genes at the transcriptional level, enabling the discovery of HBP1 as an interaction partner. RORβ enhances the transcription of *HBP1* by binding to its HRE region upstream promoter. HBP1 belongs to the sequence-specific, HMG family of transcription factors [48]. As a putative suppressor of the Wnt pathway, HBP1 may also inhibit the transcription of TCF4 targets by directly blocking the binding of TCF4 with DNA [33, 49]. Therefore, we speculate that RORβ may affect the activity of the Wnt pathway by regulating HBP1 transcription, and NRIP2 up-regulates Wnt activity by attenuating RORβ transcriptional activity. Due to the critical role of the Wnt pathway in CCIC self-renewal, HBP1 also participated in the regulation of CCIC self-renewal. These results indicate that the NRIP2/RORβ/HBP1 pathway is a beneficial supplement to the Wnt pathway. In addition to the activation of the Wnt pathway by NRIP2/RORβ/HBP1, NRIP2 is also associated with DNA mismatch repair in colorectal cancer cells, and RORβ may be correlated with tumorigenesis and tumor stages (Additional file 2), suggesting that the NRIP2/RORβ/HBP1 pathway is also involved in other biological processes.

## Conclusion

In this study, we identified NRIP2 as a novel molecule acting in Wnt pathway. The interaction between NRIP2 and RORβ activates downstream target HBP1 and is probably involved in CCIC self-renewal. For the positive role in Wnt, NRIP2 may be a potential alternative target for inhibiting CCIC self-renewal.

## Additional files

Additional file 1: Determination of *NRIP2* and *HBP1* in the colorectal cancer initiating cells and detection of *RORB* in the colorectal epithelials. (DOC 3098 kb)
Additional file 2: Analysis of the relationship between *NRIP2*, *RORB* and clinical parameters. (DOCX 1238 kb)

## Abbreviations

CCICs: Colorectal cancer initiating cells
ChIP: Chromatin immunoprecipitation
Co-IP: Co-immunoprecipitation
CSC: cancer stem cell
Dvl: Disheveled
EMSA: Electrophoretic mobility shift assay
ES: Embryonic stem cells
FACS: Fluorescence-activated cell sorting
FISH: Fluorescence in situ hybridization
GSEA: Gene set enrichment analysis
GSK-3β: Glycogen synthase kinase-3β
HBP1: HMG box-containing protein 1
HMG: High-mobility-group
HRE: Hormone response elemen
IHC: Immunohistochemical
iPS: Induced pluripotent cells
LEF: Lymphoid enhancer factor
LRP5/6: Low-density lipoprotein receptor-related protein 5/6
NRIP2: Nuclear receptor-interacting protein 2
ROR2/PTK7: Receptor tyrosine kinase-like orphan receptor 2/protein tyrosine kinase 7
RORβ: Retinoic acid-related orphan receptor β
SP: Side population
TCF: T-cell factor
