# Supplementary material for: Up-regulated NRIP2 in colorectal cancer initiating cells modulates the Wnt pathway by targeting RORβ
Source: Mol Cancer. 2017 Jan 31;16:20. doi: 10.1186/s12943-017-0590-2 (PMC5282884; doi:10.1186/s12943-017-0590-2)
Supplement: Additional file 1: — Determination of NRIP2 and HBP1 in the colorectal cancer initiating cells and detection of RORB in the colorectal epithelials. (DOC 3098 kb) [file 12943_2017_590_MOESM1_ESM.doc]

***Figure Supplements***

**Figure S2**

**
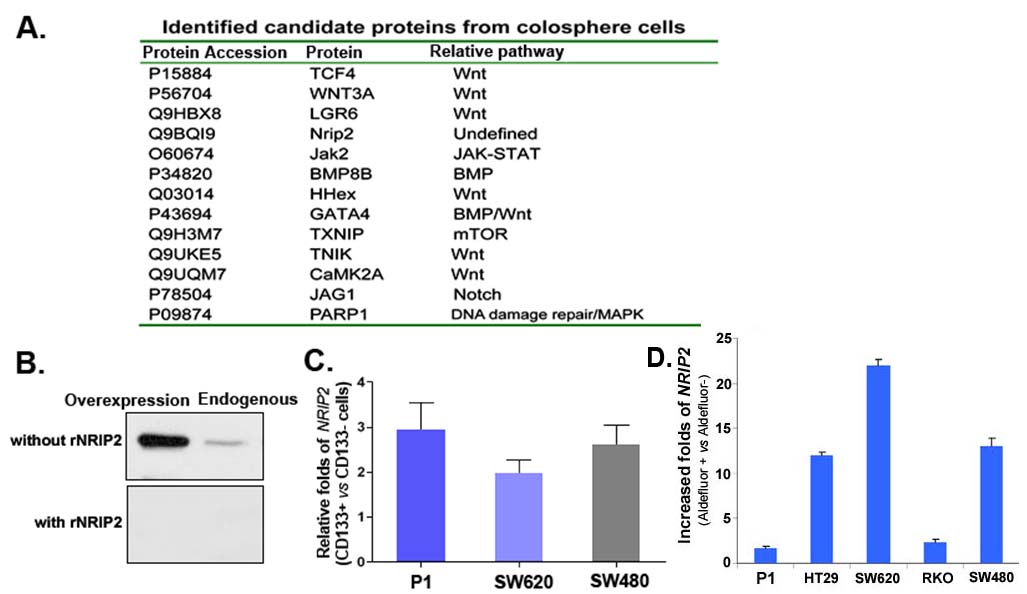
**

**Identified candidate proteins and determination of *NRIP2* in CD133+ cells**

1. **Identified candidate proteins from colosphere cells.** Candidate proteins were screened from a retroviral cDNA expression library constructed from SW620 colosphere cells by colosphere formation and Top/Fop flash reporter assays as well as DNA sequencing. **B**. **NRIP2 antibody control**. Antibodies to NRIP2 from three famous companies were testified for their reactive specificity. All antibodies were diluted as indication of product datasheet respectively, then add 100 μg/mL recombinant human NRIP2 protein (rNRIP2) from E.*coli* expression at 4°C overnight. Finally, the diluted antibody solutions were subject to WB analysis. The menbranes were incubated with the diluted antibody solution treated with or without recombinant rNRIP2 respectively. We found that antibody from Novus Co. gave a good performance. **C**. **Determination of *NRIP2* in CD133+colorectal cancer** **initiating cells**. The colorectal cancer cells were sorted by MACS coated anti- CD133 antibodies (Miltenyi Biotec, Germany). And then the level of *NRIP2* in CD133+ and CD133- cells was determined by Taqman RT-qPCR. The level of *NRIP2* in CD133+ colorectal cancer initiating cells was significantly high compared with CD133 - cells .(**p*<0.05; ANOVA). **D**. **Determination of *NRIP2* in Aldefluor+ colorectal cancer** **cells**. The colorectal cancer cells were sorted by FACS (Stem cells, USA) after incubation with ALDH enzyme substrate Aldefluor. And then the level of *NRIP2* in Aldefluor+ and Aldefluor- cells was determined by Taqman RT-qPCR. The level of *NRIP2* in Aldefluor+ colorectal cancer cells was significantly high compared with Aldefluor- cells .(**p*<0.05; ANOVA).

**Figure S3**

**
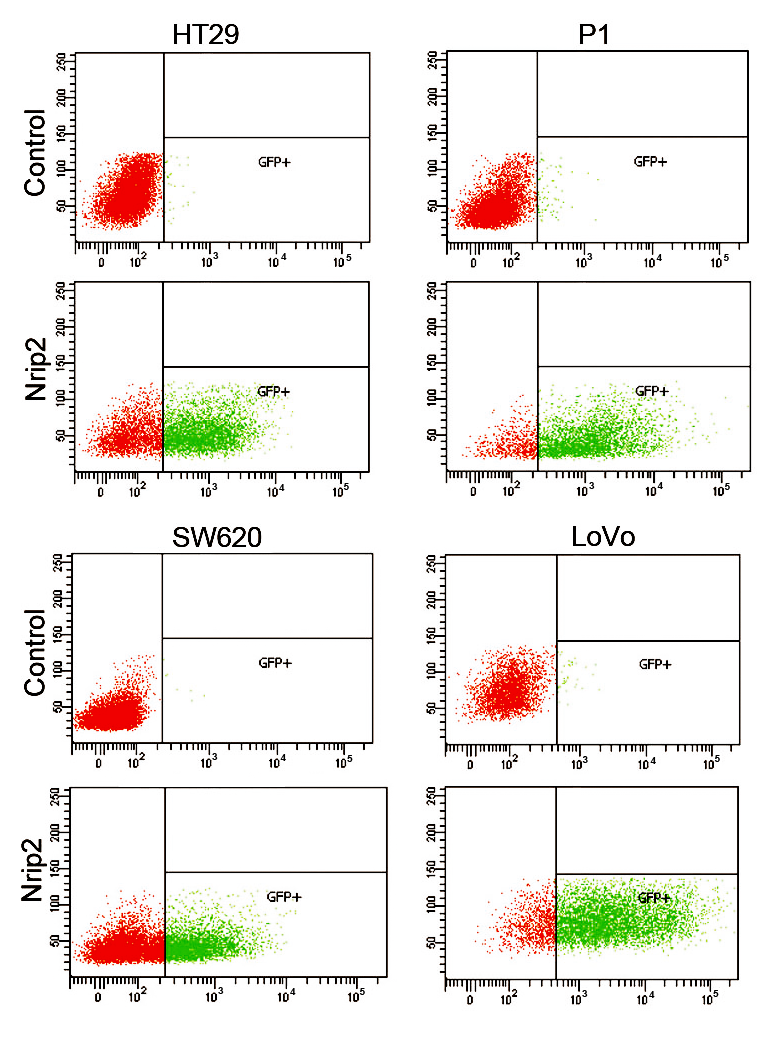
**

**FCM analyzed the infection efficiency in colorectal cancer cells**

To establish colorectal cancer cell lines that stably expressing NRIP2, HT29, P1 and SW620 cells were infected with recombinant lentiviral particles including *NRIP2* or *GFP* control, respectively. The infection efficiency was analyzed by FCM. The efficiency of HT29, P1, SW620 and LoVo was 63.5%, 85.0%, 40% and 84.4%, respectively.

**Figure S4**

**
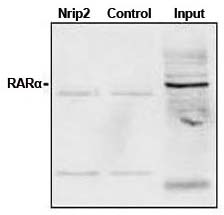
**

**NRIP2 unbound with RARα**.

Lysates from NRIP2-overexpressed P1 colorectal cancer cells and control cells were subjected to Co-IP, followed by WB with anti- RARα antibody. The results showed that NRIP2 did not interact with RARα.

**Figure S5**

**
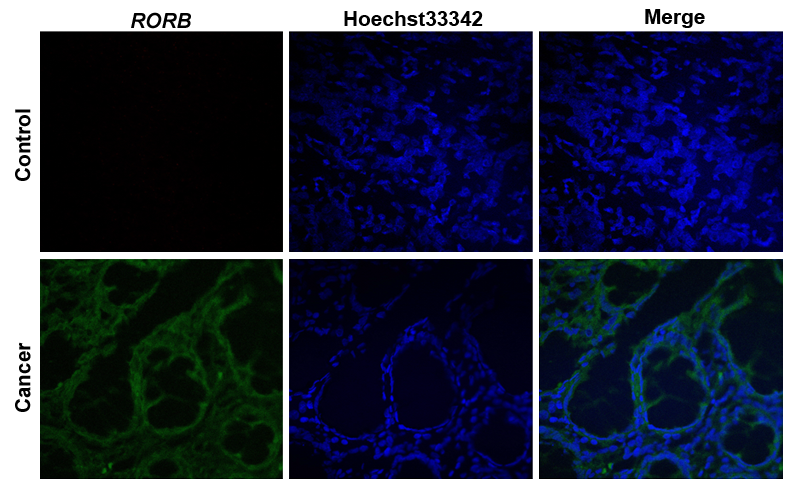
**

**The expression of *RORB* in the colorectal epithelials**

Slides containing primary normal colorectal tissue were hybridized with labeled probes for *RORB* mRNA respectively, with a nonsense probe as a negative control. FISH analysis showed *RORB* expression in primary colorectal epithelial cells.

**Figure S6**

**
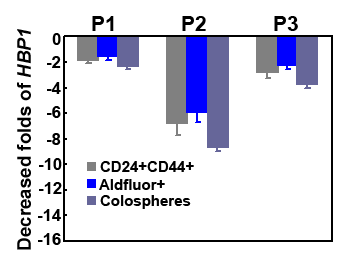
**

**Determination of *HBP1* in colorectal cancer initiating cells**

*HBP1* was determined by Taqman RT-qPCR. The results showed that level of *HBP1* in CD24+CD44+, Aldfluor+ and colosphere cells was significantly high compared with their matched cells .(**p*<0.05; ANOVA, respectively).

**Figure S7**

**
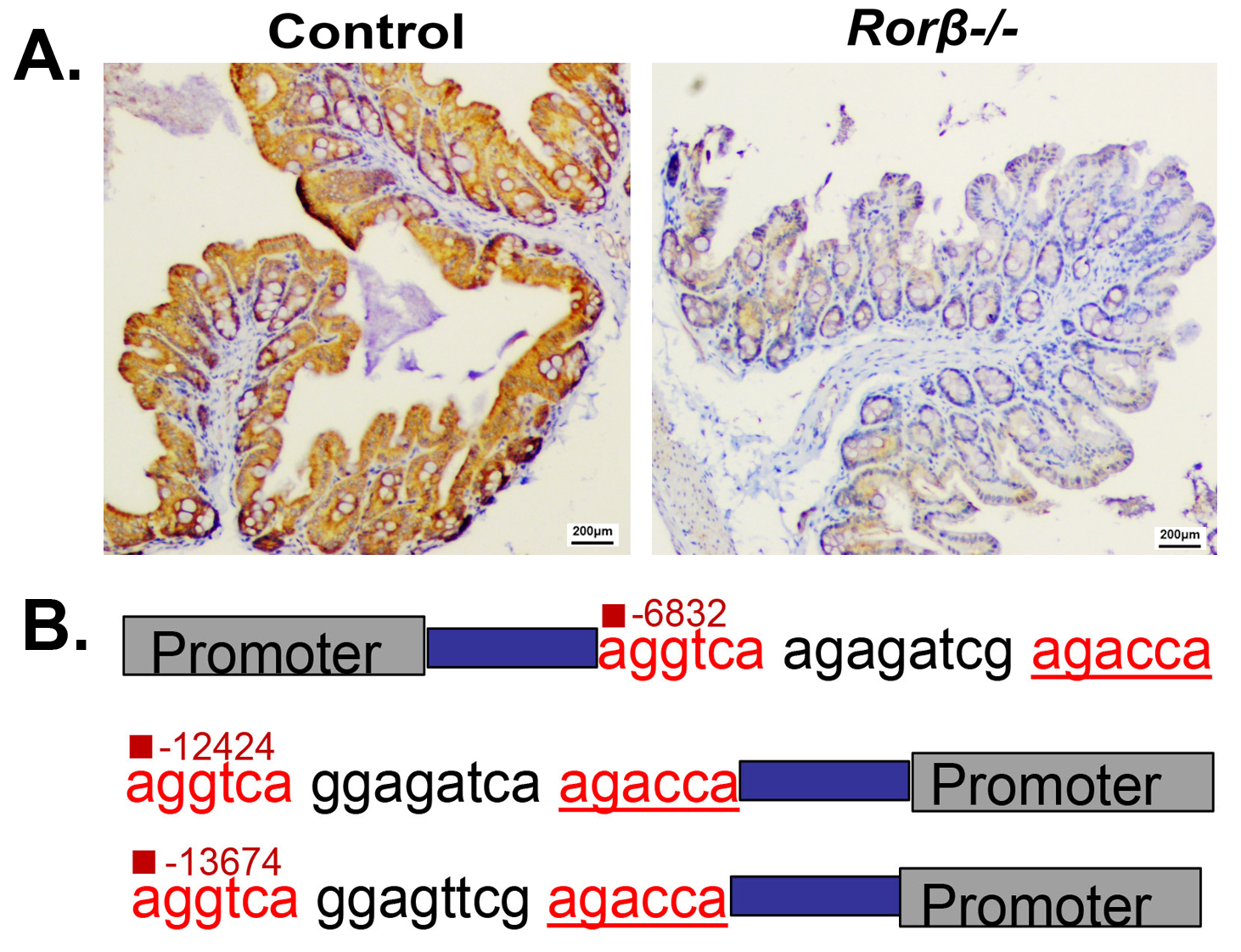
**

**Detection of HBP1 and analysis of upstream sequences of *HBP1***

**A**. **Detection of HBP1 in the intestinal mucosa.** Expression of HBP1 in the crypts of intestinal mucosa from *Rorb*-/- mice was detected by IHC staining. **B**. **Analysis of upstream sequences of** ***HBP1.*** Several similar sequences (AGGTCA-------AGACCA) were found around potential promoter regions of *HBP1*.
