# Supplementary material for: Up-regulated NRIP2 in colorectal cancer initiating cells modulates the Wnt pathway by targeting RORβ
Source: Mol Cancer. 2017 Jan 31;16:20. doi: 10.1186/s12943-017-0590-2 (PMC5282884; doi:10.1186/s12943-017-0590-2)
Supplement: Additional file 2: — Analysis of the relationship between NRIP2, RORB and clinical parameters. (DOCX 1238 kb) [file 12943_2017_590_MOESM2_ESM.docx]

**Fit Y by X Group**

**Oneway Analysis of NRIP2 By Age at DX 2**

Missing Rows

1

**Oneway Anova**

**Summary of Fit**

|  |  |
| --- | --- |
| Rsquare | 0.013628 |
| Adj Rsquare | 0.004805 |
| Root Mean Square Error | 0.305025 |
| Mean of Response | 4.652489 |
| Observations (or Sum Wgts) | 565 |

**Analysis of Variance**

| **Source** | **DF** | **Sum of Squares** | **Mean Square** | **F Ratio** | **Prob > F** |
| --- | --- | --- | --- | --- | --- |
| Age at DX 2 | 5 | 0.718584 | 0.143717 | 1.5447 | 0.1741 |
| Error | 559 | 52.009539 | 0.093040 |  |  |
| C. Total | 564 | 52.728123 |  |  |  |

**Means for Oneway Anova**

| **Level** | **Number** | **Mean** | **Std Error** | **Lower 95%** | **Upper 95%** |
| --- | --- | --- | --- | --- | --- |
| <40 | 17 | 4.54939 | 0.07398 | 4.4041 | 4.6947 |
| 40-49 | 49 | 4.64217 | 0.04358 | 4.5566 | 4.7278 |
| 50-59 | 84 | 4.71290 | 0.03328 | 4.6475 | 4.7783 |
| 60-69 | 151 | 4.67073 | 0.02482 | 4.6220 | 4.7195 |
| 70-79 | 182 | 4.64136 | 0.02261 | 4.5969 | 4.6858 |
| >=80 | 82 | 4.60925 | 0.03368 | 4.5431 | 4.6754 |

Std Error uses a pooled estimate of error variance

**Oneway Analysis of ROBb_43 By Age at DX 2**

Missing Rows

1

**Oneway Anova**

**Summary of Fit**

|  |  |
| --- | --- |
| Rsquare | 0.00158 |
| Adj Rsquare | -0.00735 |
| Root Mean Square Error | 0.168174 |
| Mean of Response | 3.062327 |
| Observations (or Sum Wgts) | 565 |

**Analysis of Variance**

| **Source** | **DF** | **Sum of Squares** | **Mean Square** | **F Ratio** | **Prob > F** |
| --- | --- | --- | --- | --- | --- |
| Age at DX 2 | 5 | 0.025026 | 0.005005 | 0.1770 | 0.9712 |
| Error | 559 | 15.809888 | 0.028282 |  |  |
| C. Total | 564 | 15.834914 |  |  |  |

**Means for Oneway Anova**

| **Level** | **Number** | **Mean** | **Std Error** | **Lower 95%** | **Upper 95%** |
| --- | --- | --- | --- | --- | --- |
| <40 | 17 | 3.03860 | 0.04079 | 2.9585 | 3.1187 |
| 40-49 | 49 | 3.05051 | 0.02402 | 3.0033 | 3.0977 |
| 50-59 | 84 | 3.06775 | 0.01835 | 3.0317 | 3.1038 |
| 60-69 | 151 | 3.06807 | 0.01369 | 3.0412 | 3.0950 |
| 70-79 | 182 | 3.05993 | 0.01247 | 3.0354 | 3.0844 |
| >=80 | 82 | 3.06348 | 0.01857 | 3.0270 | 3.1000 |

Std Error uses a pooled estimate of error variance

**Oneway Analysis of ROBb_85 By Age at DX 2**

Missing Rows

1

**Oneway Anova**

**Summary of Fit**

|  |  |
| --- | --- |
| Rsquare | 0.006245 |
| Adj Rsquare | -0.00264 |
| Root Mean Square Error | 0.147129 |
| Mean of Response | 2.710586 |
| Observations (or Sum Wgts) | 565 |

**Analysis of Variance**

| **Source** | **DF** | **Sum of Squares** | **Mean Square** | **F Ratio** | **Prob > F** |
| --- | --- | --- | --- | --- | --- |
| Age at DX 2 | 5 | 0.076042 | 0.015208 | 0.7026 | 0.6217 |
| Error | 559 | 12.100641 | 0.021647 |  |  |
| C. Total | 564 | 12.176683 |  |  |  |

**Means for Oneway Anova**

| **Level** | **Number** | **Mean** | **Std Error** | **Lower 95%** | **Upper 95%** |
| --- | --- | --- | --- | --- | --- |
| <40 | 17 | 2.67323 | 0.03568 | 2.6031 | 2.7433 |
| 40-49 | 49 | 2.71681 | 0.02102 | 2.6755 | 2.7581 |
| 50-59 | 84 | 2.70292 | 0.01605 | 2.6714 | 2.7345 |
| 60-69 | 151 | 2.70628 | 0.01197 | 2.6828 | 2.7298 |
| 70-79 | 182 | 2.72421 | 0.01091 | 2.7028 | 2.7456 |
| >=80 | 82 | 2.70016 | 0.01625 | 2.6682 | 2.7321 |

Std Error uses a pooled estimate of error variance

**Fit Y by X Group**

**Oneway Analysis of NRIP2 By Sex**

**Oneway Anova**

**Summary of Fit**

|  |  |
| --- | --- |
| Rsquare | 0.001088 |
| Adj Rsquare | -0.00068 |
| Root Mean Square Error | 0.306615 |
| Mean of Response | 4.653539 |
| Observations (or Sum Wgts) | 566 |

**t Test**

male-female

Assuming equal variances

|  |  |  |  |
| --- | --- | --- | --- |
| Difference | -0.02030 | t Ratio | -0.78388 |
| Std Err Dif | 0.02589 | DF | 564 |
| Upper CL Dif | 0.03056 | Prob > \|t\| | 0.4334 |
| Lower CL Dif | -0.07116 | Prob > t | 0.7833 |
| Confidence | 0.95 | Prob < t | 0.2167 |

**Analysis of Variance**

| **Source** | **DF** | **Sum of Squares** | **Mean Square** | **F Ratio** | **Prob > F** |
| --- | --- | --- | --- | --- | --- |
| Sex | 1 | 0.057768 | 0.057768 | 0.6145 | 0.4334 |
| Error | 564 | 53.023352 | 0.094013 |  |  |
| C. Total | 565 | 53.081120 |  |  |  |

**Means for Oneway Anova**

| **Level** | **Number** | **Mean** | **Std Error** | **Lower 95%** | **Upper 95%** |
| --- | --- | --- | --- | --- | --- |
| female | 256 | 4.66466 | 0.01916 | 4.6270 | 4.7023 |
| male | 310 | 4.64436 | 0.01741 | 4.6102 | 4.6786 |

Std Error uses a pooled estimate of error variance

**Oneway Analysis of ROBb_43 By Sex**

**Oneway Anova**

**Summary of Fit**

|  |  |
| --- | --- |
| Rsquare | 0.000771 |
| Adj Rsquare | -0.001 |
| Root Mean Square Error | 0.167618 |
| Mean of Response | 3.062056 |
| Observations (or Sum Wgts) | 566 |

**t Test**

male-female

Assuming equal variances

|  |  |  |  |
| --- | --- | --- | --- |
| Difference | -0.00934 | t Ratio | -0.65983 |
| Std Err Dif | 0.01416 | DF | 564 |
| Upper CL Dif | 0.01846 | Prob > \|t\| | 0.5096 |
| Lower CL Dif | -0.03714 | Prob > t | 0.7452 |
| Confidence | 0.95 | Prob < t | 0.2548 |

**Analysis of Variance**

| **Source** | **DF** | **Sum of Squares** | **Mean Square** | **F Ratio** | **Prob > F** |
| --- | --- | --- | --- | --- | --- |
| Sex | 1 | 0.012232 | 0.012232 | 0.4354 | 0.5096 |
| Error | 564 | 15.846035 | 0.028096 |  |  |
| C. Total | 565 | 15.858268 |  |  |  |

**Means for Oneway Anova**

| **Level** | **Number** | **Mean** | **Std Error** | **Lower 95%** | **Upper 95%** |
| --- | --- | --- | --- | --- | --- |
| female | 256 | 3.06717 | 0.01048 | 3.0466 | 3.0877 |
| male | 310 | 3.05783 | 0.00952 | 3.0391 | 3.0765 |

Std Error uses a pooled estimate of error variance

**Oneway Analysis of ROBb_85 By Sex**

**Oneway Anova**

**Summary of Fit**

|  |  |
| --- | --- |
| Rsquare | 2.754e-6 |
| Adj Rsquare | -0.00177 |
| Root Mean Square Error | 0.146935 |
| Mean of Response | 2.710584 |
| Observations (or Sum Wgts) | 566 |

**t Test**

male-female

Assuming equal variances

|  |  |  |  |
| --- | --- | --- | --- |
| Difference | 0.00049 | t Ratio | 0.039408 |
| Std Err Dif | 0.01241 | DF | 564 |
| Upper CL Dif | 0.02486 | Prob > \|t\| | 0.9686 |
| Lower CL Dif | -0.02388 | Prob > t | 0.4843 |
| Confidence | 0.95 | Prob < t | 0.5157 |

**Analysis of Variance**

| **Source** | **DF** | **Sum of Squares** | **Mean Square** | **F Ratio** | **Prob > F** |
| --- | --- | --- | --- | --- | --- |
| Sex | 1 | 0.000034 | 0.000034 | 0.0016 | 0.9686 |
| Error | 564 | 12.176651 | 0.021590 |  |  |
| C. Total | 565 | 12.176684 |  |  |  |

**Means for Oneway Anova**

| **Level** | **Number** | **Mean** | **Std Error** | **Lower 95%** | **Upper 95%** |
| --- | --- | --- | --- | --- | --- |
| female | 256 | 2.71032 | 0.00918 | 2.6923 | 2.7284 |
| male | 310 | 2.71081 | 0.00835 | 2.6944 | 2.7272 |

Std Error uses a pooled estimate of error variance

**Oneway Analysis of NRIP2 By cit-coloncancermolecularsubtype**

**Oneway Anova**

**Summary of Fit**

|  |  |
| --- | --- |
| Rsquare | 0.051054 |
| Adj Rsquare | 0.042581 |
| Root Mean Square Error | 0.299914 |
| Mean of Response | 4.653539 |
| Observations (or Sum Wgts) | 566 |

**Analysis of Variance**

| **Source** | **DF** | **Sum of Squares** | **Mean Square** | **F Ratio** | **Prob > F** |
| --- | --- | --- | --- | --- | --- |
| cit-coloncancermolecularsubtype | 5 | 2.710015 | 0.542003 | 6.0257 | <.0001* |
| Error | 560 | 50.371105 | 0.089948 |  |  |
| C. Total | 565 | 53.081120 |  |  |  |

**Means for Oneway Anova**

| **Level** | **Number** | **Mean** | **Std Error** | **Lower 95%** | **Upper 95%** |
| --- | --- | --- | --- | --- | --- |
| C1 | 116 | 4.65701 | 0.02785 | 4.6023 | 4.7117 |
| C2 | 104 | 4.56945 | 0.02941 | 4.5117 | 4.6272 |
| C3 | 75 | 4.57916 | 0.03463 | 4.5111 | 4.6472 |
| C4 | 59 | 4.73660 | 0.03905 | 4.6599 | 4.8133 |
| C5 | 152 | 4.65829 | 0.02433 | 4.6105 | 4.7061 |
| C6 | 60 | 4.79186 | 0.03872 | 4.7158 | 4.8679 |

Std Error uses a pooled estimate of error variance

**Means Comparisons**

**Comparisons for each pair using Student's t**

**Confidence Quantile**

| **t** | **Alpha** |
| --- | --- |
| 1.96421 | 0.05 |

**LSD Threshold Matrix**

| Abs(Dif)-LSD | **C6** | **C4** | **C5** | **C1** | **C3** | **C2** |
| --- | --- | --- | --- | --- | --- | --- |
| C6 | -0.10755 | -0.05275 | 0.04375 | 0.04117 | 0.11066 | 0.12690 |
| C4 | -0.05275 | -0.10846 | -0.01205 | -0.01461 | 0.05492 | 0.07113 |
| C5 | 0.04375 | -0.01205 | -0.06757 | -0.07135 | -0.00400 | 0.01387 |
| C1 | 0.04117 | -0.01461 | -0.07135 | -0.07735 | -0.00944 | 0.00801 |
| C3 | 0.11066 | 0.05492 | -0.00400 | -0.00944 | -0.09620 | -0.07953 |
| C2 | 0.12690 | 0.07113 | 0.01387 | 0.00801 | -0.07953 | -0.08169 |

Positive values show pairs of means that are significantly different.

**Connecting Letters Report**

| **Level** |  |  |  |  |  |  | **Mean** |
| --- | --- | --- | --- | --- | --- | --- | --- |
| C6 | A |  |  |  |  |  | 4.7918567 |
| C4 | A | B |  |  |  |  | 4.7365952 |
| C5 |  | B | C |  |  |  | 4.6582890 |
| C1 |  | B | C |  |  |  | 4.6570073 |
| C3 |  |  | C | D |  |  | 4.5791620 |
| C2 |  |  |  | D |  |  | 4.5694497 |

Levels not connected by same letter are significantly different.

**Ordered Differences Report**

| **Level** | **- Level** | **Difference** | **Std Err Dif** | **Lower CL** | **Upper CL** | **p-Value** |  |
| --- | --- | --- | --- | --- | --- | --- | --- |
| C6 | C2 | 0.2224070 | 0.0486213 | 0.126905 | 0.3179094 | <.0001* |  |
| C6 | C3 | 0.2126947 | 0.0519466 | 0.110661 | 0.3147287 | <.0001* |  |
| C4 | C2 | 0.1671456 | 0.0488819 | 0.071131 | 0.2631598 | 0.0007* |  |
| C4 | C3 | 0.1574332 | 0.0521906 | 0.054920 | 0.2599465 | 0.0027* |  |
| C6 | C1 | 0.1348494 | 0.0476923 | 0.041172 | 0.2285271 | 0.0049* |  |
| C6 | C5 | 0.1335677 | 0.0457264 | 0.043751 | 0.2233840 | 0.0036* |  |
| C5 | C2 | 0.0888393 | 0.0381661 | 0.013873 | 0.1638056 | 0.0203* |  |
| C1 | C2 | 0.0875577 | 0.0405007 | 0.008006 | 0.1671095 | 0.0311* |  |
| C4 | C1 | 0.0795879 | 0.0479580 | -0.014612 | 0.1737874 | 0.0976 |  |
| C5 | C3 | 0.0791270 | 0.0423211 | -0.004001 | 0.1622545 | 0.0620 |  |
| C4 | C5 | 0.0783062 | 0.0460034 | -0.012054 | 0.1686666 | 0.0893 |  |
| C1 | C3 | 0.0778453 | 0.0444379 | -0.009440 | 0.1651307 | 0.0804 |  |
| C6 | C4 | 0.0552615 | 0.0549881 | -0.052747 | 0.1632696 | 0.3153 |  |
| C3 | C2 | 0.0097123 | 0.0454335 | -0.079529 | 0.0989532 | 0.8308 |  |
| C5 | C1 | 0.0012817 | 0.0369754 | -0.071346 | 0.0739092 | 0.9724 |  |

**Oneway Analysis of ROBb_43 By cit-coloncancermolecularsubtype**

**Oneway Anova**

**Summary of Fit**

|  |  |
| --- | --- |
| Rsquare | 0.015617 |
| Adj Rsquare | 0.006828 |
| Root Mean Square Error | 0.166961 |
| Mean of Response | 3.062056 |
| Observations (or Sum Wgts) | 566 |

**Analysis of Variance**

| **Source** | **DF** | **Sum of Squares** | **Mean Square** | **F Ratio** | **Prob > F** |
| --- | --- | --- | --- | --- | --- |
| cit-coloncancermolecularsubtype | 5 | 0.247657 | 0.049531 | 1.7768 | 0.1157 |
| Error | 560 | 15.610611 | 0.027876 |  |  |
| C. Total | 565 | 15.858268 |  |  |  |

**Means for Oneway Anova**

| **Level** | **Number** | **Mean** | **Std Error** | **Lower 95%** | **Upper 95%** |
| --- | --- | --- | --- | --- | --- |
| C1 | 116 | 3.09551 | 0.01550 | 3.0651 | 3.1260 |
| C2 | 104 | 3.07159 | 0.01637 | 3.0394 | 3.1037 |
| C3 | 75 | 3.06595 | 0.01928 | 3.0281 | 3.1038 |
| C4 | 59 | 3.04661 | 0.02174 | 3.0039 | 3.0893 |
| C5 | 152 | 3.04583 | 0.01354 | 3.0192 | 3.0724 |
| C6 | 60 | 3.03229 | 0.02155 | 2.9899 | 3.0746 |

Std Error uses a pooled estimate of error variance

**Oneway Analysis of ROBb_85 By cit-coloncancermolecularsubtype**

**Oneway Anova**

**Summary of Fit**

|  |  |
| --- | --- |
| Rsquare | 0.009918 |
| Adj Rsquare | 0.001078 |
| Root Mean Square Error | 0.146726 |
| Mean of Response | 2.710584 |
| Observations (or Sum Wgts) | 566 |

**Analysis of Variance**

| **Source** | **DF** | **Sum of Squares** | **Mean Square** | **F Ratio** | **Prob > F** |
| --- | --- | --- | --- | --- | --- |
| cit-coloncancermolecularsubtype | 5 | 0.120763 | 0.024153 | 1.1219 | 0.3475 |
| Error | 560 | 12.055921 | 0.021528 |  |  |
| C. Total | 565 | 12.176684 |  |  |  |

**Means for Oneway Anova**

| **Level** | **Number** | **Mean** | **Std Error** | **Lower 95%** | **Upper 95%** |
| --- | --- | --- | --- | --- | --- |
| C1 | 116 | 2.69597 | 0.01362 | 2.6692 | 2.7227 |
| C2 | 104 | 2.72700 | 0.01439 | 2.6987 | 2.7553 |
| C3 | 75 | 2.73241 | 0.01694 | 2.6991 | 2.7657 |
| C4 | 59 | 2.72042 | 0.01910 | 2.6829 | 2.7579 |
| C5 | 152 | 2.69767 | 0.01190 | 2.6743 | 2.7211 |
| C6 | 60 | 2.70613 | 0.01894 | 2.6689 | 2.7433 |

Std Error uses a pooled estimate of error variance

**Oneway Analysis of NRIP2 By MMR.status**

Missing Rows

47

**Oneway Anova**

**Summary of Fit**

|  |  |
| --- | --- |
| Rsquare | 0.020562 |
| Adj Rsquare | 0.018667 |
| Root Mean Square Error | 0.30703 |
| Mean of Response | 4.651734 |
| Observations (or Sum Wgts) | 519 |

**t Test**

pMMR-dMMR

Assuming equal variances

|  |  |  |  |
| --- | --- | --- | --- |
| Difference | 0.126278 | t Ratio | 3.294477 |
| Std Err Dif | 0.038330 | DF | 517 |
| Upper CL Dif | 0.201580 | Prob > \|t\| | 0.0011* |
| Lower CL Dif | 0.050976 | Prob > t | 0.0005* |
| Confidence | 0.95 | Prob < t | 0.9995 |

**Analysis of Variance**

| **Source** | **DF** | **Sum of Squares** | **Mean Square** | **F Ratio** | **Prob > F** |
| --- | --- | --- | --- | --- | --- |
| MMR.status | 1 | 1.023136 | 1.02314 | 10.8536 | 0.0011* |
| Error | 517 | 48.736109 | 0.09427 |  |  |
| C. Total | 518 | 49.759245 |  |  |  |

**Means for Oneway Anova**

| **Level** | **Number** | **Mean** | **Std Error** | **Lower 95%** | **Upper 95%** |
| --- | --- | --- | --- | --- | --- |
| dMMR | 75 | 4.54370 | 0.03545 | 4.4741 | 4.6134 |
| pMMR | 444 | 4.66998 | 0.01457 | 4.6414 | 4.6986 |

Std Error uses a pooled estimate of error variance

**Oneway Analysis of ROBb_43 By MMR.status**

Missing Rows

47

**Oneway Anova**

**Summary of Fit**

|  |  |
| --- | --- |
| Rsquare | 0.001443 |
| Adj Rsquare | -0.00049 |
| Root Mean Square Error | 0.168393 |
| Mean of Response | 3.057316 |
| Observations (or Sum Wgts) | 519 |

**t Test**

pMMR-dMMR

Assuming equal variances

|  |  |  |  |
| --- | --- | --- | --- |
| Difference | -0.01817 | t Ratio | -0.86447 |
| Std Err Dif | 0.02102 | DF | 517 |
| Upper CL Dif | 0.02313 | Prob > \|t\| | 0.3877 |
| Lower CL Dif | -0.05947 | Prob > t | 0.8061 |
| Confidence | 0.95 | Prob < t | 0.1939 |

**Analysis of Variance**

| **Source** | **DF** | **Sum of Squares** | **Mean Square** | **F Ratio** | **Prob > F** |
| --- | --- | --- | --- | --- | --- |
| MMR.status | 1 | 0.021191 | 0.021191 | 0.7473 | 0.3877 |
| Error | 517 | 14.660103 | 0.028356 |  |  |
| C. Total | 518 | 14.681294 |  |  |  |

**Means for Oneway Anova**

| **Level** | **Number** | **Mean** | **Std Error** | **Lower 95%** | **Upper 95%** |
| --- | --- | --- | --- | --- | --- |
| dMMR | 75 | 3.07286 | 0.01944 | 3.0347 | 3.1111 |
| pMMR | 444 | 3.05469 | 0.00799 | 3.0390 | 3.0704 |

Std Error uses a pooled estimate of error variance

**Oneway Analysis of ROBb_85 By MMR.status**

Missing Rows

47

**Oneway Anova**

**Summary of Fit**

|  |  |
| --- | --- |
| Rsquare | 0.003338 |
| Adj Rsquare | 0.00141 |
| Root Mean Square Error | 0.148315 |
| Mean of Response | 2.708753 |
| Observations (or Sum Wgts) | 519 |

**t Test**

pMMR-dMMR

Assuming equal variances

|  |  |  |  |
| --- | --- | --- | --- |
| Difference | -0.02436 | t Ratio | -1.31588 |
| Std Err Dif | 0.01852 | DF | 517 |
| Upper CL Dif | 0.01201 | Prob > \|t\| | 0.1888 |
| Lower CL Dif | -0.06074 | Prob > t | 0.9056 |
| Confidence | 0.95 | Prob < t | 0.0944 |

**Analysis of Variance**

| **Source** | **DF** | **Sum of Squares** | **Mean Square** | **F Ratio** | **Prob > F** |
| --- | --- | --- | --- | --- | --- |
| MMR.status | 1 | 0.038089 | 0.038089 | 1.7315 | 0.1888 |
| Error | 517 | 11.372588 | 0.021997 |  |  |
| C. Total | 518 | 11.410677 |  |  |  |

**Means for Oneway Anova**

| **Level** | **Number** | **Mean** | **Std Error** | **Lower 95%** | **Upper 95%** |
| --- | --- | --- | --- | --- | --- |
| dMMR | 75 | 2.72960 | 0.01713 | 2.6960 | 2.7632 |
| pMMR | 444 | 2.70523 | 0.00704 | 2.6914 | 2.7191 |

Std Error uses a pooled estimate of error variance

**Oneway Analysis of NRIP2 By location**

**Oneway Anova**

**Summary of Fit**

|  |  |
| --- | --- |
| Rsquare | 0.001097 |
| Adj Rsquare | -0.00067 |
| Root Mean Square Error | 0.306614 |
| Mean of Response | 4.653539 |
| Observations (or Sum Wgts) | 566 |

**t Test**

proximal-distal

Assuming equal variances

|  |  |  |  |
| --- | --- | --- | --- |
| Difference | -0.02075 | t Ratio | -0.78716 |
| Std Err Dif | 0.02636 | DF | 564 |
| Upper CL Dif | 0.03102 | Prob > \|t\| | 0.4315 |
| Lower CL Dif | -0.07251 | Prob > t | 0.7842 |
| Confidence | 0.95 | Prob < t | 0.2158 |

**Analysis of Variance**

| **Source** | **DF** | **Sum of Squares** | **Mean Square** | **F Ratio** | **Prob > F** |
| --- | --- | --- | --- | --- | --- |
| location | 1 | 0.058252 | 0.058252 | 0.6196 | 0.4315 |
| Error | 564 | 53.022868 | 0.094012 |  |  |
| C. Total | 565 | 53.081120 |  |  |  |

**Means for Oneway Anova**

| **Level** | **Number** | **Mean** | **Std Error** | **Lower 95%** | **Upper 95%** |
| --- | --- | --- | --- | --- | --- |
| distal | 342 | 4.66175 | 0.01658 | 4.6292 | 4.6943 |
| proximal | 224 | 4.64100 | 0.02049 | 4.6008 | 4.6812 |

Std Error uses a pooled estimate of error variance

**Oneway Analysis of ROBb_43 By location**

**Oneway Anova**

**Summary of Fit**

|  |  |
| --- | --- |
| Rsquare | 0.000149 |
| Adj Rsquare | -0.00162 |
| Root Mean Square Error | 0.16767 |
| Mean of Response | 3.062056 |
| Observations (or Sum Wgts) | 566 |

**t Test**

proximal-distal

Assuming equal variances

|  |  |  |  |
| --- | --- | --- | --- |
| Difference | -0.00418 | t Ratio | -0.29 |
| Std Err Dif | 0.01441 | DF | 564 |
| Upper CL Dif | 0.02413 | Prob > \|t\| | 0.7719 |
| Lower CL Dif | -0.03249 | Prob > t | 0.6140 |
| Confidence | 0.95 | Prob < t | 0.3860 |

**Analysis of Variance**

| **Source** | **DF** | **Sum of Squares** | **Mean Square** | **F Ratio** | **Prob > F** |
| --- | --- | --- | --- | --- | --- |
| location | 1 | 0.002364 | 0.002364 | 0.0841 | 0.7719 |
| Error | 564 | 15.855903 | 0.028113 |  |  |
| C. Total | 565 | 15.858268 |  |  |  |

**Means for Oneway Anova**

| **Level** | **Number** | **Mean** | **Std Error** | **Lower 95%** | **Upper 95%** |
| --- | --- | --- | --- | --- | --- |
| distal | 342 | 3.06371 | 0.00907 | 3.0459 | 3.0815 |
| proximal | 224 | 3.05953 | 0.01120 | 3.0375 | 3.0815 |

Std Error uses a pooled estimate of error variance

**Oneway Analysis of ROBb_85 By location**

**Oneway Anova**

**Summary of Fit**

|  |  |
| --- | --- |
| Rsquare | 0.001593 |
| Adj Rsquare | -0.00018 |
| Root Mean Square Error | 0.146818 |
| Mean of Response | 2.710584 |
| Observations (or Sum Wgts) | 566 |

**t Test**

proximal-distal

Assuming equal variances

|  |  |  |  |
| --- | --- | --- | --- |
| Difference | 0.01197 | t Ratio | 0.948569 |
| Std Err Dif | 0.01262 | DF | 564 |
| Upper CL Dif | 0.03676 | Prob > \|t\| | 0.3432 |
| Lower CL Dif | -0.01282 | Prob > t | 0.1716 |
| Confidence | 0.95 | Prob < t | 0.8284 |

**Analysis of Variance**

| **Source** | **DF** | **Sum of Squares** | **Mean Square** | **F Ratio** | **Prob > F** |
| --- | --- | --- | --- | --- | --- |
| location | 1 | 0.019395 | 0.019395 | 0.8998 | 0.3432 |
| Error | 564 | 12.157289 | 0.021555 |  |  |
| C. Total | 565 | 12.176684 |  |  |  |

**Means for Oneway Anova**

| **Level** | **Number** | **Mean** | **Std Error** | **Lower 95%** | **Upper 95%** |
| --- | --- | --- | --- | --- | --- |
| distal | 342 | 2.70585 | 0.00794 | 2.6903 | 2.7214 |
| proximal | 224 | 2.71782 | 0.00981 | 2.6985 | 2.7371 |

Std Error uses a pooled estimate of error variance

**Oneway Analysis of NRIP2 By tnm.stage**

**Oneway Anova**

**Summary of Fit**

|  |  |
| --- | --- |
| Rsquare | 0.009968 |
| Adj Rsquare | 0.002909 |
| Root Mean Square Error | 0.306065 |
| Mean of Response | 4.653539 |
| Observations (or Sum Wgts) | 566 |

**Analysis of Variance**

| **Source** | **DF** | **Sum of Squares** | **Mean Square** | **F Ratio** | **Prob > F** |
| --- | --- | --- | --- | --- | --- |
| tnm.stage | 4 | 0.529120 | 0.132280 | 1.4121 | 0.2285 |
| Error | 561 | 52.551999 | 0.093676 |  |  |
| C. Total | 565 | 53.081120 |  |  |  |

**Means for Oneway Anova**

| **Level** | **Number** | **Mean** | **Std Error** | **Lower 95%** | **Upper 95%** |
| --- | --- | --- | --- | --- | --- |
| 0 | 4 | 4.59947 | 0.15303 | 4.2989 | 4.9001 |
| 1 | 33 | 4.56422 | 0.05328 | 4.4596 | 4.6689 |
| 2 | 264 | 4.65294 | 0.01884 | 4.6159 | 4.6899 |
| 3 | 205 | 4.68140 | 0.02138 | 4.6394 | 4.7234 |
| 4 | 60 | 4.61375 | 0.03951 | 4.5361 | 4.6914 |

Std Error uses a pooled estimate of error variance

**Means Comparisons**

**Comparisons for each pair using Student's t**

**Confidence Quantile**

| **t** | **Alpha** |
| --- | --- |
| 1.96420 | 0.05 |

**LSD Threshold Matrix**

| Abs(Dif)-LSD | **3** | **2** | **4** | **0** | **1** |
| --- | --- | --- | --- | --- | --- |
| 3 | -0.05938 | -0.02750 | -0.02060 | -0.22158 | 0.00441 |
| 2 | -0.02750 | -0.05233 | -0.04679 | -0.24939 | -0.02228 |
| 4 | -0.02060 | -0.04679 | -0.10976 | -0.29616 | -0.08076 |
| 0 | -0.22158 | -0.24939 | -0.29616 | -0.42509 | -0.28304 |
| 1 | 0.00441 | -0.02228 | -0.08076 | -0.28304 | -0.14800 |

Positive values show pairs of means that are significantly different.

**Connecting Letters Report**

| **Level** |  |  |  |  |  |  | **Mean** |
| --- | --- | --- | --- | --- | --- | --- | --- |
| 3 | A |  |  |  |  |  | 4.6813953 |
| 2 | A | B |  |  |  |  | 4.6529356 |
| 4 | A | B |  |  |  |  | 4.6137501 |
| 0 | A | B |  |  |  |  | 4.5994678 |
| 1 |  | B |  |  |  |  | 4.5642208 |

Levels not connected by same letter are significantly different.

**Ordered Differences Report**

| **Level** | **- Level** | **Difference** | **Std Err Dif** | **Lower CL** | **Upper CL** | **p-Value** |  |
| --- | --- | --- | --- | --- | --- | --- | --- |
| 3 | 1 | 0.1171745 | 0.0574074 | 0.004415 | 0.2299342 | 0.0417* |  |
| 2 | 1 | 0.0887148 | 0.0565109 | -0.022284 | 0.1997136 | 0.1170 |  |
| 3 | 0 | 0.0819275 | 0.1545181 | -0.221577 | 0.3854322 | 0.5962 |  |
| 3 | 4 | 0.0676452 | 0.0449245 | -0.020596 | 0.1558861 | 0.1327 |  |
| 2 | 0 | 0.0534678 | 0.1541873 | -0.249387 | 0.3563228 | 0.7289 |  |
| 4 | 1 | 0.0495293 | 0.0663318 | -0.080760 | 0.1798184 | 0.4556 |  |
| 2 | 4 | 0.0391855 | 0.0437732 | -0.046794 | 0.1251649 | 0.3711 |  |
| 0 | 1 | 0.0352470 | 0.1620418 | -0.283036 | 0.3535298 | 0.8279 |  |
| 3 | 2 | 0.0284597 | 0.0284919 | -0.027504 | 0.0844235 | 0.3183 |  |
| 4 | 0 | 0.0142823 | 0.1580511 | -0.296162 | 0.3247265 | 0.9280 |  |

**Oneway Analysis of ROBb_43 By tnm.stage**

**Oneway Anova**

**Summary of Fit**

|  |  |
| --- | --- |
| Rsquare | 0.01803 |
| Adj Rsquare | 0.011028 |
| Root Mean Square Error | 0.166608 |
| Mean of Response | 3.062056 |
| Observations (or Sum Wgts) | 566 |

**Analysis of Variance**

| **Source** | **DF** | **Sum of Squares** | **Mean Square** | **F Ratio** | **Prob > F** |
| --- | --- | --- | --- | --- | --- |
| tnm.stage | 4 | 0.285925 | 0.071481 | 2.5751 | 0.0368* |
| Error | 561 | 15.572343 | 0.027758 |  |  |
| C. Total | 565 | 15.858268 |  |  |  |

**Means for Oneway Anova**

| **Level** | **Number** | **Mean** | **Std Error** | **Lower 95%** | **Upper 95%** |
| --- | --- | --- | --- | --- | --- |
| 0 | 4 | 3.03842 | 0.08330 | 2.8748 | 3.2020 |
| 1 | 33 | 3.04161 | 0.02900 | 2.9846 | 3.0986 |
| 2 | 264 | 3.08574 | 0.01025 | 3.0656 | 3.1059 |
| 3 | 205 | 3.03833 | 0.01164 | 3.0155 | 3.0612 |
| 4 | 60 | 3.05173 | 0.02151 | 3.0095 | 3.0940 |

Std Error uses a pooled estimate of error variance

**Means Comparisons**

**Comparisons for each pair using Student's t**

**Confidence Quantile**

| **t** | **Alpha** |
| --- | --- |
| 1.96420 | 0.05 |

**LSD Threshold Matrix**

| Abs(Dif)-LSD | **2** | **4** | **1** | **0** | **3** |
| --- | --- | --- | --- | --- | --- |
| 2 | -0.02848 | -0.01279 | -0.01629 | -0.11753 | 0.01695 |
| 4 | -0.01279 | -0.05975 | -0.06080 | -0.15568 | -0.03463 |
| 1 | -0.01629 | -0.06080 | -0.08056 | -0.17007 | -0.05811 |
| 0 | -0.11753 | -0.15568 | -0.17007 | -0.23140 | -0.16513 |
| 3 | 0.01695 | -0.03463 | -0.05811 | -0.16513 | -0.03232 |

Positive values show pairs of means that are significantly different.

**Connecting Letters Report**

| **Level** |  |  |  |  |  |  | **Mean** |
| --- | --- | --- | --- | --- | --- | --- | --- |
| 2 | A |  |  |  |  |  | 3.0857411 |
| 4 | A | B |  |  |  |  | 3.0517308 |
| 1 | A | B |  |  |  |  | 3.0416062 |
| 0 | A | B |  |  |  |  | 3.0384153 |
| 3 |  | B |  |  |  |  | 3.0383306 |

Levels not connected by same letter are significantly different.

**Ordered Differences Report**

| **Level** | **- Level** | **Difference** | **Std Err Dif** | **Lower CL** | **Upper CL** | **p-Value** |  |
| --- | --- | --- | --- | --- | --- | --- | --- |
| 2 | 3 | 0.0474104 | 0.0155097 | 0.016946 | 0.0778746 | 0.0023* |  |
| 2 | 0 | 0.0473258 | 0.0839327 | -0.117535 | 0.2121864 | 0.5731 |  |
| 2 | 1 | 0.0441349 | 0.0307620 | -0.016288 | 0.1045577 | 0.1519 |  |
| 2 | 4 | 0.0340103 | 0.0238282 | -0.012793 | 0.0808136 | 0.1540 |  |
| 4 | 3 | 0.0134001 | 0.0244549 | -0.034634 | 0.0614345 | 0.5839 |  |
| 4 | 0 | 0.0133155 | 0.0860359 | -0.155676 | 0.1823074 | 0.8771 |  |
| 4 | 1 | 0.0101246 | 0.0361081 | -0.060799 | 0.0810482 | 0.7793 |  |
| 1 | 3 | 0.0032755 | 0.0312500 | -0.058106 | 0.0646568 | 0.9166 |  |
| 1 | 0 | 0.0031909 | 0.0882083 | -0.170068 | 0.1764498 | 0.9712 |  |
| 0 | 3 | 0.0000847 | 0.0841127 | -0.165130 | 0.1652990 | 0.9992 |  |

**Oneway Analysis of ROBb_85 By tnm.stage**

**Oneway Anova**

**Summary of Fit**

|  |  |
| --- | --- |
| Rsquare | 0.008031 |
| Adj Rsquare | 0.000958 |
| Root Mean Square Error | 0.146734 |
| Mean of Response | 2.710584 |
| Observations (or Sum Wgts) | 566 |

**Analysis of Variance**

| **Source** | **DF** | **Sum of Squares** | **Mean Square** | **F Ratio** | **Prob > F** |
| --- | --- | --- | --- | --- | --- |
| tnm.stage | 4 | 0.097794 | 0.024448 | 1.1355 | 0.3388 |
| Error | 561 | 12.078891 | 0.021531 |  |  |
| C. Total | 565 | 12.176684 |  |  |  |

**Means for Oneway Anova**

| **Level** | **Number** | **Mean** | **Std Error** | **Lower 95%** | **Upper 95%** |
| --- | --- | --- | --- | --- | --- |
| 0 | 4 | 2.68343 | 0.07337 | 2.5393 | 2.8275 |
| 1 | 33 | 2.69750 | 0.02554 | 2.6473 | 2.7477 |
| 2 | 264 | 2.72457 | 0.00903 | 2.7068 | 2.7423 |
| 3 | 205 | 2.69882 | 0.01025 | 2.6787 | 2.7190 |
| 4 | 60 | 2.69823 | 0.01894 | 2.6610 | 2.7354 |

Std Error uses a pooled estimate of error variance

**Means Comparisons**

**Comparisons for each pair using Student's t**

**Confidence Quantile**

| **t** | **Alpha** |
| --- | --- |
| 1.96420 | 0.05 |

**LSD Threshold Matrix**

| Abs(Dif)-LSD | **2** | **3** | **4** | **1** | **0** |
| --- | --- | --- | --- | --- | --- |
| 2 | -0.02509 | -0.00108 | -0.01488 | -0.02614 | -0.10405 |
| 3 | -0.00108 | -0.02847 | -0.04172 | -0.05274 | -0.13011 |
| 4 | -0.01488 | -0.04172 | -0.05262 | -0.06173 | -0.13403 |
| 1 | -0.02614 | -0.05274 | -0.06173 | -0.07095 | -0.13852 |
| 0 | -0.10405 | -0.13011 | -0.13403 | -0.13852 | -0.20380 |

Positive values show pairs of means that are significantly different.

**Connecting Letters Report**

| **Level** |  |  |  |  |  |  | **Mean** |
| --- | --- | --- | --- | --- | --- | --- | --- |
| 2 | A |  |  |  |  |  | 2.7245738 |
| 3 | A |  |  |  |  |  | 2.6988203 |
| 4 | A |  |  |  |  |  | 2.6982322 |
| 1 | A |  |  |  |  |  | 2.6974963 |
| 0 | A |  |  |  |  |  | 2.6834268 |

Levels not connected by same letter are significantly different.

**Ordered Differences Report**

| **Level** | **- Level** | **Difference** | **Std Err Dif** | **Lower CL** | **Upper CL** | **p-Value** |  |
| --- | --- | --- | --- | --- | --- | --- | --- |
| 2 | 0 | 0.0411470 | 0.0739209 | -0.104049 | 0.1863426 | 0.5780 |  |
| 2 | 1 | 0.0270775 | 0.0270926 | -0.026138 | 0.0802929 | 0.3180 |  |
| 2 | 4 | 0.0263415 | 0.0209859 | -0.014879 | 0.0675620 | 0.2099 |  |
| 2 | 3 | 0.0257534 | 0.0136597 | -0.001077 | 0.0525837 | 0.0599 |  |
| 3 | 0 | 0.0153936 | 0.0740795 | -0.130114 | 0.1609007 | 0.8355 |  |
| 4 | 0 | 0.0148054 | 0.0757733 | -0.134029 | 0.1636396 | 0.8452 |  |
| 1 | 0 | 0.0140695 | 0.0776866 | -0.138523 | 0.1666616 | 0.8563 |  |
| 3 | 1 | 0.0013241 | 0.0275224 | -0.052736 | 0.0553837 | 0.9616 |  |
| 4 | 1 | 0.0007359 | 0.0318010 | -0.061728 | 0.0631995 | 0.9815 |  |
| 3 | 4 | 0.0005881 | 0.0215379 | -0.041717 | 0.0428928 | 0.9782 |  |

**Product-Limit Survival Fit**

**Survival Plot**

Time to event:

RFS.delay

Censored by

rfs.event

Censor Code

1

Grouped by

NRIP2_Q

**Summary**

| **Group** | **Number failed** | **Number censored** | **Mean** |  | **Std Error** |
| --- | --- | --- | --- | --- | --- |
| 0 | 44 | 93 | 45.0324 | Biased | 2.11722 |
| 1 | 46 | 92 | 66.2327 | Biased | 3.32559 |
| 2 | 40 | 102 | 56.2992 | Biased | 2.47213 |
| 3 | 47 | 93 | 82.2917 | Biased | 4.45369 |
| Combined | 177 | 380 | 82.9813 | Biased | 2.2269 |

**Quantiles**

| **Group** | **Median Time** | **Lower 95%** | **Upper 95%** | **25% Failures** | **75% Failures** |
| --- | --- | --- | --- | --- | --- |
| 0 | . | . | . | 23 | . |
| 1 | . | 93 | . | 21 | . |
| 2 | . | . | . | 36 | . |
| 3 | . | 119 | . | 17 | . |
| Combined | . | . | . | 22 | . |

**Tests Between Groups**

| **Test** | **ChiSquare** | **DF** | **Prob>ChiSq** |
| --- | --- | --- | --- |
| Log-Rank | 0.8739 | 3 | 0.8317 |
| Wilcoxon | 0.9406 | 3 | 0.8156 |

**Product-Limit Survival Fit chemotherapy.adjuvant=N**

**Survival Plot**

Time to event:

RFS.delay

Censored by

rfs.event

Censor Code

1

Grouped by

NRIP2_Q

**Summary**

| **Group** | **Number failed** | **Number censored** | **Mean** |  | **Std Error** |
| --- | --- | --- | --- | --- | --- |
| 0 | 17 | 63 | 45.0744 | Biased | 1.92903 |
| 1 | 22 | 67 | 72.9436 | Biased | 3.90158 |
| 2 | 12 | 59 | 50.0599 | Biased | 1.79544 |
| 3 | 20 | 49 | 34.0259 | Biased | 1.8756 |
| Combined | 71 | 238 | 73.7454 | Biased | 2.02594 |

**Quantiles**

| **Group** | **Median Time** | **Lower 95%** | **Upper 95%** | **25% Failures** | **75% Failures** |
| --- | --- | --- | --- | --- | --- |
| 0 | . | . | . | 53 | . |
| 1 | . | . | . | 45 | . |
| 2 | . | . | . | . | . |
| 3 | . | . | . | 24 | . |
| Combined | . | . | . | 48 | . |

**Tests Between Groups**

| **Test** | **ChiSquare** | **DF** | **Prob>ChiSq** |
| --- | --- | --- | --- |
| Log-Rank | 3.0971 | 3 | 0.3769 |
| Wilcoxon | 4.5811 | 3 | 0.2052 |

**Product-Limit Survival Fit chemotherapy.adjuvant=Y**

**Survival Plot**

Time to event:

RFS.delay

Censored by

rfs.event

Censor Code

1

Grouped by

NRIP2_Q

**Summary**

| **Group** | **Number failed** | **Number censored** | **Mean** |  | **Std Error** |
| --- | --- | --- | --- | --- | --- |
| 0 | 18 | 30 | 43.9383 | Biased | 3.5766 |
| 1 | 22 | 25 | 49.853 | Biased | 4.83599 |
| 2 | 24 | 42 | 51.3721 | Biased | 3.93796 |
| 3 | 27 | 44 | 78.6613 | Biased | 6.3886 |
| Combined | 91 | 141 | 76.2136 | Biased | 3.52285 |

**Quantiles**

| **Group** | **Median Time** | **Lower 95%** | **Upper 95%** | **25% Failures** | **75% Failures** |
| --- | --- | --- | --- | --- | --- |
| 0 | . | 34 | . | 19 | . |
| 1 | 78 | 21 | . | 18 | . |
| 2 | . | 54 | . | 18 | . |
| 3 | 119 | 33 | . | 14 | . |
| Combined | . | 78 | . | 17 | . |

**Tests Between Groups**

| **Test** | **ChiSquare** | **DF** | **Prob>ChiSq** |
| --- | --- | --- | --- |
| Log-Rank | 0.6955 | 3 | 0.8743 |
| Wilcoxon | 0.3296 | 3 | 0.9544 |

**Product-Limit Survival Fit cit-coloncancermolecularsubtype=C1**

**Survival Plot**

Time to event:

RFS.delay

Censored by

rfs.event

Censor Code

1

Grouped by

NRIP2_Q

**Summary**

| **Group** | **Number failed** | **Number censored** | **Mean** |  | **Std Error** |
| --- | --- | --- | --- | --- | --- |
| 0 | 6 | 17 | 49.289 | Biased | 5.06292 |
| 1 | 11 | 23 | 50.7385 | Biased | 4.35052 |
| 2 | 9 | 21 | 28.9107 | Biased | 2.75265 |
| 3 | 11 | 16 | 24.52 | Biased | 2.38159 |
| Combined | 37 | 77 | 48.6673 | Biased | 2.47932 |

**Quantiles**

| **Group** | **Median Time** | **Lower 95%** | **Upper 95%** | **25% Failures** | **75% Failures** |
| --- | --- | --- | --- | --- | --- |
| 0 | . | 61 | . | 61 | . |
| 1 | . | 45 | . | 32 | . |
| 2 | . | 28 | . | 28 | . |
| 3 | . | 15 | . | 15 | . |
| Combined | . | . | . | 23 | . |

**Tests Between Groups**

| **Test** | **ChiSquare** | **DF** | **Prob>ChiSq** |
| --- | --- | --- | --- |
| Log-Rank | 1.5288 | 3 | 0.6757 |
| Wilcoxon | 1.6681 | 3 | 0.6441 |

**Product-Limit Survival Fit cit-coloncancermolecularsubtype=C2**

**Survival Plot**

Time to event:

RFS.delay

Censored by

rfs.event

Censor Code

1

Grouped by

NRIP2_Q

**Summary**

| **Group** | **Number failed** | **Number censored** | **Mean** |  | **Std Error** |
| --- | --- | --- | --- | --- | --- |
| 0 | 6 | 27 | 45.8385 | Biased | 3.26018 |
| 1 | 6 | 25 | 25.0345 | Biased | 1.36864 |
| 2 | 3 | 17 | 19.6941 | Biased | 0.3023 |
| 3 | 4 | 13 | 100 | Biased | 11.5133 |
| Combined | 19 | 82 | 98.9233 | Biased | 4.39045 |

**Quantiles**

| **Group** | **Median Time** | **Lower 95%** | **Upper 95%** | **25% Failures** | **75% Failures** |
| --- | --- | --- | --- | --- | --- |
| 0 | . | . | . | . | . |
| 1 | . | . | . | . | . |
| 2 | . | . | . | . | . |
| 3 | . | 119 | . | 119 | . |
| Combined | . | 119 | . | 119 | . |

**Tests Between Groups**

| **Test** | **ChiSquare** | **DF** | **Prob>ChiSq** |
| --- | --- | --- | --- |
| Log-Rank | 0.2493 | 3 | 0.9693 |
| Wilcoxon | 0.3342 | 3 | 0.9535 |

**Product-Limit Survival Fit cit-coloncancermolecularsubtype=C3**

**Survival Plot**

Time to event:

RFS.delay

Censored by

rfs.event

Censor Code

1

Grouped by

NRIP2_Q

**Summary**

| **Group** | **Number failed** | **Number censored** | **Mean** |  | **Std Error** |
| --- | --- | --- | --- | --- | --- |
| 0 | 7 | 17 | 41.4556 | Biased | 4.54385 |
| 1 | 5 | 11 | 36.0852 | Biased | 4.51251 |
| 2 | 2 | 15 | 42.4375 | Biased | 3.50885 |
| 3 | 5 | 9 | 17.5143 | Biased | 1.70652 |
| Combined | 19 | 52 | 42.3918 | Biased | 2.34957 |

**Quantiles**

| **Group** | **Median Time** | **Lower 95%** | **Upper 95%** | **25% Failures** | **75% Failures** |
| --- | --- | --- | --- | --- | --- |
| 0 | . | 53 | . | 34 | . |
| 1 | . | 25 | . | 25 | . |
| 2 | . | 45 | . | . | . |
| 3 | . | 11 | . | 13 | . |
| Combined | . | . | . | 45 | . |

**Tests Between Groups**

| **Test** | **ChiSquare** | **DF** | **Prob>ChiSq** |
| --- | --- | --- | --- |
| Log-Rank | 2.3294 | 3 | 0.5069 |
| Wilcoxon | 2.4259 | 3 | 0.4888 |

**Product-Limit Survival Fit cit-coloncancermolecularsubtype=C4**

**Survival Plot**

Time to event:

RFS.delay

Censored by

rfs.event

Censor Code

1

Grouped by

NRIP2_Q

**Summary**

| **Group** | **Number failed** | **Number censored** | **Mean** |  | **Std Error** |
| --- | --- | --- | --- | --- | --- |
| 0 | 7 | 6 | 18.7552 | Biased | 4.3518 |
| 1 | 10 | 3 | 28.6838 | Biased | 11.1154 |
| 2 | 6 | 8 | 35.8042 | Biased | 7.79518 |
| 3 | 6 | 13 | 15.2961 | Biased | 1.25569 |
| Combined | 29 | 30 | 49.609 | Biased | 5.86862 |

**Quantiles**

| **Group** | **Median Time** | **Lower 95%** | **Upper 95%** | **25% Failures** | **75% Failures** |
| --- | --- | --- | --- | --- | --- |
| 0 | 21 | 1 | . | 5 | . |
| 1 | 18 | 0 | 41 | 3 | 41 |
| 2 | 56 | 3 | . | 13 | . |
| 3 | . | 14 | . | 14 | . |
| Combined | 41 | 14 | . | 6 | . |

**Tests Between Groups**

| **Test** | **ChiSquare** | **DF** | **Prob>ChiSq** |
| --- | --- | --- | --- |
| Log-Rank | 7.7750 | 3 | 0.0509 |
| Wilcoxon | 6.0812 | 3 | 0.1077 |

**Product-Limit Survival Fit cit-coloncancermolecularsubtype=C5**

**Survival Plot**

Time to event:

RFS.delay

Censored by

rfs.event

Censor Code

1

Grouped by

NRIP2_Q

**Summary**

| **Group** | **Number failed** | **Number censored** | **Mean** |  | **Std Error** |
| --- | --- | --- | --- | --- | --- |
| 0 | 15 | 21 | 33.7504 | Biased | 3.30807 |
| 1 | 9 | 24 | 61.7399 | Biased | 5.37676 |
| 2 | 13 | 32 | 57.8681 | Biased | 4.24787 |
| 3 | 10 | 28 | 29.5556 | Biased | 1.80666 |
| Combined | 47 | 105 | 58.1243 | Biased | 2.52558 |

**Quantiles**

| **Group** | **Median Time** | **Lower 95%** | **Upper 95%** | **25% Failures** | **75% Failures** |
| --- | --- | --- | --- | --- | --- |
| 0 | 48 | 23 | . | 21 | . |
| 1 | . | 78 | . | 38 | . |
| 2 | . | 74 | . | 39 | . |
| 3 | . | . | . | 35 | . |
| Combined | . | . | . | 32 | . |

**Tests Between Groups**

| **Test** | **ChiSquare** | **DF** | **Prob>ChiSq** |
| --- | --- | --- | --- |
| Log-Rank | 4.4436 | 3 | 0.2174 |
| Wilcoxon | 4.2456 | 3 | 0.2361 |

**Product-Limit Survival Fit cit-coloncancermolecularsubtype=C6**

**Survival Plot**

Time to event:

RFS.delay

Censored by

rfs.event

Censor Code

1

Grouped by

NRIP2_Q

**Summary**

| **Group** | **Number failed** | **Number censored** | **Mean** |  | **Std Error** |
| --- | --- | --- | --- | --- | --- |
| 0 | 3 | 5 | 15.375 | Biased | 2.88805 |
| 1 | 5 | 6 | 22.1625 | Biased | 2.25693 |
| 2 | 7 | 9 | 24.6875 | Biased | 4.09157 |
| 3 | 11 | 14 | 19.784 | Biased | 1.77286 |
| Combined | 26 | 34 | 25.4218 | Biased | 1.76068 |

**Quantiles**

| **Group** | **Median Time** | **Lower 95%** | **Upper 95%** | **25% Failures** | **75% Failures** |
| --- | --- | --- | --- | --- | --- |
| 0 | . | 0 | . | 14 | . |
| 1 | 27 | 9 | . | 18 | . |
| 2 | . | 6 | . | 7 | . |
| 3 | . | 14 | . | 13 | . |
| Combined | . | 20 | . | 13 | . |

**Tests Between Groups**

| **Test** | **ChiSquare** | **DF** | **Prob>ChiSq** |
| --- | --- | --- | --- |
| Log-Rank | 0.1326 | 3 | 0.9877 |
| Wilcoxon | 0.1607 | 3 | 0.9837 |

**Product-Limit Survival Fit Characteristics=discovery**

**Survival Plot**

Time to event:

RFS.delay

Censored by

rfs.event

Censor Code

1

Grouped by

NRIP2_Q

**Summary**

| **Group** | **Number failed** | **Number censored** | **Mean** |  | **Std Error** |
| --- | --- | --- | --- | --- | --- |
| 0 | 31 | 76 | 46.7534 | Biased | 2.34982 |
| 1 | 42 | 73 | 63.3458 | Biased | 3.75999 |
| 2 | 29 | 78 | 57.152 | Biased | 2.80863 |
| 3 | 39 | 72 | 80.4703 | Biased | 5.08277 |
| Combined | 141 | 299 | 82.3019 | Biased | 2.53383 |

**Quantiles**

| **Group** | **Median Time** | **Lower 95%** | **Upper 95%** | **25% Failures** | **75% Failures** |
| --- | --- | --- | --- | --- | --- |
| 0 | . | . | . | 36 | . |
| 1 | . | 78 | . | 19 | . |
| 2 | . | . | . | 37 | . |
| 3 | . | 119 | . | 17 | . |
| Combined | . | . | . | 23 | . |

**Tests Between Groups**

| **Test** | **ChiSquare** | **DF** | **Prob>ChiSq** |
| --- | --- | --- | --- |
| Log-Rank | 2.2003 | 3 | 0.5319 |
| Wilcoxon | 1.8488 | 3 | 0.6044 |

**Product-Limit Survival Fit Characteristics=validation**

**Survival Plot**

Time to event:

RFS.delay

Censored by

rfs.event

Censor Code

1

Grouped by

NRIP2_Q

**Summary**

| **Group** | **Number failed** | **Number censored** | **Mean** |  | **Std Error** |
| --- | --- | --- | --- | --- | --- |
| 0 | 13 | 17 | 25.0017 | Biased | 2.36022 |
| 1 | 4 | 19 | 18.3478 | Biased | 0.41161 |
| 2 | 11 | 24 | 34.5868 | Biased | 3.04746 |
| 3 | 8 | 21 | 20.0015 | Biased | 1.46432 |
| Combined | 36 | 81 | 34.8105 | Biased | 1.52884 |

**Quantiles**

| **Group** | **Median Time** | **Lower 95%** | **Upper 95%** | **25% Failures** | **75% Failures** |
| --- | --- | --- | --- | --- | --- |
| 0 | . | 19 | . | 19 | . |
| 1 | . | . | . | . | . |
| 2 | . | 45 | . | 22 | . |
| 3 | . | . | . | 24 | . |
| Combined | . | . | . | 19 | . |

**Tests Between Groups**

| **Test** | **ChiSquare** | **DF** | **Prob>ChiSq** |
| --- | --- | --- | --- |
| Log-Rank | 4.8743 | 3 | 0.1812 |
| Wilcoxon | 4.5045 | 3 | 0.2119 |

**Product-Limit Survival Fit MMR.status=dMMR**

**Survival Plot**

Time to event:

RFS.delay

Censored by

rfs.event

Censor Code

1

Grouped by

NRIP2_Q

**Summary**

| **Group** | **Number failed** | **Number censored** | **Mean** |  | **Std Error** |
| --- | --- | --- | --- | --- | --- |
| 0 | 4 | 24 | 9.44444 | Biased | 0.34919 |
| 1 | 3 | 14 | 15.8667 | Biased | 1.11275 |
| 2 | 1 | 13 | 20 | Biased | . |
| 3 | 2 | 11 | 40.1538 | Biased | 3.86716 |
| Combined | 10 | 62 | 38.5236 | Biased | 1.48426 |

**Quantiles**

| **Group** | **Median Time** | **Lower 95%** | **Upper 95%** | **25% Failures** | **75% Failures** |
| --- | --- | --- | --- | --- | --- |
| 0 | . | . | . | . | . |
| 1 | . | 17 | . | . | . |
| 2 | . | . | . | . | . |
| 3 | . | 43 | . | . | . |
| Combined | . | . | . | . | . |

**Tests Between Groups**

| **Test** | **ChiSquare** | **DF** | **Prob>ChiSq** |
| --- | --- | --- | --- |
| Log-Rank | 0.8463 | 3 | 0.8384 |
| Wilcoxon | 1.0972 | 3 | 0.7777 |

**Product-Limit Survival Fit MMR.status=pMMR**

**Survival Plot**

Time to event:

RFS.delay

Censored by

rfs.event

Censor Code

1

Grouped by

NRIP2_Q

**Summary**

| **Group** | **Number failed** | **Number censored** | **Mean** |  | **Std Error** |
| --- | --- | --- | --- | --- | --- |
| 0 | 39 | 62 | 41.7127 | Biased | 2.58682 |
| 1 | 41 | 67 | 62.8661 | Biased | 3.84444 |
| 2 | 34 | 77 | 54.8315 | Biased | 2.85806 |
| 3 | 43 | 76 | 26.4175 | Biased | 1.14575 |
| Combined | 157 | 282 | 62.9279 | Biased | 1.93103 |

**Quantiles**

| **Group** | **Median Time** | **Lower 95%** | **Upper 95%** | **25% Failures** | **75% Failures** |
| --- | --- | --- | --- | --- | --- |
| 0 | . | 37 | . | 19 | . |
| 1 | . | 66 | . | 19 | . |
| 2 | . | . | . | 28 | . |
| 3 | . | . | . | 14 | . |
| Combined | . | . | . | 19 | . |

**Tests Between Groups**

| **Test** | **ChiSquare** | **DF** | **Prob>ChiSq** |
| --- | --- | --- | --- |
| Log-Rank | 1.7838 | 3 | 0.6185 |
| Wilcoxon | 2.1891 | 3 | 0.5341 |

**Product-Limit Survival Fit tnm.stage=0**

**Survival Plot**

Time to event:

RFS.delay

Censored by

rfs.event

Censor Code

1

Grouped by

NRIP2_Q

**Summary**

| **Group** | **Number failed** | **Number censored** | **Mean** |  | **Std Error** |
| --- | --- | --- | --- | --- | --- |
| 0 | 0 | 1 | . |  | . |
| 2 | 0 | 2 | . |  | . |
| 3 | 0 | 1 | . |  | . |
| Combined | 0 | 4 | . |  | . |

**Quantiles**

| **Group** | **Median Time** | **Lower 95%** | **Upper 95%** | **25% Failures** | **75% Failures** |
| --- | --- | --- | --- | --- | --- |
| 0 | . | . | . | . | . |
| 2 | . | . | . | . | . |
| 3 | . | . | . | . | . |
| Combined | . | . | . | . | . |

**Tests Between Groups**

| **Test** | **ChiSquare** | **DF** | **Prob>ChiSq** |
| --- | --- | --- | --- |
| Log-Rank | 0.0000 | 0 | <.0001* |
| Wilcoxon | 0.0000 | 0 | <.0001* |

**Product-Limit Survival Fit tnm.stage=1**

**Survival Plot**

Time to event:

RFS.delay

Censored by

rfs.event

Censor Code

1

Grouped by

NRIP2_Q

**Summary**

| **Group** | **Number failed** | **Number censored** | **Mean** |  | **Std Error** |
| --- | --- | --- | --- | --- | --- |
| 0 | 0 | 10 | . |  | . |
| 1 | 1 | 9 | 16 | Biased | . |
| 2 | 0 | 4 | . |  | . |
| 3 | 0 | 8 | . |  | . |
| Combined | 1 | 31 | 16 | Biased | . |

**Quantiles**

| **Group** | **Median Time** | **Lower 95%** | **Upper 95%** | **25% Failures** | **75% Failures** |
| --- | --- | --- | --- | --- | --- |
| 0 | . | . | . | . | . |
| 1 | . | 16 | . | . | . |
| 2 | . | . | . | . | . |
| 3 | . | . | . | . | . |
| Combined | . | . | . | . | . |

**Tests Between Groups**

| **Test** | **ChiSquare** | **DF** | **Prob>ChiSq** |
| --- | --- | --- | --- |
| Log-Rank | 2.1111 | 3 | 0.5497 |
| Wilcoxon | 2.1111 | 3 | 0.5497 |

**Product-Limit Survival Fit tnm.stage=2**

**Survival Plot**

Time to event:

RFS.delay

Censored by

rfs.event

Censor Code

1

Grouped by

NRIP2_Q

**Summary**

| **Group** | **Number failed** | **Number censored** | **Mean** |  | **Std Error** |
| --- | --- | --- | --- | --- | --- |
| 0 | 13 | 53 | 46.4553 | Biased | 1.92428 |
| 1 | 16 | 50 | 75.0779 | Biased | 4.2349 |
| 2 | 13 | 52 | 48.5453 | Biased | 2.23952 |
| 3 | 17 | 46 | 91.2116 | Biased | 6.16913 |
| Combined | 59 | 201 | 94.2239 | Biased | 2.86984 |

**Quantiles**

| **Group** | **Median Time** | **Lower 95%** | **Upper 95%** | **25% Failures** | **75% Failures** |
| --- | --- | --- | --- | --- | --- |
| 0 | . | . | . | . | . |
| 1 | . | 93 | . | 66 | . |
| 2 | . | . | . | . | . |
| 3 | . | 119 | . | 43 | . |
| Combined | . | . | . | 66 | . |

**Tests Between Groups**

| **Test** | **ChiSquare** | **DF** | **Prob>ChiSq** |
| --- | --- | --- | --- |
| Log-Rank | 0.7414 | 3 | 0.8634 |
| Wilcoxon | 0.9442 | 3 | 0.8147 |

**Product-Limit Survival Fit tnm.stage=3**

**Survival Plot**

Time to event:

RFS.delay

Censored by

rfs.event

Censor Code

1

Grouped by

NRIP2_Q

**Summary**

| **Group** | **Number failed** | **Number censored** | **Mean** |  | **Std Error** |
| --- | --- | --- | --- | --- | --- |
| 0 | 18 | 25 | 42.4167 | Biased | 3.76554 |
| 1 | 19 | 27 | 53.7066 | Biased | 4.57034 |
| 2 | 18 | 37 | 54.9445 | Biased | 3.97391 |
| 3 | 25 | 32 | 25.3054 | Biased | 1.66624 |
| Combined | 80 | 121 | 52.7942 | Biased | 2.25232 |

**Quantiles**

| **Group** | **Median Time** | **Lower 95%** | **Upper 95%** | **25% Failures** | **75% Failures** |
| --- | --- | --- | --- | --- | --- |
| 0 | . | 32 | . | 19 | . |
| 1 | . | 28 | . | 19 | . |
| 2 | . | 74 | . | 28 | . |
| 3 | . | 22 | . | 13 | . |
| Combined | . | 74 | . | 19 | . |

**Tests Between Groups**

| **Test** | **ChiSquare** | **DF** | **Prob>ChiSq** |
| --- | --- | --- | --- |
| Log-Rank | 1.6694 | 3 | 0.6438 |
| Wilcoxon | 2.4353 | 3 | 0.4871 |

**Product-Limit Survival Fit tnm.stage=4**

**Survival Plot**

Time to event:

RFS.delay

Censored by

rfs.event

Censor Code

1

Grouped by

NRIP2_Q

**Summary**

| **Group** | **Number failed** | **Number censored** | **Mean** |  | **Std Error** |
| --- | --- | --- | --- | --- | --- |
| 0 | 13 | 4 | 0.64706 | Biased | 0.22812 |
| 1 | 10 | 6 | 4.375 | Biased | 1.30728 |
| 2 | 9 | 7 | 8.5625 | Biased | 1.84182 |
| 3 | 5 | 6 | 12.4848 | Biased | 2.65045 |
| Combined | 37 | 23 | 8.05764 | Biased | 1.12014 |

**Quantiles**

| **Group** | **Median Time** | **Lower 95%** | **Upper 95%** | **25% Failures** | **75% Failures** |
| --- | --- | --- | --- | --- | --- |
| 0 | 0 | 0 | 2 | 0 | 2 |
| 1 | 0 | 0 | . | 0 | . |
| 2 | 12 | 0 | . | 0 | . |
| 3 | 18 | 0 | . | 0 | . |
| Combined | 1 | 0 | 18 | 0 | . |

**Tests Between Groups**

| **Test** | **ChiSquare** | **DF** | **Prob>ChiSq** |
| --- | --- | --- | --- |
| Log-Rank | 4.1880 | 3 | 0.2419 |
| Wilcoxon | 5.4465 | 3 | 0.1419 |

**Product-Limit Survival Fit location=distal**

**Survival Plot**

Time to event:

RFS.delay

Censored by

rfs.event

Censor Code

1

Grouped by

NRIP2_Q

**Summary**

| **Group** | **Number failed** | **Number censored** | **Mean** |  | **Std Error** |
| --- | --- | --- | --- | --- | --- |
| 0 | 29 | 48 | 42.6323 | Biased | 2.9344 |
| 1 | 25 | 54 | 58.7911 | Biased | 3.42355 |
| 2 | 34 | 62 | 51.6322 | Biased | 3.24932 |
| 3 | 29 | 58 | 82.8758 | Biased | 5.68794 |
| Combined | 117 | 222 | 80.1933 | Biased | 2.89924 |

**Quantiles**

| **Group** | **Median Time** | **Lower 95%** | **Upper 95%** | **25% Failures** | **75% Failures** |
| --- | --- | --- | --- | --- | --- |
| 0 | . | 48 | . | 19 | . |
| 1 | . | 78 | . | 28 | . |
| 2 | . | 56 | . | 19 | . |
| 3 | . | 119 | . | 17 | . |
| Combined | . | . | . | 21 | . |

**Tests Between Groups**

| **Test** | **ChiSquare** | **DF** | **Prob>ChiSq** |
| --- | --- | --- | --- |
| Log-Rank | 1.6638 | 3 | 0.6450 |
| Wilcoxon | 2.0785 | 3 | 0.5563 |

**Product-Limit Survival Fit location=proximal**

**Survival Plot**

Time to event:

RFS.delay

Censored by

rfs.event

Censor Code

1

Grouped by

NRIP2_Q

**Summary**

| **Group** | **Number failed** | **Number censored** | **Mean** |  | **Std Error** |
| --- | --- | --- | --- | --- | --- |
| 0 | 15 | 45 | 42.373 | Biased | 2.62068 |
| 1 | 21 | 38 | 63.8315 | Biased | 5.40783 |
| 2 | 6 | 40 | 34.6791 | Biased | 1.2372 |
| 3 | 18 | 35 | 33.0348 | Biased | 2.16479 |
| Combined | 60 | 158 | 69.8333 | Biased | 2.58674 |

**Quantiles**

| **Group** | **Median Time** | **Lower 95%** | **Upper 95%** | **25% Failures** | **75% Failures** |
| --- | --- | --- | --- | --- | --- |
| 0 | . | . | . | 37 | . |
| 1 | . | 41 | . | 16 | . |
| 2 | . | . | . | . | . |
| 3 | . | 43 | . | 20 | . |
| Combined | . | . | . | 33 | . |

**Tests Between Groups**

| **Test** | **ChiSquare** | **DF** | **Prob>ChiSq** |
| --- | --- | --- | --- |
| Log-Rank | 7.4831 | 3 | 0.0580 |
| Wilcoxon | 7.5701 | 3 | 0.0558 |

**Product-Limit Survival Fit tnm.stage 2=0-2**

**Survival Plot**

Time to event:

RFS.delay

Censored by

rfs.event

Censor Code

1

Grouped by

NRIP2_Q

**Summary**

| **Group** | **Number failed** | **Number censored** | **Mean** |  | **Std Error** |
| --- | --- | --- | --- | --- | --- |
| 0 | 13 | 64 | 47.3335 | Biased | 1.67885 |
| 1 | 17 | 59 | 76.3681 | Biased | 3.83709 |
| 2 | 13 | 58 | 49.1377 | Biased | 2.06965 |
| 3 | 17 | 55 | 94.5315 | Biased | 5.54658 |
| Combined | 60 | 236 | 96.6251 | Biased | 2.60833 |

**Quantiles**

| **Group** | **Median Time** | **Lower 95%** | **Upper 95%** | **25% Failures** | **75% Failures** |
| --- | --- | --- | --- | --- | --- |
| 0 | . | . | . | . | . |
| 1 | . | . | . | 93 | . |
| 2 | . | . | . | . | . |
| 3 | . | 119 | . | 119 | . |
| Combined | . | . | . | 119 | . |

**Tests Between Groups**

| **Test** | **ChiSquare** | **DF** | **Prob>ChiSq** |
| --- | --- | --- | --- |
| Log-Rank | 0.7423 | 3 | 0.8632 |
| Wilcoxon | 0.9473 | 3 | 0.8140 |

**Product-Limit Survival Fit tnm.stage 2=3-4**

**Survival Plot**

Time to event:

RFS.delay

Censored by

rfs.event

Censor Code

1

Grouped by

NRIP2_Q

**Summary**

| **Group** | **Number failed** | **Number censored** | **Mean** |  | **Std Error** |
| --- | --- | --- | --- | --- | --- |
| 0 | 31 | 29 | 33.8836 | Biased | 3.64538 |
| 1 | 29 | 33 | 46.5285 | Biased | 4.45046 |
| 2 | 27 | 44 | 49.8206 | Biased | 3.85236 |
| 3 | 30 | 38 | 24.496 | Biased | 1.61132 |
| Combined | 117 | 144 | 46.9689 | Biased | 2.16372 |

**Quantiles**

| **Group** | **Median Time** | **Lower 95%** | **Upper 95%** | **25% Failures** | **75% Failures** |
| --- | --- | --- | --- | --- | --- |
| 0 | 34 | 18 | . | 4 | . |
| 1 | 78 | 21 | . | 15 | . |
| 2 | . | 37 | . | 16 | . |
| 3 | . | 21 | . | 12 | . |
| Combined | 78 | 34 | . | 11 | . |

**Tests Between Groups**

| **Test** | **ChiSquare** | **DF** | **Prob>ChiSq** |
| --- | --- | --- | --- |
| Log-Rank | 3.6337 | 3 | 0.3038 |
| Wilcoxon | 4.3086 | 3 | 0.2300 |

**Product-Limit Survival Fit**

**Survival Plot**

Time to event:

RFS.delay

Censored by

rfs.event

Censor Code

1

Grouped by

RORb_43_Q

**Summary**

| **Group** | **Number failed** | **Number censored** | **Mean** |  | **Std Error** |
| --- | --- | --- | --- | --- | --- |
| 0 | 46 | 92 | 66.0875 | Biased | 3.33658 |
| 1 | 39 | 101 | 46.6631 | Biased | 2.05945 |
| 2 | 39 | 102 | 57.2836 | Biased | 2.40234 |
| 3 | 53 | 85 | 76.5306 | Biased | 4.64505 |
| Combined | 177 | 380 | 82.9813 | Biased | 2.2269 |

**Quantiles**

| **Group** | **Median Time** | **Lower 95%** | **Upper 95%** | **25% Failures** | **75% Failures** |
| --- | --- | --- | --- | --- | --- |
| 0 | . | 93 | . | 24 | . |
| 1 | . | . | . | 23 | . |
| 2 | . | . | . | 37 | . |
| 3 | . | 56 | . | 17 | . |
| Combined | . | . | . | 22 | . |

**Tests Between Groups**

| **Test** | **ChiSquare** | **DF** | **Prob>ChiSq** |
| --- | --- | --- | --- |
| Log-Rank | 4.3769 | 3 | 0.2235 |
| Wilcoxon | 3.6173 | 3 | 0.3059 |

**Product-Limit Survival Fit Sex=female**

**Survival Plot**

Time to event:

RFS.delay

Censored by

rfs.event

Censor Code

1

Grouped by

RORb_43_Q

**Summary**

| **Group** | **Number failed** | **Number censored** | **Mean** |  | **Std Error** |
| --- | --- | --- | --- | --- | --- |
| 0 | 19 | 40 | 66.4419 | Biased | 5.34513 |
| 1 | 15 | 47 | 23.5061 | Biased | 1.20987 |
| 2 | 12 | 55 | 33.1929 | Biased | 1.45778 |
| 3 | 25 | 37 | 25.9774 | Biased | 1.62735 |
| Combined | 71 | 179 | 68.9 | Biased | 2.45539 |

**Quantiles**

| **Group** | **Median Time** | **Lower 95%** | **Upper 95%** | **25% Failures** | **75% Failures** |
| --- | --- | --- | --- | --- | --- |
| 0 | . | 93 | . | 18 | . |
| 1 | . | . | . | 28 | . |
| 2 | . | . | . | . | . |
| 3 | . | 26 | . | 15 | . |
| Combined | . | . | . | 22 | . |

**Tests Between Groups**

| **Test** | **ChiSquare** | **DF** | **Prob>ChiSq** |
| --- | --- | --- | --- |
| Log-Rank | 7.9742 | 3 | 0.0465* |
| Wilcoxon | 6.5029 | 3 | 0.0895 |

**Product-Limit Survival Fit Sex=male**

**Survival Plot**

Time to event:

RFS.delay

Censored by

rfs.event

Censor Code

1

Grouped by

RORb_43_Q

**Summary**

| **Group** | **Number failed** | **Number censored** | **Mean** |  | **Std Error** |
| --- | --- | --- | --- | --- | --- |
| 0 | 27 | 52 | 56.8768 | Biased | 3.49326 |
| 1 | 24 | 54 | 45.2168 | Biased | 2.8946 |
| 2 | 27 | 47 | 52.9335 | Biased | 3.50141 |
| 3 | 28 | 48 | 78.9583 | Biased | 6.24424 |
| Combined | 106 | 201 | 79.7079 | Biased | 3.06843 |

**Quantiles**

| **Group** | **Median Time** | **Lower 95%** | **Upper 95%** | **25% Failures** | **75% Failures** |
| --- | --- | --- | --- | --- | --- |
| 0 | . | 78 | . | 28 | . |
| 1 | . | 61 | . | 19 | . |
| 2 | . | 54 | . | 21 | . |
| 3 | . | 53 | . | 17 | . |
| Combined | . | 119 | . | 21 | . |

**Tests Between Groups**

| **Test** | **ChiSquare** | **DF** | **Prob>ChiSq** |
| --- | --- | --- | --- |
| Log-Rank | 0.3300 | 3 | 0.9543 |
| Wilcoxon | 0.4202 | 3 | 0.9360 |

**Product-Limit Survival Fit cit-coloncancermolecularsubtype=C1**

**Survival Plot**

Time to event:

RFS.delay

Censored by

rfs.event

Censor Code

1

Grouped by

RORb_43_Q

**Summary**

| **Group** | **Number failed** | **Number censored** | **Mean** |  | **Std Error** |
| --- | --- | --- | --- | --- | --- |
| 0 | 6 | 14 | 27.0071 | Biased | 2.75369 |
| 1 | 13 | 19 | 42.9449 | Biased | 4.51358 |
| 2 | 5 | 23 | 57.3637 | Biased | 4.46011 |
| 3 | 13 | 21 | 27.7943 | Biased | 2.30711 |
| Combined | 37 | 77 | 48.6673 | Biased | 2.47932 |

**Quantiles**

| **Group** | **Median Time** | **Lower 95%** | **Upper 95%** | **25% Failures** | **75% Failures** |
| --- | --- | --- | --- | --- | --- |
| 0 | . | 24 | . | 24 | . |
| 1 | . | 19 | . | 19 | . |
| 2 | . | . | . | . | . |
| 3 | . | 22 | . | 15 | . |
| Combined | . | . | . | 23 | . |

**Tests Between Groups**

| **Test** | **ChiSquare** | **DF** | **Prob>ChiSq** |
| --- | --- | --- | --- |
| Log-Rank | 3.1730 | 3 | 0.3657 |
| Wilcoxon | 3.3455 | 3 | 0.3414 |

**Product-Limit Survival Fit cit-coloncancermolecularsubtype=C2**

**Survival Plot**

Time to event:

RFS.delay

Censored by

rfs.event

Censor Code

1

Grouped by

RORb_43_Q

**Summary**

| **Group** | **Number failed** | **Number censored** | **Mean** |  | **Std Error** |
| --- | --- | --- | --- | --- | --- |
| 0 | 5 | 22 | 25.7308 | Biased | 1.26917 |
| 1 | 6 | 14 | 41.081 | Biased | 5.02032 |
| 2 | 2 | 25 | 42.04 | Biased | 1.33022 |
| 3 | 6 | 21 | 98.037 | Biased | 9.27148 |
| Combined | 19 | 82 | 98.9233 | Biased | 4.39045 |

**Quantiles**

| **Group** | **Median Time** | **Lower 95%** | **Upper 95%** | **25% Failures** | **75% Failures** |
| --- | --- | --- | --- | --- | --- |
| 0 | . | . | . | . | . |
| 1 | . | 23 | . | 23 | . |
| 2 | . | . | . | . | . |
| 3 | 119 | 119 | . | 119 | . |
| Combined | . | 119 | . | 119 | . |

**Tests Between Groups**

| **Test** | **ChiSquare** | **DF** | **Prob>ChiSq** |
| --- | --- | --- | --- |
| Log-Rank | 5.5672 | 3 | 0.1347 |
| Wilcoxon | 5.0947 | 3 | 0.1650 |

**Product-Limit Survival Fit cit-coloncancermolecularsubtype=C3**

**Survival Plot**

Time to event:

RFS.delay

Censored by

rfs.event

Censor Code

1

Grouped by

RORb_43_Q

**Summary**

| **Group** | **Number failed** | **Number censored** | **Mean** |  | **Std Error** |
| --- | --- | --- | --- | --- | --- |
| 0 | 1 | 9 | 13 | Biased | . |
| 1 | 6 | 23 | 38.3086 | Biased | 3.00803 |
| 2 | 4 | 11 | 36 | Biased | 5.36656 |
| 3 | 8 | 9 | 35.9144 | Biased | 5.4767 |
| Combined | 19 | 52 | 42.3918 | Biased | 2.34957 |

**Quantiles**

| **Group** | **Median Time** | **Lower 95%** | **Upper 95%** | **25% Failures** | **75% Failures** |
| --- | --- | --- | --- | --- | --- |
| 0 | . | 13 | . | . | . |
| 1 | . | . | . | . | . |
| 2 | . | 0 | . | 45 | . |
| 3 | 53 | 6 | . | 11 | . |
| Combined | . | . | . | 45 | . |

**Tests Between Groups**

| **Test** | **ChiSquare** | **DF** | **Prob>ChiSq** |
| --- | --- | --- | --- |
| Log-Rank | 4.9990 | 3 | 0.1719 |
| Wilcoxon | 3.9302 | 3 | 0.2691 |

**Product-Limit Survival Fit cit-coloncancermolecularsubtype=C4**

**Survival Plot**

Time to event:

RFS.delay

Censored by

rfs.event

Censor Code

1

Grouped by

RORb_43_Q

**Summary**

| **Group** | **Number failed** | **Number censored** | **Mean** |  | **Std Error** |
| --- | --- | --- | --- | --- | --- |
| 0 | 9 | 11 | 58.5476 | Biased | 9.94872 |
| 1 | 2 | 10 | 13.5556 | Biased | 0.59259 |
| 2 | 10 | 1 | 10.5455 | Biased | 2.57972 |
| 3 | 8 | 8 | 32.4536 | Biased | 7.08026 |
| Combined | 29 | 30 | 49.609 | Biased | 5.86862 |

**Quantiles**

| **Group** | **Median Time** | **Lower 95%** | **Upper 95%** | **25% Failures** | **75% Failures** |
| --- | --- | --- | --- | --- | --- |
| 0 | 93 | 5 | . | 11.5 | . |
| 1 | . | 10 | . | . | . |
| 2 | 13 | 0 | 18 | 0 | 18 |
| 3 | 56 | 4 | . | 4.5 | . |
| Combined | 41 | 14 | . | 6 | . |

**Tests Between Groups**

| **Test** | **ChiSquare** | **DF** | **Prob>ChiSq** |
| --- | --- | --- | --- |
| Log-Rank | 11.9227 | 3 | 0.0077* |
| Wilcoxon | 10.6675 | 3 | 0.0137* |

**Product-Limit Survival Fit cit-coloncancermolecularsubtype=C5**

**Survival Plot**

Time to event:

RFS.delay

Censored by

rfs.event

Censor Code

1

Grouped by

RORb_43_Q

**Summary**

| **Group** | **Number failed** | **Number censored** | **Mean** |  | **Std Error** |
| --- | --- | --- | --- | --- | --- |
| 0 | 16 | 28 | 56.3789 | Biased | 4.76488 |
| 1 | 7 | 24 | 18.9301 | Biased | 1.63159 |
| 2 | 12 | 32 | 58.9087 | Biased | 4.20463 |
| 3 | 12 | 21 | 36.1028 | Biased | 3.22102 |
| Combined | 47 | 105 | 58.1243 | Biased | 2.52558 |

**Quantiles**

| **Group** | **Median Time** | **Lower 95%** | **Upper 95%** | **25% Failures** | **75% Failures** |
| --- | --- | --- | --- | --- | --- |
| 0 | . | 37 | . | 32 | . |
| 1 | . | . | . | . | . |
| 2 | . | 74 | . | 38 | . |
| 3 | . | 30 | . | 18 | . |
| Combined | . | . | . | 32 | . |

**Tests Between Groups**

| **Test** | **ChiSquare** | **DF** | **Prob>ChiSq** |
| --- | --- | --- | --- |
| Log-Rank | 1.6491 | 3 | 0.6483 |
| Wilcoxon | 1.2463 | 3 | 0.7419 |

**Product-Limit Survival Fit cit-coloncancermolecularsubtype=C6**

**Survival Plot**

Time to event:

RFS.delay

Censored by

rfs.event

Censor Code

1

Grouped by

RORb_43_Q

**Summary**

| **Group** | **Number failed** | **Number censored** | **Mean** |  | **Std Error** |
| --- | --- | --- | --- | --- | --- |
| 0 | 9 | 8 | 18.4118 | Biased | 2.66035 |
| 1 | 5 | 11 | 14.0625 | Biased | 1.98251 |
| 2 | 6 | 10 | 28.4667 | Biased | 3.06875 |
| 3 | 6 | 5 | 20.1039 | Biased | 2.38731 |
| Combined | 26 | 34 | 25.4218 | Biased | 1.76068 |

**Quantiles**

| **Group** | **Median Time** | **Lower 95%** | **Upper 95%** | **25% Failures** | **75% Failures** |
| --- | --- | --- | --- | --- | --- |
| 0 | 27 | 9 | . | 10 | . |
| 1 | . | 9 | . | 13.5 | . |
| 2 | . | 14 | . | 14 | . |
| 3 | 26 | 11 | . | 11 | . |
| Combined | . | 20 | . | 13 | . |

**Tests Between Groups**

| **Test** | **ChiSquare** | **DF** | **Prob>ChiSq** |
| --- | --- | --- | --- |
| Log-Rank | 1.7603 | 3 | 0.6236 |
| Wilcoxon | 1.3854 | 3 | 0.7090 |

**Product-Limit Survival Fit Characteristics=discovery**

**Survival Plot**

Time to event:

RFS.delay

Censored by

rfs.event

Censor Code

1

Grouped by

RORb_43_Q

**Summary**

| **Group** | **Number failed** | **Number censored** | **Mean** |  | **Std Error** |
| --- | --- | --- | --- | --- | --- |
| 0 | 36 | 70 | 65.4505 | Biased | 3.85452 |
| 1 | 28 | 78 | 47.4839 | Biased | 2.34132 |
| 2 | 33 | 84 | 57.1452 | Biased | 2.64779 |
| 3 | 44 | 67 | 74.5888 | Biased | 5.29438 |
| Combined | 141 | 299 | 82.3019 | Biased | 2.53383 |

**Quantiles**

| **Group** | **Median Time** | **Lower 95%** | **Upper 95%** | **25% Failures** | **75% Failures** |
| --- | --- | --- | --- | --- | --- |
| 0 | . | 93 | . | 27 | . |
| 1 | . | . | . | 28 | . |
| 2 | . | . | . | 37 | . |
| 3 | 119 | 48 | . | 15 | . |
| Combined | . | . | . | 23 | . |

**Tests Between Groups**

| **Test** | **ChiSquare** | **DF** | **Prob>ChiSq** |
| --- | --- | --- | --- |
| Log-Rank | 5.2801 | 3 | 0.1524 |
| Wilcoxon | 4.1645 | 3 | 0.2442 |

**Product-Limit Survival Fit Characteristics=validation**

**Survival Plot**

Time to event:

RFS.delay

Censored by

rfs.event

Censor Code

1

Grouped by

RORb_43_Q

**Summary**

| **Group** | **Number failed** | **Number censored** | **Mean** |  | **Std Error** |
| --- | --- | --- | --- | --- | --- |
| 0 | 10 | 22 | 25.8125 | Biased | 1.99633 |
| 1 | 11 | 23 | 33.7561 | Biased | 3.18726 |
| 2 | 6 | 18 | 21.2278 | Biased | 1.37483 |
| 3 | 9 | 18 | 27.4615 | Biased | 2.25534 |
| Combined | 36 | 81 | 34.8105 | Biased | 1.52884 |

**Quantiles**

| **Group** | **Median Time** | **Lower 95%** | **Upper 95%** | **25% Failures** | **75% Failures** |
| --- | --- | --- | --- | --- | --- |
| 0 | . | 32 | . | 20.5 | . |
| 1 | . | 23 | . | 19 | . |
| 2 | . | 24 | . | 24 | . |
| 3 | . | 30 | . | 19 | . |
| Combined | . | . | . | 19 | . |

**Tests Between Groups**

| **Test** | **ChiSquare** | **DF** | **Prob>ChiSq** |
| --- | --- | --- | --- |
| Log-Rank | 0.5670 | 3 | 0.9039 |
| Wilcoxon | 0.6111 | 3 | 0.8939 |

**Product-Limit Survival Fit MMR.status=dMMR**

**Survival Plot**

Time to event:

RFS.delay

Censored by

rfs.event

Censor Code

1

Grouped by

RORb_43_Q

**Summary**

| **Group** | **Number failed** | **Number censored** | **Mean** |  | **Std Error** |
| --- | --- | --- | --- | --- | --- |
| 0 | 2 | 15 | 19.6 | Biased | 0.5465 |
| 1 | 2 | 12 | 16.3636 | Biased | 0.85807 |
| 2 | 1 | 19 | 43 | Biased | . |
| 3 | 5 | 16 | 5.71429 | Biased | 0.17075 |
| Combined | 10 | 62 | 38.5236 | Biased | 1.48426 |

**Quantiles**

| **Group** | **Median Time** | **Lower 95%** | **Upper 95%** | **25% Failures** | **75% Failures** |
| --- | --- | --- | --- | --- | --- |
| 0 | . | . | . | . | . |
| 1 | . | 17 | . | . | . |
| 2 | . | . | . | . | . |
| 3 | . | . | . | . | . |
| Combined | . | . | . | . | . |

**Tests Between Groups**

| **Test** | **ChiSquare** | **DF** | **Prob>ChiSq** |
| --- | --- | --- | --- |
| Log-Rank | 3.1088 | 3 | 0.3752 |
| Wilcoxon | 4.4393 | 3 | 0.2178 |

**Product-Limit Survival Fit MMR.status=pMMR**

**Survival Plot**

Time to event:

RFS.delay

Censored by

rfs.event

Censor Code

1

Grouped by

RORb_43_Q

**Summary**

| **Group** | **Number failed** | **Number censored** | **Mean** |  | **Std Error** |
| --- | --- | --- | --- | --- | --- |
| 0 | 42 | 73 | 64.1 | Biased | 3.6875 |
| 1 | 34 | 83 | 45.9379 | Biased | 2.29453 |
| 2 | 36 | 68 | 52.421 | Biased | 3.04905 |
| 3 | 45 | 58 | 37.0536 | Biased | 2.2678 |
| Combined | 157 | 282 | 62.9279 | Biased | 1.93103 |

**Quantiles**

| **Group** | **Median Time** | **Lower 95%** | **Upper 95%** | **25% Failures** | **75% Failures** |
| --- | --- | --- | --- | --- | --- |
| 0 | . | 93 | . | 21 | . |
| 1 | . | . | . | 21 | . |
| 2 | . | 74 | . | 19 | . |
| 3 | . | 30 | . | 13 | . |
| Combined | . | . | . | 19 | . |

**Tests Between Groups**

| **Test** | **ChiSquare** | **DF** | **Prob>ChiSq** |
| --- | --- | --- | --- |
| Log-Rank | 4.7575 | 3 | 0.1904 |
| Wilcoxon | 3.1924 | 3 | 0.3629 |

**Product-Limit Survival Fit tnm.stage=0**

**Survival Plot**

Time to event:

RFS.delay

Censored by

rfs.event

Censor Code

1

Grouped by

RORb_43_Q

**Summary**

| **Group** | **Number failed** | **Number censored** | **Mean** |  | **Std Error** |
| --- | --- | --- | --- | --- | --- |
| 0 | 0 | 1 | . |  | . |
| 1 | 0 | 2 | . |  | . |
| 3 | 0 | 1 | . |  | . |
| Combined | 0 | 4 | . |  | . |

**Quantiles**

| **Group** | **Median Time** | **Lower 95%** | **Upper 95%** | **25% Failures** | **75% Failures** |
| --- | --- | --- | --- | --- | --- |
| 0 | . | . | . | . | . |
| 1 | . | . | . | . | . |
| 3 | . | . | . | . | . |
| Combined | . | . | . | . | . |

**Tests Between Groups**

| **Test** | **ChiSquare** | **DF** | **Prob>ChiSq** |
| --- | --- | --- | --- |
| Log-Rank | 0.0000 | 0 | <.0001* |
| Wilcoxon | 0.0000 | 0 | <.0001* |

**Product-Limit Survival Fit tnm.stage=1**

**Survival Plot**

Time to event:

RFS.delay

Censored by

rfs.event

Censor Code

1

Grouped by

RORb_43_Q

**Summary**

| **Group** | **Number failed** | **Number censored** | **Mean** |  | **Std Error** |
| --- | --- | --- | --- | --- | --- |
| 0 | 0 | 12 | . |  | . |
| 1 | 1 | 2 | 16 | Biased | . |
| 2 | 0 | 9 | . |  | . |
| 3 | 0 | 8 | . |  | . |
| Combined | 1 | 31 | 16 | Biased | . |

**Quantiles**

| **Group** | **Median Time** | **Lower 95%** | **Upper 95%** | **25% Failures** | **75% Failures** |
| --- | --- | --- | --- | --- | --- |
| 0 | . | . | . | . | . |
| 1 | . | 16 | . | 16 | . |
| 2 | . | . | . | . | . |
| 3 | . | . | . | . | . |
| Combined | . | . | . | . | . |

**Tests Between Groups**

| **Test** | **ChiSquare** | **DF** | **Prob>ChiSq** |
| --- | --- | --- | --- |
| Log-Rank | 8.3333 | 3 | 0.0396* |
| Wilcoxon | 8.3333 | 3 | 0.0396* |

**Product-Limit Survival Fit tnm.stage=2**

**Survival Plot**

Time to event:

RFS.delay

Censored by

rfs.event

Censor Code

1

Grouped by

RORb_43_Q

**Summary**

| **Group** | **Number failed** | **Number censored** | **Mean** |  | **Std Error** |
| --- | --- | --- | --- | --- | --- |
| 0 | 12 | 44 | 77.1673 | Biased | 4.49517 |
| 1 | 12 | 48 | 45.6795 | Biased | 2.2274 |
| 2 | 10 | 57 | 60.5621 | Biased | 1.88107 |
| 3 | 25 | 52 | 84.3512 | Biased | 5.95523 |
| Combined | 59 | 201 | 94.2239 | Biased | 2.86984 |

**Quantiles**

| **Group** | **Median Time** | **Lower 95%** | **Upper 95%** | **25% Failures** | **75% Failures** |
| --- | --- | --- | --- | --- | --- |
| 0 | . | . | . | 93 | . |
| 1 | . | . | . | . | . |
| 2 | . | . | . | . | . |
| 3 | . | 119 | . | 21 | . |
| Combined | . | . | . | 66 | . |

**Tests Between Groups**

| **Test** | **ChiSquare** | **DF** | **Prob>ChiSq** |
| --- | --- | --- | --- |
| Log-Rank | 7.5046 | 3 | 0.0574 |
| Wilcoxon | 8.8557 | 3 | 0.0313* |

**Product-Limit Survival Fit tnm.stage=3**

**Survival Plot**

Time to event:

RFS.delay

Censored by

rfs.event

Censor Code

1

Grouped by

RORb_43_Q

**Summary**

| **Group** | **Number failed** | **Number censored** | **Mean** |  | **Std Error** |
| --- | --- | --- | --- | --- | --- |
| 0 | 27 | 29 | 48.8589 | Biased | 4.33027 |
| 1 | 15 | 45 | 49.0896 | Biased | 2.89652 |
| 2 | 15 | 30 | 54.9607 | Biased | 4.31196 |
| 3 | 23 | 17 | 31.3424 | Biased | 3.36659 |
| Combined | 80 | 121 | 52.7942 | Biased | 2.25232 |

**Quantiles**

| **Group** | **Median Time** | **Lower 95%** | **Upper 95%** | **25% Failures** | **75% Failures** |
| --- | --- | --- | --- | --- | --- |
| 0 | 78 | 27 | . | 18 | . |
| 1 | . | . | . | 61 | . |
| 2 | . | 38 | . | 24 | . |
| 3 | 34 | 11 | . | 8 | . |
| Combined | . | 74 | . | 19 | . |

**Tests Between Groups**

| **Test** | **ChiSquare** | **DF** | **Prob>ChiSq** |
| --- | --- | --- | --- |
| Log-Rank | 11.8883 | 3 | 0.0078* |
| Wilcoxon | 11.8100 | 3 | 0.0081* |

**Product-Limit Survival Fit tnm.stage=4**

**Survival Plot**

Time to event:

RFS.delay

Censored by

rfs.event

Censor Code

1

Grouped by

RORb_43_Q

**Summary**

| **Group** | **Number failed** | **Number censored** | **Mean** |  | **Std Error** |
| --- | --- | --- | --- | --- | --- |
| 0 | 7 | 6 | 8 | Biased | 2.25932 |
| 1 | 11 | 4 | 3 | Biased | 1.14891 |
| 2 | 14 | 6 | 7.3 | Biased | 1.92068 |
| 3 | 5 | 7 | 8.66667 | Biased | 1.97789 |
| Combined | 37 | 23 | 8.05764 | Biased | 1.12014 |

**Quantiles**

| **Group** | **Median Time** | **Lower 95%** | **Upper 95%** | **25% Failures** | **75% Failures** |
| --- | --- | --- | --- | --- | --- |
| 0 | 15 | 0 | . | 0 | . |
| 1 | 0 | 0 | 9 | 0 | . |
| 2 | 1 | 0 | 18 | 0 | . |
| 3 | . | 0 | . | 0 | . |
| Combined | 1 | 0 | 18 | 0 | . |

**Tests Between Groups**

| **Test** | **ChiSquare** | **DF** | **Prob>ChiSq** |
| --- | --- | --- | --- |
| Log-Rank | 3.5348 | 3 | 0.3163 |
| Wilcoxon | 3.8933 | 3 | 0.2732 |

**Product-Limit Survival Fit tnm.stage 2=0-2**

**Survival Plot**

Time to event:

RFS.delay

Censored by

rfs.event

Censor Code

1

Grouped by

RORb_43_Q

**Summary**

| **Group** | **Number failed** | **Number censored** | **Mean** |  | **Std Error** |
| --- | --- | --- | --- | --- | --- |
| 0 | 12 | 57 | 80.0721 | Biased | 3.74947 |
| 1 | 13 | 52 | 45.5172 | Biased | 2.15093 |
| 2 | 10 | 66 | 61.1449 | Biased | 1.68967 |
| 3 | 25 | 61 | 87.8162 | Biased | 5.4865 |
| Combined | 60 | 236 | 96.6251 | Biased | 2.60833 |

**Quantiles**

| **Group** | **Median Time** | **Lower 95%** | **Upper 95%** | **25% Failures** | **75% Failures** |
| --- | --- | --- | --- | --- | --- |
| 0 | . | . | . | . | . |
| 1 | . | . | . | . | . |
| 2 | . | . | . | . | . |
| 3 | . | 119 | . | 26 | . |
| Combined | . | . | . | 119 | . |

**Tests Between Groups**

| **Test** | **ChiSquare** | **DF** | **Prob>ChiSq** |
| --- | --- | --- | --- |
| Log-Rank | 7.5659 | 3 | 0.0559 |
| Wilcoxon | 9.1201 | 3 | 0.0277* |

**Product-Limit Survival Fit tnm.stage 2=3-4**

**Survival Plot**

Time to event:

RFS.delay

Censored by

rfs.event

Censor Code

1

Grouped by

RORb_43_Q

**Summary**

| **Group** | **Number failed** | **Number censored** | **Mean** |  | **Std Error** |
| --- | --- | --- | --- | --- | --- |
| 0 | 34 | 35 | 45.3585 | Biased | 4.15659 |
| 1 | 26 | 49 | 42.4526 | Biased | 3.1043 |
| 2 | 29 | 36 | 44.6612 | Biased | 4.21335 |
| 3 | 28 | 24 | 30.6528 | Biased | 3.11663 |
| Combined | 117 | 144 | 46.9689 | Biased | 2.16372 |

**Quantiles**

| **Group** | **Median Time** | **Lower 95%** | **Upper 95%** | **25% Failures** | **75% Failures** |
| --- | --- | --- | --- | --- | --- |
| 0 | 41 | 21 | . | 13 | . |
| 1 | . | 61 | . | 14 | . |
| 2 | 74 | 19 | . | 13 | . |
| 3 | 34 | 13 | . | 6 | . |
| Combined | 78 | 34 | . | 11 | . |

**Tests Between Groups**

| **Test** | **ChiSquare** | **DF** | **Prob>ChiSq** |
| --- | --- | --- | --- |
| Log-Rank | 4.3547 | 3 | 0.2256 |
| Wilcoxon | 2.1392 | 3 | 0.5440 |

**Proportional Hazards Fit**

Censored By: rfs.event

**Whole Model**

|  |  |
| --- | --- |
| Number of Events | 177 |
| Number of Censorings | 380 |
| Total Number | 557 |

| **Model** | **-LogLikelihood** | **ChiSquare** | **DF** | **Prob>Chisq** |
| --- | --- | --- | --- | --- |
| Difference | 0.441 | 0.8814 | 3 | 0.8299 |
| Full | 1063.868 |  |  |  |
| Reduced | 1064.308 |  |  |  |

**Parameter Estimates**

| **Term** | **Estimate** | **Std Error** | **Lower 95%** | **Upper 95%** |  |
| --- | --- | --- | --- | --- | --- |
| NRIP2_Q[1-0] | -0.0298417 | 0.2109325 | -0.444099 | 0.385684 |  |
| NRIP2_Q[2-1] | -0.1498126 | 0.216212 | -0.577266 | 0.2734474 |  |
| NRIP2_Q[3-2] | 0.16453755 | 0.2151608 | -0.256342 | 0.5902042 |  |

**Effect Likelihood Ratio Tests**

| **Source** | **Nparm** | **DF** | **L-R ChiSquare** | **Prob>ChiSq** |  |
| --- | --- | --- | --- | --- | --- |
| NRIP2_Q | 3 | 3 | 0.88139116 | 0.8299 |  |

**Baseline Survival at mean**

**Risk Ratios**

**Risk Ratios for NRIP2_Q**

| **Level1** | **/Level2** | **Risk Ratio** | **Prob>Chisq** | **Lower 95%** | **Upper 95%** |
| --- | --- | --- | --- | --- | --- |
| 1 | 0 | 0.9705991 | 0.8875 | 0.6414021 | 1.4706199 |
| 2 | 0 | 0.835559 | 0.4106 | 0.5428125 | 1.2824338 |
| 2 | 1 | 0.8608693 | 0.4878 | 0.5614313 | 1.3144882 |
| 3 | 0 | 0.9849969 | 0.9426 | 0.6525176 | 1.4896583 |
| 3 | 1 | 1.0148338 | 0.9434 | 0.6750767 | 1.5264939 |
| 3 | 2 | 1.1788478 | 0.4437 | 0.7738772 | 1.8043569 |
| 0 | 1 | 1.0302915 | 0.8875 | 0.6799853 | 1.5590844 |
| 0 | 2 | 1.1968036 | 0.4106 | 0.7797673 | 1.8422567 |
| 1 | 2 | 1.1616166 | 0.4878 | 0.7607524 | 1.7811618 |
| 0 | 3 | 1.0152317 | 0.9426 | 0.6712949 | 1.5325257 |
| 1 | 3 | 0.985383 | 0.9434 | 0.655096 | 1.4813133 |
| 2 | 3 | 0.8482859 | 0.4437 | 0.5542141 | 1.2921947 |

**Proportional Hazards Fit tnm.stage 2=0-2**

Censored By: rfs.event

**Whole Model**

|  |  |
| --- | --- |
| Number of Events | 60 |
| Number of Censorings | 236 |
| Total Number | 296 |

| **Model** | **-LogLikelihood** | **ChiSquare** | **DF** | **Prob>Chisq** |
| --- | --- | --- | --- | --- |
| Difference | 0.3723 | 0.7447 | 3 | 0.8626 |
| Full | 322.7169 |  |  |  |
| Reduced | 323.0893 |  |  |  |

**Parameter Estimates**

| **Term** | **Estimate** | **Std Error** | **Lower 95%** | **Upper 95%** |  |
| --- | --- | --- | --- | --- | --- |
| NRIP2_Q[1-0] | 0.22915276 | 0.3686827 | -0.48964 | 0.9713748 |  |
| NRIP2_Q[2-1] | -0.1291362 | 0.3685544 | -0.871128 | 0.5893988 |  |
| NRIP2_Q[3-2] | 0.18835969 | 0.3687203 | -0.530508 | 0.9306499 |  |

**Effect Likelihood Ratio Tests**

| **Source** | **Nparm** | **DF** | **L-R ChiSquare** | **Prob>ChiSq** |  |
| --- | --- | --- | --- | --- | --- |
| NRIP2_Q | 3 | 3 | 0.74467515 | 0.8626 |  |

**Baseline Survival at mean**

**Risk Ratios**

**Risk Ratios for NRIP2_Q**

| **Level1** | **/Level2** | **Risk Ratio** | **Prob>Chisq** | **Lower 95%** | **Upper 95%** |
| --- | --- | --- | --- | --- | --- |
| 1 | 0 | 1.2575341 | 0.5326 | 0.6128468 | 2.6415735 |
| 2 | 0 | 1.1051893 | 0.7988 | 0.5073607 | 2.4074354 |
| 2 | 1 | 0.8788543 | 0.7254 | 0.4184793 | 1.8029041 |
| 3 | 0 | 1.3342593 | 0.4322 | 0.6499852 | 2.8037 |
| 3 | 1 | 1.0610124 | 0.8629 | 0.5382209 | 2.0916058 |
| 3 | 2 | 1.2072677 | 0.6082 | 0.5883059 | 2.5361569 |
| 0 | 1 | 0.795207 | 0.5326 | 0.3785622 | 1.6317292 |
| 0 | 2 | 0.9048224 | 0.7988 | 0.4153798 | 1.9709842 |
| 1 | 2 | 1.137845 | 0.7254 | 0.5546607 | 2.3896046 |
| 0 | 3 | 0.7494795 | 0.4322 | 0.3566715 | 1.5384966 |
| 1 | 3 | 0.9424961 | 0.8629 | 0.4781016 | 1.8579732 |
| 2 | 3 | 0.8283167 | 0.6082 | 0.3942974 | 1.699796 |

**Proportional Hazards Fit tnm.stage 2=3-4**

Censored By: rfs.event

**Whole Model**

|  |  |
| --- | --- |
| Number of Events | 117 |
| Number of Censorings | 144 |
| Total Number | 261 |

| **Model** | **-LogLikelihood** | **ChiSquare** | **DF** | **Prob>Chisq** |
| --- | --- | --- | --- | --- |
| Difference | 1.7033 | 3.4067 | 3 | 0.3331 |
| Full | 607.2224 |  |  |  |
| Reduced | 608.9258 |  |  |  |

**Parameter Estimates**

| **Term** | **Estimate** | **Std Error** | **Lower 95%** | **Upper 95%** |  |
| --- | --- | --- | --- | --- | --- |
| NRIP2_Q[1-0] | -0.2315166 | 0.2585482 | -0.742398 | 0.2764992 |  |
| NRIP2_Q[2-1] | -0.2478481 | 0.2675169 | -0.776819 | 0.2778349 |  |
| NRIP2_Q[3-2] | 0.17355683 | 0.2653817 | -0.347129 | 0.6990125 |  |

**Effect Likelihood Ratio Tests**

| **Source** | **Nparm** | **DF** | **L-R ChiSquare** | **Prob>ChiSq** |  |
| --- | --- | --- | --- | --- | --- |
| NRIP2_Q | 3 | 3 | 3.4066859 | 0.3331 |  |

**Baseline Survival at mean**

**Risk Ratios**

**Risk Ratios for NRIP2_Q**

| **Level1** | **/Level2** | **Risk Ratio** | **Prob>Chisq** | **Lower 95%** | **Upper 95%** |
| --- | --- | --- | --- | --- | --- |
| 1 | 0 | 0.7933295 | 0.3705 | 0.4759711 | 1.3185059 |
| 2 | 0 | 0.6191766 | 0.0686 | 0.3672719 | 1.0374596 |
| 2 | 1 | 0.7804785 | 0.3541 | 0.4598663 | 1.3202681 |
| 3 | 0 | 0.7365281 | 0.2333 | 0.4441942 | 1.2195644 |
| 3 | 1 | 0.9284013 | 0.7756 | 0.5560551 | 1.5523743 |
| 3 | 2 | 1.1895283 | 0.5127 | 0.7067141 | 2.0117651 |
| 0 | 1 | 1.2605103 | 0.3705 | 0.7584342 | 2.1009678 |
| 0 | 2 | 1.615048 | 0.0686 | 0.9638929 | 2.7227786 |
| 1 | 2 | 1.2812653 | 0.3541 | 0.7574219 | 2.174545 |
| 0 | 3 | 1.3577214 | 0.2333 | 0.8199649 | 2.2512674 |
| 1 | 3 | 1.0771205 | 0.7756 | 0.6441745 | 1.7983829 |
| 2 | 3 | 0.8406694 | 0.5127 | 0.4970759 | 1.4149993 |

**Proportional Hazards Fit**

Censored By: rfs.event

**Whole Model**

|  |  |
| --- | --- |
| Number of Events | 177 |
| Number of Censorings | 380 |
| Total Number | 557 |

| **Model** | **-LogLikelihood** | **ChiSquare** | **DF** | **Prob>Chisq** |
| --- | --- | --- | --- | --- |
| Difference | 2.096 | 4.1914 | 3 | 0.2415 |
| Full | 1062.213 |  |  |  |
| Reduced | 1064.308 |  |  |  |

**Parameter Estimates**

| **Term** | **Estimate** | **Std Error** | **Lower 95%** | **Upper 95%** |  |
| --- | --- | --- | --- | --- | --- |
| RORb_43_Q[1-0] | -0.1379864 | 0.2177015 | -0.568804 | 0.2878151 |  |
| RORb_43_Q[2-1] | -0.063314 | 0.2264887 | -0.509047 | 0.3824185 |  |
| RORb_43_Q[3-2] | 0.39528201 | 0.2110842 | -0.015568 | 0.8148336 |  |

**Effect Likelihood Ratio Tests**

| **Source** | **Nparm** | **DF** | **L-R ChiSquare** | **Prob>ChiSq** |  |
| --- | --- | --- | --- | --- | --- |
| RORb_43_Q | 3 | 3 | 4.19140161 | 0.2415 |  |

**Baseline Survival at mean**

**Risk Ratios**

**Risk Ratios for RORb_43_Q**

| **Level1** | **/Level2** | **Risk Ratio** | **Prob>Chisq** | **Lower 95%** | **Upper 95%** |
| --- | --- | --- | --- | --- | --- |
| 1 | 0 | 0.8711106 | 0.5256 | 0.5662022 | 1.3335107 |
| 2 | 0 | 0.8176668 | 0.3542 | 0.5314594 | 1.2517093 |
| 2 | 1 | 0.9386487 | 0.7798 | 0.6010682 | 1.4658254 |
| 3 | 0 | 1.214074 | 0.3352 | 0.8182907 | 1.8079424 |
| 3 | 1 | 1.3937082 | 0.1135 | 0.9242281 | 2.1200411 |
| 3 | 2 | 1.4848029 | 0.0594 | 0.9845522 | 2.2587997 |
| 0 | 1 | 1.1479599 | 0.5256 | 0.7499003 | 1.7661535 |
| 0 | 2 | 1.2229921 | 0.3542 | 0.7989075 | 1.8816113 |
| 1 | 2 | 1.0653613 | 0.7798 | 0.6822095 | 1.6637047 |
| 0 | 3 | 0.823673 | 0.3352 | 0.553115 | 1.2220596 |
| 1 | 3 | 0.7175103 | 0.1135 | 0.471689 | 1.081984 |
| 2 | 3 | 0.6734901 | 0.0594 | 0.442713 | 1.0156901 |

**Proportional Hazards Fit tnm.stage 2=0-2**

Censored By: rfs.event

**Whole Model**

|  |  |
| --- | --- |
| Number of Events | 60 |
| Number of Censorings | 236 |
| Total Number | 296 |

| **Model** | **-LogLikelihood** | **ChiSquare** | **DF** | **Prob>Chisq** |
| --- | --- | --- | --- | --- |
| Difference | 3.7212 | 7.4423 | 3 | 0.0591 |
| Full | 319.3681 |  |  |  |
| Reduced | 323.0893 |  |  |  |

**Parameter Estimates**

| **Term** | **Estimate** | **Std Error** | **Lower 95%** | **Upper 95%** |  |
| --- | --- | --- | --- | --- | --- |
| RORb_43_Q[1-0] | 0.27146273 | 0.4006869 | -0.519774 | 1.0710486 |  |
| RORb_43_Q[2-1] | -0.5934016 | 0.4210061 | -1.445326 | 0.2285021 |  |
| RORb_43_Q[3-2] | 0.92765864 | 0.3744026 | 0.2239755 | 1.7090836 |  |

**Effect Likelihood Ratio Tests**

| **Source** | **Nparm** | **DF** | **L-R ChiSquare** | **Prob>ChiSq** |  |
| --- | --- | --- | --- | --- | --- |
| RORb_43_Q | 3 | 3 | 7.44234752 | 0.0591 |  |

**Baseline Survival at mean**

**Risk Ratios**

**Risk Ratios for RORb_43_Q**

| **Level1** | **/Level2** | **Risk Ratio** | **Prob>Chisq** | **Lower 95%** | **Upper 95%** |
| --- | --- | --- | --- | --- | --- |
| 1 | 0 | 1.311882 | 0.4980 | 0.5946547 | 2.9184382 |
| 2 | 0 | 0.7247425 | 0.4509 | 0.305903 | 1.680178 |
| 2 | 1 | 0.5524449 | 0.1564 | 0.2356693 | 1.2567161 |
| 3 | 0 | 1.8325708 | 0.0760 | 0.9398866 | 3.7791983 |
| 3 | 1 | 1.3969022 | 0.3214 | 0.7266102 | 2.8165409 |
| 3 | 2 | 2.5285819 | 0.0091* | 1.2510404 | 5.5238972 |
| 0 | 1 | 0.7622637 | 0.4980 | 0.342649 | 1.6816482 |
| 0 | 2 | 1.3798004 | 0.4509 | 0.595175 | 3.2690098 |
| 1 | 2 | 1.8101352 | 0.1564 | 0.7957247 | 4.2432346 |
| 0 | 3 | 0.5456815 | 0.0760 | 0.2646064 | 1.0639582 |
| 1 | 3 | 0.7158697 | 0.3214 | 0.3550454 | 1.3762538 |
| 2 | 3 | 0.3954786 | 0.0091* | 0.1810316 | 0.7993347 |

**Proportional Hazards Fit tnm.stage 2=3-4**

Censored By: rfs.event

**Whole Model**

|  |  |
| --- | --- |
| Number of Events | 117 |
| Number of Censorings | 144 |
| Total Number | 261 |

| **Model** | **-LogLikelihood** | **ChiSquare** | **DF** | **Prob>Chisq** |
| --- | --- | --- | --- | --- |
| Difference | 2.1497 | 4.2994 | 3 | 0.2309 |
| Full | 606.7761 |  |  |  |
| Reduced | 608.9258 |  |  |  |

**Parameter Estimates**

| **Term** | **Estimate** | **Std Error** | **Lower 95%** | **Upper 95%** |  |
| --- | --- | --- | --- | --- | --- |
| RORb_43_Q[1-0] | -0.3792661 | 0.2606652 | -0.898778 | 0.1285916 |  |
| RORb_43_Q[2-1] | 0.32326295 | 0.2702367 | -0.206921 | 0.8585742 |  |
| RORb_43_Q[3-2] | 0.2171103 | 0.2651017 | -0.306189 | 0.7388251 |  |

**Effect Likelihood Ratio Tests**

| **Source** | **Nparm** | **DF** | **L-R ChiSquare** | **Prob>ChiSq** |  |
| --- | --- | --- | --- | --- | --- |
| RORb_43_Q | 3 | 3 | 4.29942464 | 0.2309 |  |

**Baseline Survival at mean**

**Risk Ratios**

**Risk Ratios for RORb_43_Q**

| **Level1** | **/Level2** | **Risk Ratio** | **Prob>Chisq** | **Lower 95%** | **Upper 95%** |
| --- | --- | --- | --- | --- | --- |
| 1 | 0 | 0.6843635 | 0.1435 | 0.4070669 | 1.1372256 |
| 2 | 0 | 0.9455362 | 0.8246 | 0.5727292 | 1.5508525 |
| 2 | 1 | 1.3816286 | 0.2313 | 0.8130839 | 2.3597936 |
| 3 | 0 | 1.1748108 | 0.5294 | 0.7074772 | 1.9350923 |
| 3 | 1 | 1.7166475 | 0.0482* | 1.0044076 | 2.9443507 |
| 3 | 2 | 1.2424811 | 0.4136 | 0.7362471 | 2.0934745 |
| 0 | 1 | 1.4612118 | 0.1435 | 0.879333 | 2.4565989 |
| 0 | 2 | 1.057601 | 0.8246 | 0.6448066 | 1.746026 |
| 1 | 2 | 0.7237835 | 0.2313 | 0.4237659 | 1.2298854 |
| 0 | 3 | 0.8512009 | 0.5294 | 0.5167712 | 1.4134731 |
| 1 | 3 | 0.5825308 | 0.0482* | 0.3396335 | 0.9956118 |
| 2 | 3 | 0.8048412 | 0.4136 | 0.4776748 | 1.3582396 |

**Proportional Hazards Fit tnm.stage 2=0-2**

Censored By: rfs.event

**Whole Model**

|  |  |
| --- | --- |
| Number of Events | 53 |
| Number of Censorings | 201 |
| Total Number | 254 |

| **Model** | **-LogLikelihood** | **ChiSquare** | **DF** | **Prob>Chisq** |
| --- | --- | --- | --- | --- |
| Difference | 5.6505 | 11.3011 | 10 | 0.3345 |
| Full | 272.4407 |  |  |  |
| Reduced | 278.0912 |  |  |  |

**Parameter Estimates**

| **Term** | **Estimate** | **Std Error** | **Lower 95%** | **Upper 95%** |  |
| --- | --- | --- | --- | --- | --- |
| RORb_43_Q[1-0] | 0.26807048 | 0.4442138 | -0.609566 | 1.156815 |  |
| RORb_43_Q[2-1] | -0.3608892 | 0.4548786 | -1.279039 | 0.5318462 |  |
| RORb_43_Q[3-2] | 0.89605924 | 0.3964789 | 0.1521121 | 1.7263419 |  |
| NRIP2_Q[1-0] | 0.24053374 | 0.3885837 | -0.513216 | 1.0256251 |  |
| NRIP2_Q[2-1] | -0.3555987 | 0.4166743 | -1.216597 | 0.4389792 |  |
| NRIP2_Q[3-2] | 0.29045884 | 0.427806 | -0.531286 | 1.1688873 |  |
| Sex[female] | -0.144713 | 0.1491073 | -0.447212 | 0.1410558 |  |
| Age at DX | 0.00534889 | 0.0116196 | -0.016831 | 0.0288294 |  |
| MMR.status[dMMR] | -0.229424 | 0.2336825 | -0.735782 | 0.1949729 |  |
| location[distal] | -0.0391595 | 0.1520638 | -0.33175 | 0.2674947 |  |

**Effect Likelihood Ratio Tests**

| **Source** | **Nparm** | **DF** | **L-R ChiSquare** | **Prob>ChiSq** |  |
| --- | --- | --- | --- | --- | --- |
| RORb_43_Q | 3 | 3 | 7.42676389 | 0.0595 |  |
| NRIP2_Q | 3 | 3 | 0.95298168 | 0.8126 |  |
| Sex | 1 | 1 | 0.96619906 | 0.3256 |  |
| Age at DX | 1 | 1 | 0.21475558 | 0.6431 |  |
| MMR.status | 1 | 1 | 1.04951721 | 0.3056 |  |
| location | 1 | 1 | 0.06589782 | 0.7974 |  |

**Baseline Survival at mean**

**Risk Ratios**

**Unit Risk Ratios**

Per unit change in regressor

| **Term** | **Risk Ratio** | **Lower 95%** | **Upper 95%** | **Reciprocal** |
| --- | --- | --- | --- | --- |
| Age at DX | 1.005363 | 0.98331 | 1.029249 | 0.9946654 |

**Range Risk Ratios**

Per change in regressor over entire range

| **Term** | **Risk Ratio** | **Lower 95%** | **Upper 95%** | **Reciprocal** |
| --- | --- | --- | --- | --- |
| Age at DX | 1.454151 | 0.307851 | 7.523708 | 0.6876867 |

**Risk Ratios for RORb_43_Q**

| **Level1** | **/Level2** | **Risk Ratio** | **Prob>Chisq** | **Lower 95%** | **Upper 95%** |
| --- | --- | --- | --- | --- | --- |
| 1 | 0 | 1.3074393 | 0.5460 | 0.5435869 | 3.1797896 |
| 2 | 0 | 0.9113587 | 0.8421 | 0.3576275 | 2.2893522 |
| 2 | 1 | 0.6970562 | 0.4261 | 0.2783046 | 1.7020718 |
| 3 | 0 | 2.2327645 | 0.0295* | 1.081031 | 4.9546161 |
| 3 | 1 | 1.7077386 | 0.1379 | 0.8455576 | 3.6642368 |
| 3 | 2 | 2.4499295 | 0.0175* | 1.1642908 | 5.6200574 |
| 0 | 1 | 0.7648539 | 0.5460 | 0.3144862 | 1.8396322 |
| 0 | 2 | 1.0972628 | 0.8421 | 0.4368048 | 2.7962057 |
| 1 | 2 | 1.4346045 | 0.4261 | 0.5875193 | 3.5931855 |
| 0 | 3 | 0.4478753 | 0.0295* | 0.201832 | 0.9250429 |
| 1 | 3 | 0.5855697 | 0.1379 | 0.2729081 | 1.1826515 |
| 2 | 3 | 0.408175 | 0.0175* | 0.1779341 | 0.858892 |

**Risk Ratios for NRIP2_Q**

| **Level1** | **/Level2** | **Risk Ratio** | **Prob>Chisq** | **Lower 95%** | **Upper 95%** |
| --- | --- | --- | --- | --- | --- |
| 1 | 0 | 1.2719279 | 0.5337 | 0.5985675 | 2.7888383 |
| 2 | 0 | 0.8913082 | 0.7983 | 0.3569669 | 2.1485795 |
| 2 | 1 | 0.7007538 | 0.3862 | 0.2962367 | 1.551123 |
| 3 | 0 | 1.1917155 | 0.6548 | 0.5524174 | 2.6270729 |
| 3 | 1 | 0.9369364 | 0.8571 | 0.4556575 | 1.9078882 |
| 3 | 2 | 1.3370408 | 0.4929 | 0.5878485 | 3.2184097 |
| 0 | 1 | 0.7862081 | 0.5337 | 0.3585722 | 1.6706554 |
| 0 | 2 | 1.1219463 | 0.7983 | 0.4654238 | 2.80138 |
| 1 | 2 | 1.4270348 | 0.3862 | 0.6446942 | 3.3756795 |
| 0 | 3 | 0.8391264 | 0.6548 | 0.3806518 | 1.8102254 |
| 1 | 3 | 1.0673083 | 0.8571 | 0.5241397 | 2.1946309 |
| 2 | 3 | 0.7479203 | 0.4929 | 0.3107125 | 1.7011185 |

**Risk Ratios for Sex**

| **Level1** | **/Level2** | **Risk Ratio** | **Prob>Chisq** | **Lower 95%** | **Upper 95%** |
| --- | --- | --- | --- | --- | --- |
| male | female | 1.3356605 | 0.3256 | 0.7541895 | 2.4459266 |
| female | male | 0.7486932 | 0.3256 | 0.408843 | 1.3259266 |

**Risk Ratios for MMR.status**

| **Level1** | **/Level2** | **Risk Ratio** | **Prob>Chisq** | **Lower 95%** | **Upper 95%** |
| --- | --- | --- | --- | --- | --- |
| pMMR | dMMR | 1.5822503 | 0.3056 | 0.6770936 | 4.3560395 |
| dMMR | pMMR | 0.6320113 | 0.3056 | 0.2295663 | 1.4769008 |

**Risk Ratios for location**

| **Level1** | **/Level2** | **Risk Ratio** | **Prob>Chisq** | **Lower 95%** | **Upper 95%** |
| --- | --- | --- | --- | --- | --- |
| proximal | distal | 1.0814676 | 0.7974 | 0.5856755 | 1.9415745 |
| distal | proximal | 0.9246694 | 0.7974 | 0.5150459 | 1.7074302 |

**Proportional Hazards Fit tnm.stage 2=3-4**

Censored By: rfs.event

**Whole Model**

|  |  |
| --- | --- |
| Number of Events | 114 |
| Number of Censorings | 142 |
| Total Number | 256 |

| **Model** | **-LogLikelihood** | **ChiSquare** | **DF** | **Prob>Chisq** |
| --- | --- | --- | --- | --- |
| Difference | 11.9976 | 23.9951 | 10 | 0.0076* |
| Full | 578.7696 |  |  |  |
| Reduced | 590.7672 |  |  |  |

**Parameter Estimates**

| **Term** | **Estimate** | **Std Error** | **Lower 95%** | **Upper 95%** |  |
| --- | --- | --- | --- | --- | --- |
| RORb_43_Q[1-0] | -0.3569504 | 0.2644473 | -0.884916 | 0.1574301 |  |
| RORb_43_Q[2-1] | 0.5099462 | 0.2806334 | -0.040396 | 1.0658598 |  |
| RORb_43_Q[3-2] | 0.16126031 | 0.2723947 | -0.376482 | 0.697282 |  |
| NRIP2_Q[1-0] | -0.2370801 | 0.26673 | -0.765794 | 0.285296 |  |
| NRIP2_Q[2-1] | -0.3811936 | 0.282088 | -0.938091 | 0.1737157 |  |
| NRIP2_Q[3-2] | 0.18142049 | 0.2693048 | -0.346076 | 0.7155477 |  |
| Sex[female] | -0.0861734 | 0.096538 | -0.277184 | 0.1022366 |  |
| Age at DX | -0.0010105 | 0.0078208 | -0.016253 | 0.0143872 |  |
| MMR.status[dMMR] | -0.6679207 | 0.2605879 | -1.267469 | -0.218706 |  |
| location[distal] | 0.11890072 | 0.1033877 | -0.080019 | 0.3264533 |  |

**Effect Likelihood Ratio Tests**

| **Source** | **Nparm** | **DF** | **L-R ChiSquare** | **Prob>ChiSq** |  |
| --- | --- | --- | --- | --- | --- |
| RORb_43_Q | 3 | 3 | 6.34786738 | 0.0959 |  |
| NRIP2_Q | 3 | 3 | 5.69619901 | 0.1274 |  |
| Sex | 1 | 1 | 0.80092299 | 0.3708 |  |
| Age at DX | 1 | 1 | 0.01668207 | 0.8972 |  |
| MMR.status | 1 | 1 | 9.61199384 | 0.0019* |  |
| location | 1 | 1 | 1.35377484 | 0.2446 |  |

**Baseline Survival at mean**

**Risk Ratios**

**Unit Risk Ratios**

Per unit change in regressor

| **Term** | **Risk Ratio** | **Lower 95%** | **Upper 95%** | **Reciprocal** |
| --- | --- | --- | --- | --- |
| Age at DX | 0.99899 | 0.983879 | 1.014491 | 1.001011 |

**Range Risk Ratios**

Per change in regressor over entire range

| **Term** | **Risk Ratio** | **Lower 95%** | **Upper 95%** | **Reciprocal** |
| --- | --- | --- | --- | --- |
| Age at DX | 0.927015 | 0.295537 | 2.941848 | 1.0787308 |

**Risk Ratios for RORb_43_Q**

| **Level1** | **/Level2** | **Risk Ratio** | **Prob>Chisq** | **Lower 95%** | **Upper 95%** |
| --- | --- | --- | --- | --- | --- |
| 1 | 0 | 0.6998072 | 0.1743 | 0.412749 | 1.1704989 |
| 2 | 0 | 1.1653201 | 0.5628 | 0.6907054 | 1.9517088 |
| 2 | 1 | 1.6652016 | 0.0692 | 0.9604093 | 2.9033342 |
| 3 | 0 | 1.3692404 | 0.2405 | 0.8073828 | 2.3013174 |
| 3 | 1 | 1.9565965 | 0.0179* | 1.1235487 | 3.4199996 |
| 3 | 2 | 1.1749908 | 0.5542 | 0.6862713 | 2.0082868 |
| 0 | 1 | 1.428965 | 0.1743 | 0.8543365 | 2.4227798 |
| 0 | 2 | 0.8581333 | 0.5628 | 0.5123715 | 1.4477952 |
| 1 | 2 | 0.6005279 | 0.0692 | 0.3444316 | 1.0412228 |
| 0 | 3 | 0.730332 | 0.2405 | 0.4345337 | 1.2385698 |
| 1 | 3 | 0.5110916 | 0.0179* | 0.2923977 | 0.8900371 |
| 2 | 3 | 0.8510705 | 0.5542 | 0.4979368 | 1.4571497 |

**Risk Ratios for NRIP2_Q**

| **Level1** | **/Level2** | **Risk Ratio** | **Prob>Chisq** | **Lower 95%** | **Upper 95%** |
| --- | --- | --- | --- | --- | --- |
| 1 | 0 | 0.7889281 | 0.3733 | 0.4649646 | 1.3301557 |
| 2 | 0 | 0.5388739 | 0.0224* | 0.3144881 | 0.9159812 |
| 2 | 1 | 0.6830456 | 0.1772 | 0.3913743 | 1.1897172 |
| 3 | 0 | 0.6460662 | 0.0933 | 0.3870819 | 1.0767561 |
| 3 | 1 | 0.8189165 | 0.4619 | 0.481268 | 1.3995485 |
| 3 | 2 | 1.1989192 | 0.4999 | 0.707459 | 2.0453066 |
| 0 | 1 | 1.2675427 | 0.3733 | 0.7517917 | 2.1507014 |
| 0 | 2 | 1.8557218 | 0.0224* | 1.0917255 | 3.1797702 |
| 1 | 2 | 1.464031 | 0.1772 | 0.8405359 | 2.5550986 |
| 0 | 3 | 1.5478289 | 0.0933 | 0.9287154 | 2.5834325 |
| 1 | 3 | 1.2211257 | 0.4619 | 0.7145161 | 2.0778446 |
| 2 | 3 | 0.8340846 | 0.4999 | 0.4889242 | 1.4135095 |

**Risk Ratios for Sex**

| **Level1** | **/Level2** | **Risk Ratio** | **Prob>Chisq** | **Lower 95%** | **Upper 95%** |
| --- | --- | --- | --- | --- | --- |
| male | female | 1.1880897 | 0.3708 | 0.8150765 | 1.7408395 |
| female | male | 0.8416873 | 0.3708 | 0.5744355 | 1.2268786 |

**Risk Ratios for MMR.status**

| **Level1** | **/Level2** | **Risk Ratio** | **Prob>Chisq** | **Lower 95%** | **Upper 95%** |
| --- | --- | --- | --- | --- | --- |
| pMMR | dMMR | 3.8031943 | 0.0019* | 1.5486944 | 12.615653 |
| dMMR | pMMR | 0.2629369 | 0.0019* | 0.0792666 | 0.6457052 |

**Risk Ratios for location**

| **Level1** | **/Level2** | **Risk Ratio** | **Prob>Chisq** | **Lower 95%** | **Upper 95%** |
| --- | --- | --- | --- | --- | --- |
| proximal | distal | 0.7883592 | 0.2446 | 0.5205307 | 1.1735544 |
| distal | proximal | 1.2684573 | 0.2446 | 0.8521122 | 1.9211165 |

**Proportional Hazards Fit chemotherapy.adjuvant=N**

Censored By: rfs.event

**Whole Model**

|  |  |
| --- | --- |
| Number of Events | 70 |
| Number of Censorings | 207 |
| Total Number | 277 |

| **Model** | **-LogLikelihood** | **ChiSquare** | **DF** | **Prob>Chisq** |
| --- | --- | --- | --- | --- |
| Difference | 30.8022 | 61.6044 | 11 | <.0001* |
| Full | 339.7898 |  |  |  |
| Reduced | 370.5920 |  |  |  |

**Parameter Estimates**

| **Term** | **Estimate** | **Std Error** | **Lower 95%** | **Upper 95%** |  |
| --- | --- | --- | --- | --- | --- |
| RORb_43_Q[1-0] | -0.3316926 | 0.3844481 | -1.105534 | 0.4140788 |  |
| RORb_43_Q[2-1] | -0.3872581 | 0.4180141 | -1.225956 | 0.4331832 |  |
| RORb_43_Q[3-2] | 1.14806029 | 0.366045 | 0.4576778 | 1.9069434 |  |
| NRIP2_Q[1-0] | -0.1006909 | 0.3417852 | -0.76809 | 0.5810759 |  |
| NRIP2_Q[2-1] | -0.4185192 | 0.3776112 | -1.188959 | 0.3046173 |  |
| NRIP2_Q[3-2] | 0.53048526 | 0.3864783 | -0.213708 | 1.3155062 |  |
| tnm.stage | 1.13796834 | 0.1828049 | 0.7792117 | 1.4971882 |  |
| Sex[female] | -0.3921998 | 0.1425264 | -0.681176 | -0.120151 |  |
| Age at DX | 0.00800999 | 0.010277 | -0.011363 | 0.0290546 |  |
| location[distal] | 0.00588887 | 0.1311514 | -0.249438 | 0.266639 |  |
| MMR.status[dMMR] | -0.5382685 | 0.2260038 | -1.032201 | -0.130958 |  |

**Effect Likelihood Ratio Tests**

| **Source** | **Nparm** | **DF** | **L-R ChiSquare** | **Prob>ChiSq** |  |
| --- | --- | --- | --- | --- | --- |
| RORb_43_Q | 3 | 3 | 11.6043547 | 0.0089* |  |
| NRIP2_Q | 3 | 3 | 2.37573954 | 0.4982 |  |
| tnm.stage | 1 | 1 | 37.8335458 | <.0001* |  |
| Sex | 1 | 1 | 8.13840695 | 0.0043* |  |
| Age at DX | 1 | 1 | 0.62781994 | 0.4282 |  |
| location | 1 | 1 | 0.00201709 | 0.9642 |  |
| MMR.status | 1 | 1 | 7.04586226 | 0.0079* |  |

**Baseline Survival at mean**

**Risk Ratios**

**Unit Risk Ratios**

Per unit change in regressor

| **Term** | **Risk Ratio** | **Lower 95%** | **Upper 95%** | **Reciprocal** |
| --- | --- | --- | --- | --- |
| tnm.stage | 3.120422 | 2.179753 | 4.469105 | 0.3204694 |
| Age at DX | 1.008042 | 0.988702 | 1.029481 | 0.992022 |

**Range Risk Ratios**

Per change in regressor over entire range

| **Term** | **Risk Ratio** | **Lower 95%** | **Upper 95%** | **Reciprocal** |
| --- | --- | --- | --- | --- |
| tnm.stage | 94.80986 | 22.57509 | 398.9167 | 0.0105474 |
| Age at DX | 1.751897 | 0.451406 | 7.643265 | 0.5708098 |

**Risk Ratios for RORb_43_Q**

| **Level1** | **/Level2** | **Risk Ratio** | **Prob>Chisq** | **Lower 95%** | **Upper 95%** |
| --- | --- | --- | --- | --- | --- |
| 1 | 0 | 0.7177079 | 0.3852 | 0.3310339 | 1.5129764 |
| 2 | 0 | 0.4872633 | 0.0586 | 0.2202369 | 1.0261696 |
| 2 | 1 | 0.6789158 | 0.3531 | 0.2934771 | 1.5421587 |
| 3 | 0 | 1.5358894 | 0.1524 | 0.8540176 | 2.8174224 |
| 3 | 1 | 2.1399922 | 0.0299* | 1.0755749 | 4.4663874 |
| 3 | 2 | 3.1520729 | 0.0009* | 1.5803998 | 6.7324787 |
| 0 | 1 | 1.3933245 | 0.3852 | 0.6609488 | 3.0208385 |
| 0 | 2 | 2.0522786 | 0.0586 | 0.9744977 | 4.540565 |
| 1 | 2 | 1.4729366 | 0.3531 | 0.6484417 | 3.4074207 |
| 0 | 3 | 0.6510886 | 0.1524 | 0.3549344 | 1.1709361 |
| 1 | 3 | 0.4672914 | 0.0299* | 0.2238946 | 0.9297354 |
| 2 | 3 | 0.3172515 | 0.0009* | 0.1485337 | 0.6327513 |

**Risk Ratios for NRIP2_Q**

| **Level1** | **/Level2** | **Risk Ratio** | **Prob>Chisq** | **Lower 95%** | **Upper 95%** |
| --- | --- | --- | --- | --- | --- |
| 1 | 0 | 0.9042125 | 0.7687 | 0.4638985 | 1.787961 |
| 2 | 0 | 0.5949903 | 0.1947 | 0.262582 | 1.3015108 |
| 2 | 1 | 0.6580205 | 0.2604 | 0.3045382 | 1.3561059 |
| 3 | 0 | 1.0113389 | 0.9736 | 0.5173864 | 1.990601 |
| 3 | 1 | 1.1184749 | 0.7357 | 0.5801518 | 2.1456393 |
| 3 | 2 | 1.6997569 | 0.1638 | 0.8075843 | 3.7266369 |
| 0 | 1 | 1.1059348 | 0.7687 | 0.5592963 | 2.1556441 |
| 0 | 2 | 1.6806996 | 0.1947 | 0.7683378 | 3.8083346 |
| 1 | 2 | 1.5197095 | 0.2604 | 0.7374055 | 3.2836608 |
| 0 | 3 | 0.9887882 | 0.9736 | 0.5023609 | 1.9327913 |
| 1 | 3 | 0.8940746 | 0.7357 | 0.4660615 | 1.7236869 |
| 2 | 3 | 0.5883194 | 0.1638 | 0.2683385 | 1.2382608 |

**Risk Ratios for Sex**

| **Level1** | **/Level2** | **Risk Ratio** | **Prob>Chisq** | **Lower 95%** | **Upper 95%** |
| --- | --- | --- | --- | --- | --- |
| male | female | 2.191091 | 0.0043* | 1.2716341 | 3.9053717 |
| female | male | 0.4563936 | 0.0043* | 0.2560576 | 0.7863897 |

**Risk Ratios for location**

| **Level1** | **/Level2** | **Risk Ratio** | **Prob>Chisq** | **Lower 95%** | **Upper 95%** |
| --- | --- | --- | --- | --- | --- |
| proximal | distal | 0.9882914 | 0.9642 | 0.5866787 | 1.6468679 |
| distal | proximal | 1.0118474 | 0.9642 | 0.6072132 | 1.7045106 |

**Risk Ratios for MMR.status**

| **Level1** | **/Level2** | **Risk Ratio** | **Prob>Chisq** | **Lower 95%** | **Upper 95%** |
| --- | --- | --- | --- | --- | --- |
| pMMR | dMMR | 2.9344997 | 0.0079* | 1.2994165 | 7.8805788 |
| dMMR | pMMR | 0.3407736 | 0.0079* | 0.1268942 | 0.7695762 |

**Proportional Hazards Fit chemotherapy.adjuvant=Y**

Censored By: rfs.event

**Whole Model**

|  |  |
| --- | --- |
| Number of Events | 82 |
| Number of Censorings | 135 |
| Total Number | 217 |

| **Model** | **-LogLikelihood** | **ChiSquare** | **DF** | **Prob>Chisq** |
| --- | --- | --- | --- | --- |
| Difference | 8.5196 | 17.0393 | 11 | 0.1067 |
| Full | 407.8565 |  |  |  |
| Reduced | 416.3762 |  |  |  |

**Parameter Estimates**

| **Term** | **Estimate** | **Std Error** | **Lower 95%** | **Upper 95%** |  |
| --- | --- | --- | --- | --- | --- |
| RORb_43_Q[1-0] | -0.2689974 | 0.3271347 | -0.923768 | 0.3688299 |  |
| RORb_43_Q[2-1] | 0.50953305 | 0.333777 | -0.142427 | 1.1762474 |  |
| RORb_43_Q[3-2] | 0.08824312 | 0.3123553 | -0.527321 | 0.7055203 |  |
| NRIP2_Q[1-0] | 0.13044142 | 0.3343806 | -0.524984 | 0.7966723 |  |
| NRIP2_Q[2-1] | -0.4319617 | 0.3298846 | -1.084709 | 0.2178927 |  |
| NRIP2_Q[3-2] | 0.19455198 | 0.304345 | -0.397258 | 0.804283 |  |
| tnm.stage | 0.60765092 | 0.2097875 | 0.1972257 | 1.0203837 |  |
| Sex[female] | 0.03430413 | 0.1142816 | -0.191653 | 0.2579127 |  |
| Age at DX | 0.0013205 | 0.0099758 | -0.018143 | 0.0209173 |  |
| location[distal] | 0.19675109 | 0.1313058 | -0.052639 | 0.4645817 |  |
| MMR.status[dMMR] | -0.182829 | 0.271616 | -0.797999 | 0.2944759 |  |

**Effect Likelihood Ratio Tests**

| **Source** | **Nparm** | **DF** | **L-R ChiSquare** | **Prob>ChiSq** |  |
| --- | --- | --- | --- | --- | --- |
| RORb_43_Q | 3 | 3 | 4.02326113 | 0.2590 |  |
| NRIP2_Q | 3 | 3 | 1.8099614 | 0.6128 |  |
| tnm.stage | 1 | 1 | 8.42401071 | 0.0037* |  |
| Sex | 1 | 1 | 0.08994438 | 0.7642 |  |
| Age at DX | 1 | 1 | 0.01753054 | 0.8947 |  |
| location | 1 | 1 | 2.36207355 | 0.1243 |  |
| MMR.status | 1 | 1 | 0.49369316 | 0.4823 |  |

**Baseline Survival at mean**

**Risk Ratios**

**Unit Risk Ratios**

Per unit change in regressor

| **Term** | **Risk Ratio** | **Lower 95%** | **Upper 95%** | **Reciprocal** |
| --- | --- | --- | --- | --- |
| tnm.stage | 1.836113 | 1.218019 | 2.774259 | 0.5446287 |
| Age at DX | 1.001321 | 0.982021 | 1.021138 | 0.9986804 |

**Range Risk Ratios**

Per change in regressor over entire range

| **Term** | **Risk Ratio** | **Lower 95%** | **Upper 95%** | **Reciprocal** |
| --- | --- | --- | --- | --- |
| tnm.stage | 3.371312 | 1.48357 | 7.696514 | 0.2966205 |
| Age at DX | 1.104108 | 0.256476 | 4.800885 | 0.9057088 |

**Risk Ratios for RORb_43_Q**

| **Level1** | **/Level2** | **Risk Ratio** | **Prob>Chisq** | **Lower 95%** | **Upper 95%** |
| --- | --- | --- | --- | --- | --- |
| 1 | 0 | 0.7641452 | 0.4090 | 0.3970203 | 1.4460416 |
| 2 | 0 | 1.2719303 | 0.4504 | 0.6783141 | 2.3804016 |
| 2 | 1 | 1.6645138 | 0.1254 | 0.8672507 | 3.2421849 |
| 3 | 0 | 1.3892704 | 0.2941 | 0.7497083 | 2.5754704 |
| 3 | 1 | 1.8180712 | 0.0675 | 0.957713 | 3.5105247 |
| 3 | 2 | 1.0922536 | 0.7775 | 0.5901837 | 2.0249 |
| 0 | 1 | 1.3086518 | 0.4090 | 0.6915431 | 2.5187631 |
| 0 | 2 | 0.7862066 | 0.4504 | 0.4200972 | 1.4742432 |
| 1 | 2 | 0.600776 | 0.1254 | 0.308434 | 1.1530691 |
| 0 | 3 | 0.7198023 | 0.2941 | 0.3882786 | 1.3338521 |
| 1 | 3 | 0.5500335 | 0.0675 | 0.2848577 | 1.0441542 |
| 2 | 3 | 0.9155383 | 0.7775 | 0.4938515 | 1.6943878 |

**Risk Ratios for NRIP2_Q**

| **Level1** | **/Level2** | **Risk Ratio** | **Prob>Chisq** | **Lower 95%** | **Upper 95%** |
| --- | --- | --- | --- | --- | --- |
| 1 | 0 | 1.1393312 | 0.6961 | 0.5915647 | 2.2181472 |
| 2 | 0 | 0.7396928 | 0.3861 | 0.3743874 | 1.4720387 |
| 2 | 1 | 0.6492342 | 0.1912 | 0.3380001 | 1.2434537 |
| 3 | 0 | 0.8985542 | 0.7393 | 0.4835274 | 1.7126707 |
| 3 | 1 | 0.7886681 | 0.4379 | 0.435676 | 1.4475737 |
| 3 | 2 | 1.2147666 | 0.5209 | 0.6721609 | 2.2350934 |
| 0 | 1 | 0.8777079 | 0.6961 | 0.4508267 | 1.6904323 |
| 0 | 2 | 1.3519125 | 0.3861 | 0.67933 | 2.6710298 |
| 1 | 2 | 1.5402761 | 0.1912 | 0.8042117 | 2.9585787 |
| 0 | 3 | 1.112899 | 0.7393 | 0.5838834 | 2.068135 |
| 1 | 3 | 1.2679605 | 0.4379 | 0.6908111 | 2.2952838 |
| 2 | 3 | 0.8232034 | 0.5209 | 0.4474086 | 1.4877391 |

**Risk Ratios for Sex**

| **Level1** | **/Level2** | **Risk Ratio** | **Prob>Chisq** | **Lower 95%** | **Upper 95%** |
| --- | --- | --- | --- | --- | --- |
| male | female | 0.9336924 | 0.7642 | 0.5970076 | 1.4671272 |
| female | male | 1.0710166 | 0.7642 | 0.6816042 | 1.6750204 |

**Risk Ratios for location**

| **Level1** | **/Level2** | **Risk Ratio** | **Prob>Chisq** | **Lower 95%** | **Upper 95%** |
| --- | --- | --- | --- | --- | --- |
| proximal | distal | 0.6746898 | 0.1243 | 0.3948839 | 1.1110188 |
| distal | proximal | 1.4821625 | 0.1243 | 0.9000748 | 2.5323898 |

**Risk Ratios for MMR.status**

| **Level1** | **/Level2** | **Risk Ratio** | **Prob>Chisq** | **Lower 95%** | **Upper 95%** |
| --- | --- | --- | --- | --- | --- |
| pMMR | dMMR | 1.4414622 | 0.4823 | 0.5549086 | 4.9332509 |
| dMMR | pMMR | 0.69374 | 0.4823 | 0.2027061 | 1.8020986 |
